# Supplementary material for: M6Allele: a toolkit for detection of allele-specific RNA N6-methyladenosine modifications
Source: Gigascience. 2025 May 19;14:giaf040. doi: 10.1093/gigascience/giaf040 (PMC12087454; doi:10.1093/gigascience/giaf040)
Supplement: giaf040_Supplemental_Files [file giaf040_supplemental_files.zip › Figure 4.pdf]

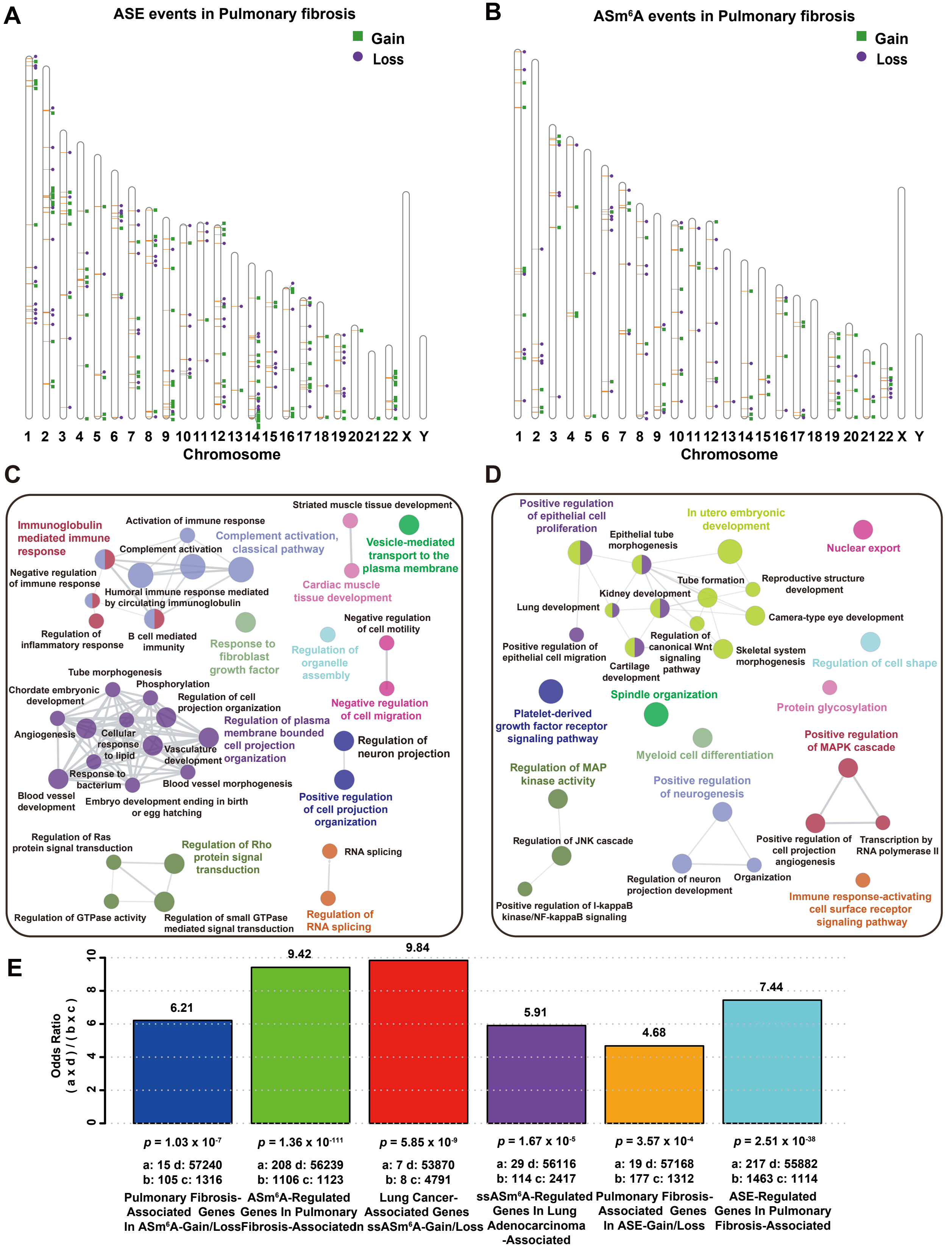
A
ASE events in Pulmonary fibrosis
Gain
Loss
1 2 3 4 5 6 7 8 9 10 11 12 13 14 15 16 17 18 19 20 21 22 X Y
Chromosome
B
ASm6A events in Pulmonary fibrosis
Gain
Loss
1 2 3 4 5 6 7 8 9 10 11 12 13 14 15 16 17 18 19 20 21 22 X Y
Chromosome
C
Immunoglobulin mediated immune response
Activation of immune response
Complement activation
Complement activation, classical pathway
Striated muscle tissue development
Vesicle-mediated transport to the plasma membrane
Negative regulation of immune response
Humoral immune response mediated by circulating immunoglobulin
Cardiac muscle tissue development
Regulation of inflammatory response
B cell mediated immunity
Response to fibroblast growth factor
Regulation of organelle assembly
Negative regulation of cell motility
Tube morphogenesis
Phosphorylation
Chordate embryonic development
Regulation of cell projection organization
Negative regulation of cell migration
Regulation of plasma membrane bounded cell projection organization
Angiogenesis
Cellular response to lipid
Vasculature development
Regulation of neuron projection
Response to bacterium
Blood vessel morphogenesis
Blood vessel development
Embryo development ending in birth or egg hatching
Positive regulation of cell projection organization
Regulation of Ras protein signal transduction
Regulation of Rho protein signal transduction
RNA splicing
Regulation of GTPase activity
Regulation of small GTPase mediated signal transduction
Regulation of RNA splicing
D
Positive regulation of epithelial cell proliferation
Epithelial tube morphogenesis
In utero embryonic development
Nuclear export
Tube formation
Reproductive structure development
Kidney development
Lung development
Camera-type eye development
Positive regulation of epithelial cell migration
Regulation of canonical Wnt signaling pathway
Skeletal system morphogenesis
Regulation of cell shape
Cartilage development
Platelet-derived growth factor receptor signaling pathway
Spindle organization
Protein glycosylation
Myeloid cell differentiation
Positive regulation of MAPK cascade
Regulation of MAP kinase activity
Positive regulation of neurogenesis
Regulation of JNK cascade
Positive regulation of cell projection angiogenesis
Transcription by RNA polymerase II
Regulation of neuron projection development
Organization
Immune response-activating cell surface receptor signaling pathway
Positive regulation of I-kappaB kinase/NF-kappaB signaling
E
Odds Ratio
(a x d) / (b x c)
0 2 4 6 8 10
6.21
9.42
9.84
5.91
4.68
7.44
p = 1.03 x 10-7
a: 15 d: 57240
b: 105 c: 1316
Pulmonary Fibrosis-Associated Genes In ASm6A-Gain/Loss
p = 1.36 x 10-111
a: 208 d: 56239
b: 1106 c: 1123
ASm6A-Regulated Genes In Pulmonary Fibrosis-Associated
p = 5.85 x 10-9
a: 7 d: 53870
b: 8 c: 4791
Lung Cancer-Associated Genes in ssASm6A-Gain/Loss
p = 1.67 x 10-5
a: 29 d: 56116
b: 114 c: 2417
ssASm6A-Regulated Genes In Lung Adenocarcinoma -Associated
p = 3.57 x 10-4
a: 19 d: 57168
b: 177 c: 1312
Pulmonary Fibrosis-Associated Genes In ASE-Gain/Loss
p = 2.51 x 10-38
a: 217 d: 55882
b: 1463 c: 1114
ASE-Regulated Genes In Pulmonary Fibrosis-Associated
