# Supplementary material for: M6Allele: a toolkit for detection of allele-specific RNA N6-methyladenosine modifications
Source: Gigascience. 2025 May 19;14:giaf040. doi: 10.1093/gigascience/giaf040 (PMC12087454; doi:10.1093/gigascience/giaf040)
Supplement: giaf040_Supplemental_Files [file giaf040_supplemental_files.zip › Figure S10.pdf]

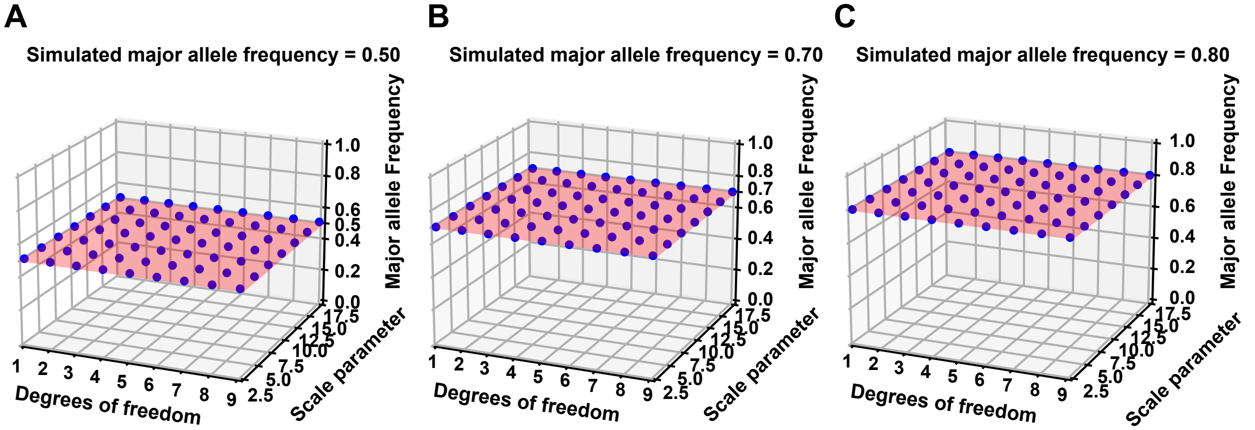
A
Simulated major allele frequency = 0.50
B
Simulated major allele frequency = 0.70
C
Simulated major allele frequency = 0.80
Major allele Frequency
Degrees of freedom
Scale parameter
1.0
0.8
0.7
0.6
0.5
0.4
0.2
0.0
1 2 3 4 5 6 7 8 9
2.5 5.0 7.5 10.0 12.5 15.0 17.5
