# Supplementary figures and images for: M6Allele: a toolkit for detection of allele-specific RNA N6-methyladenosine modifications
Source: Gigascience. 2025 May 19;14:giaf040. doi: 10.1093/gigascience/giaf040 (PMC12087454; doi:10.1093/gigascience/giaf040)

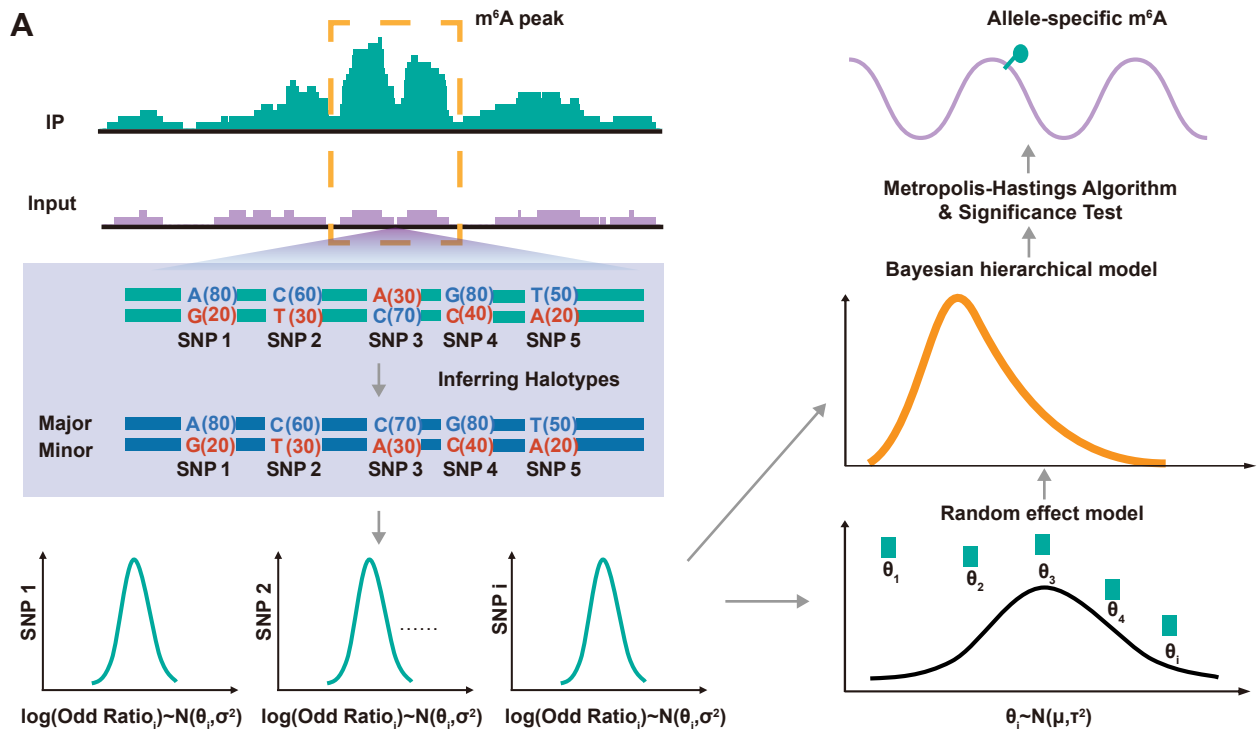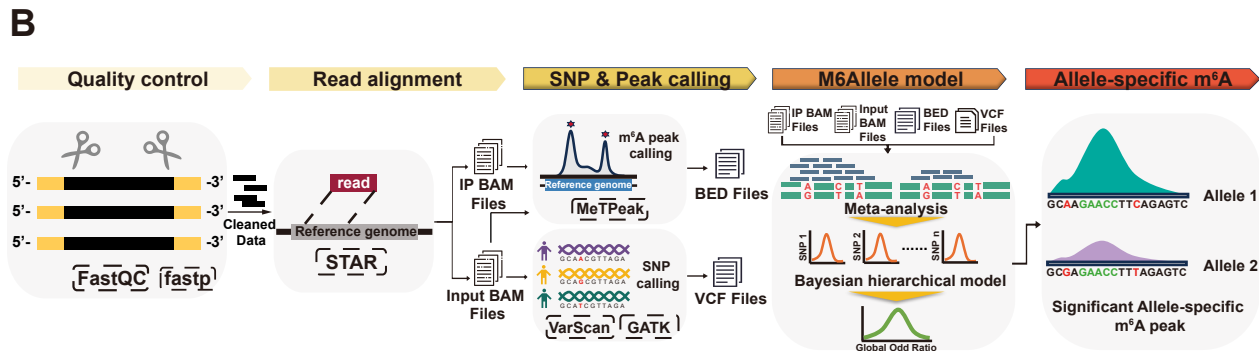

Supplement: giaf040_Supplemental_Files [file giaf040_supplemental_files.zip › Figure 1.pdf]

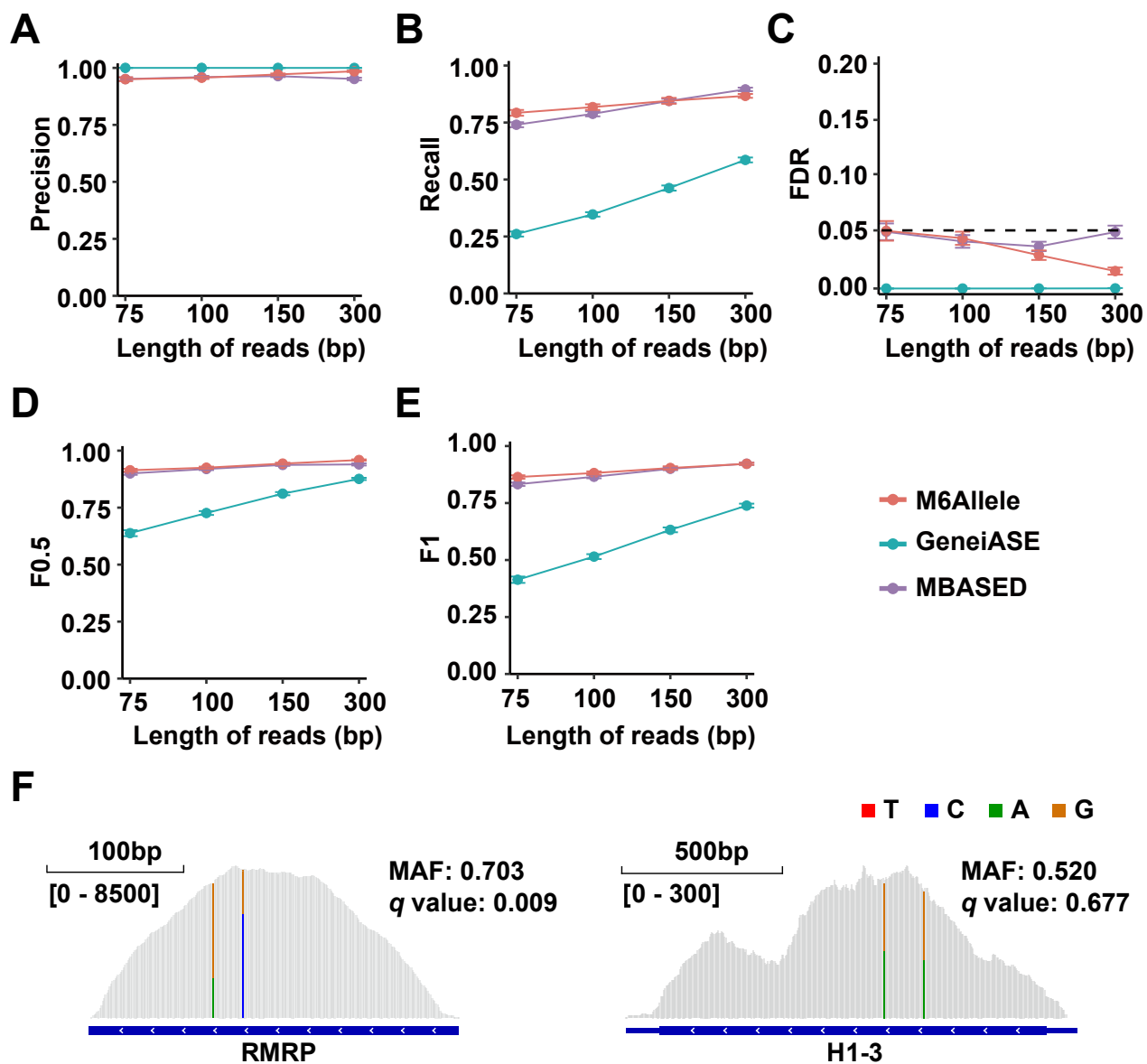

Supplement: giaf040_Supplemental_Files [file giaf040_supplemental_files.zip › Figure 2.pdf]

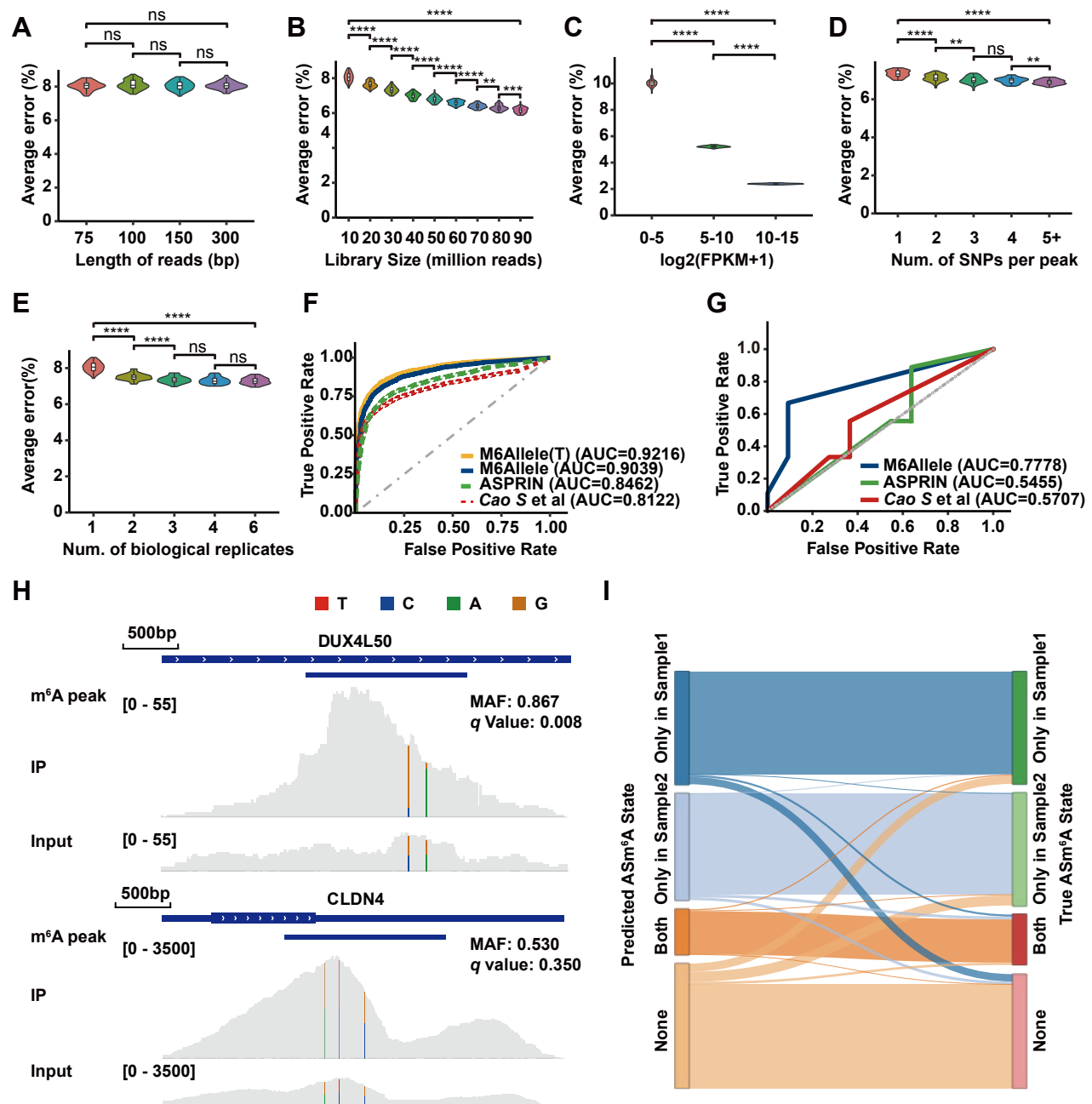

Supplement: giaf040_Supplemental_Files [file giaf040_supplemental_files.zip › Figure 3.pdf]

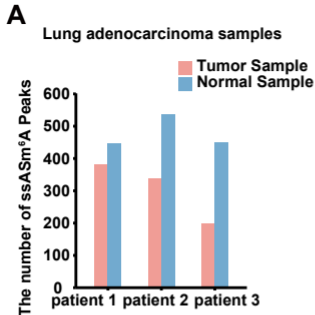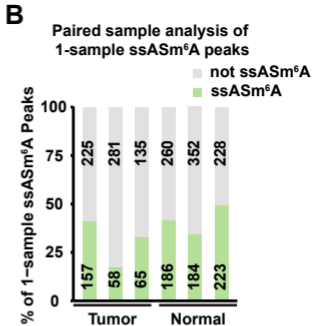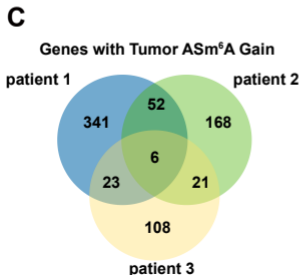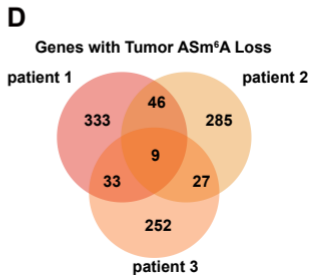

Supplement: giaf040_Supplemental_Files [file giaf040_supplemental_files.zip › Figure 5.pdf]

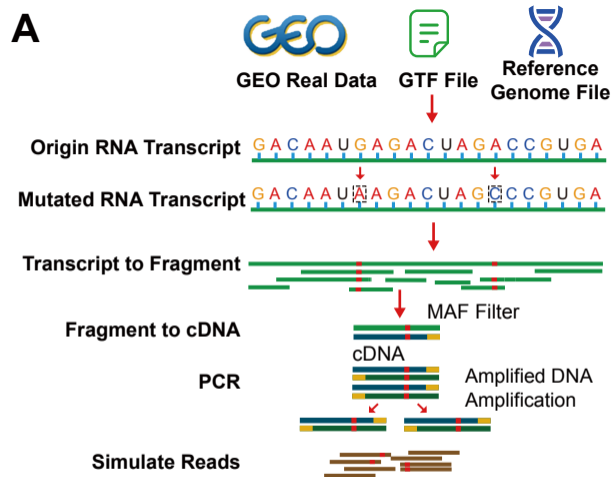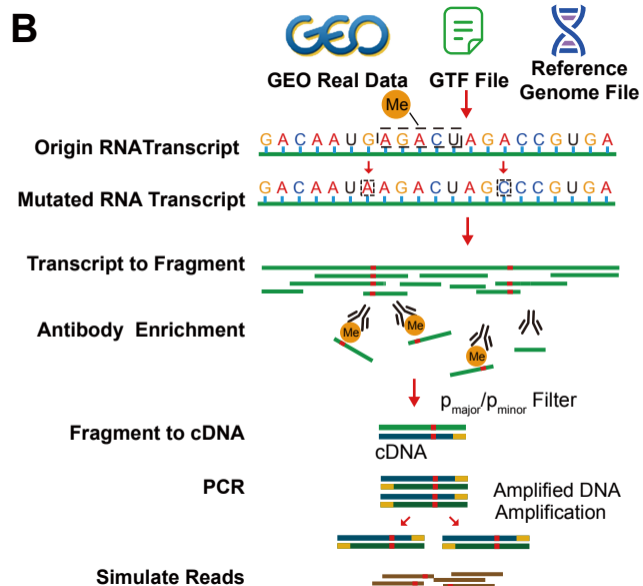

Supplement: giaf040_Supplemental_Files [file giaf040_supplemental_files.zip › Figure S1.pdf]

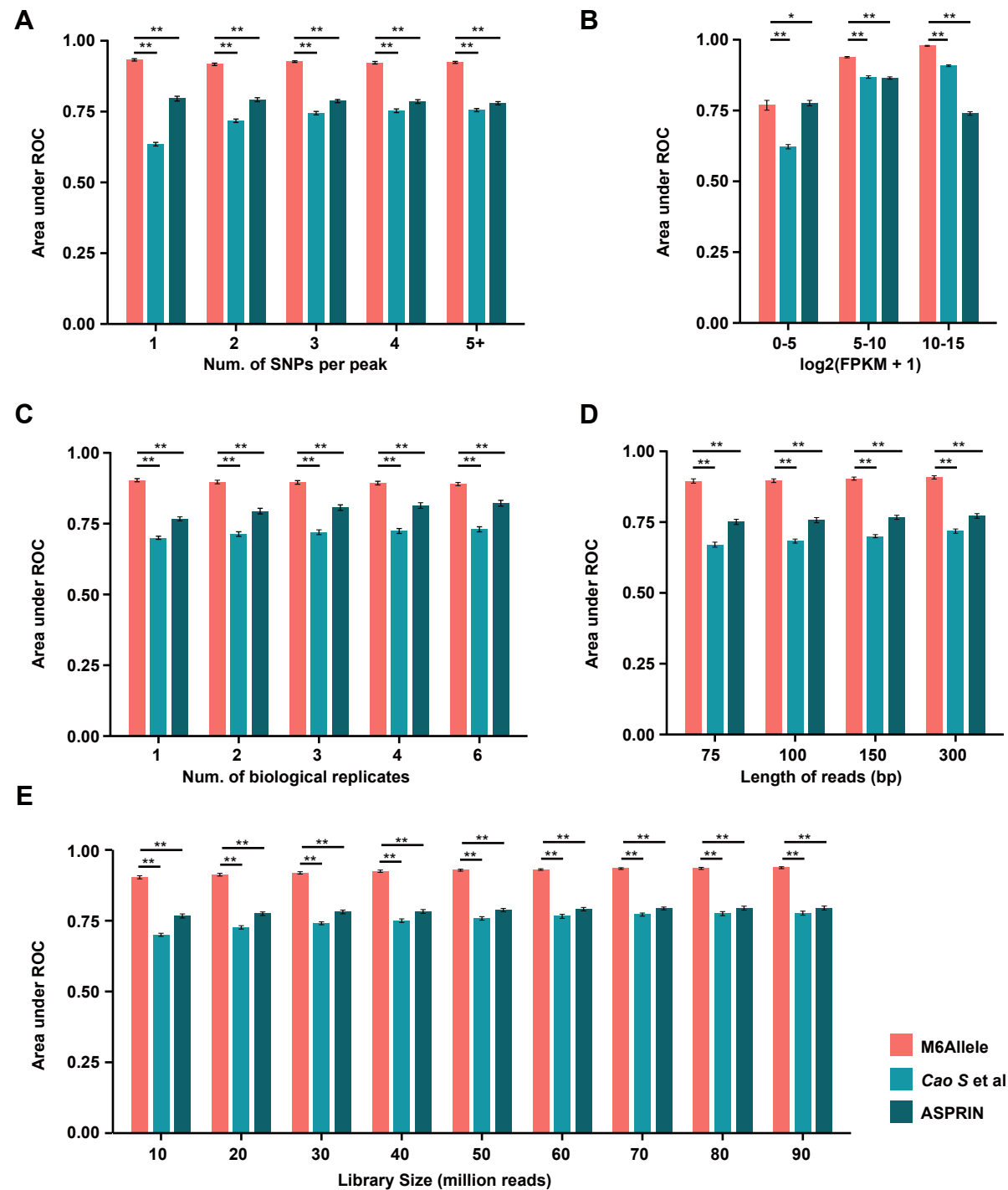

Supplement: giaf040_Supplemental_Files [file giaf040_supplemental_files.zip › Figure S2.pdf]

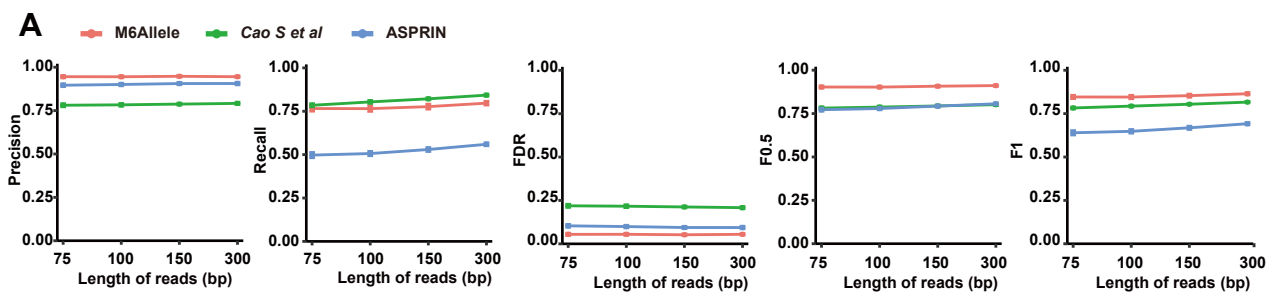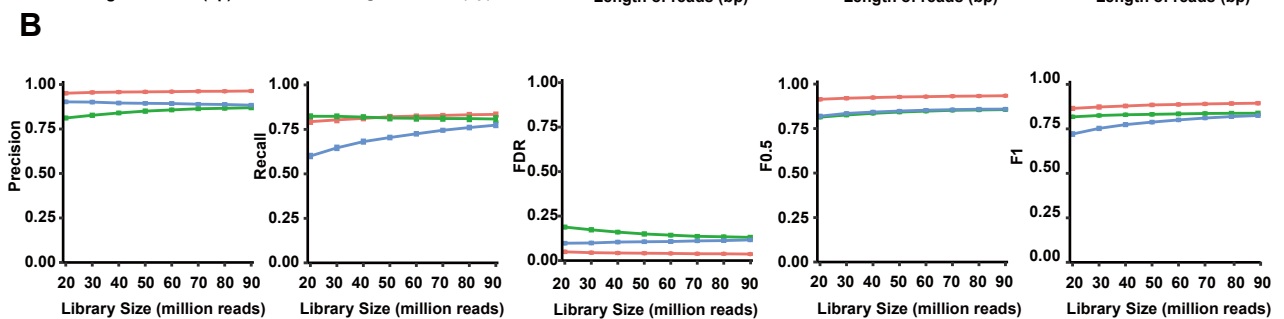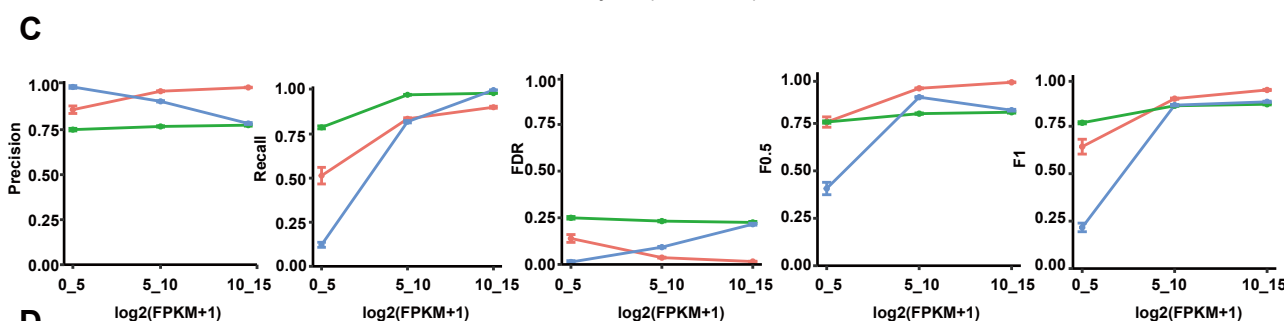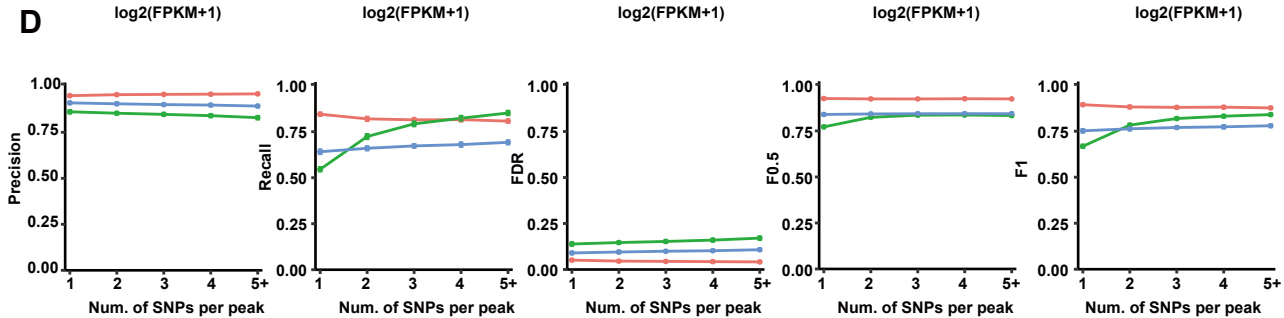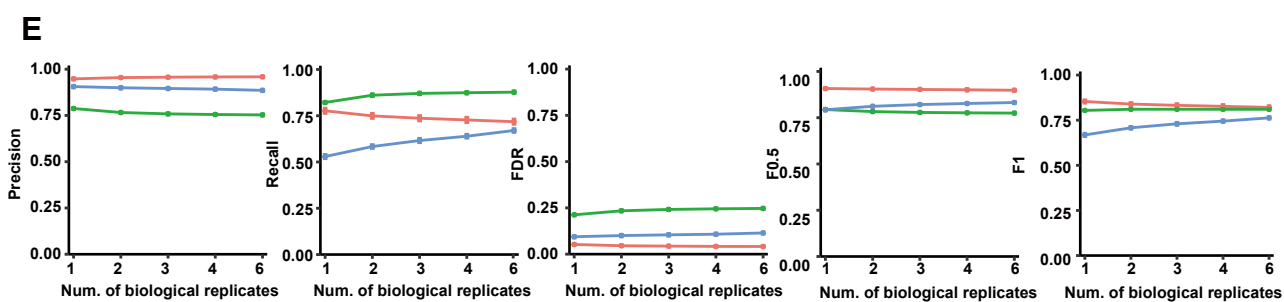

Supplement: giaf040_Supplemental_Files [file giaf040_supplemental_files.zip › Figure S3.pdf]

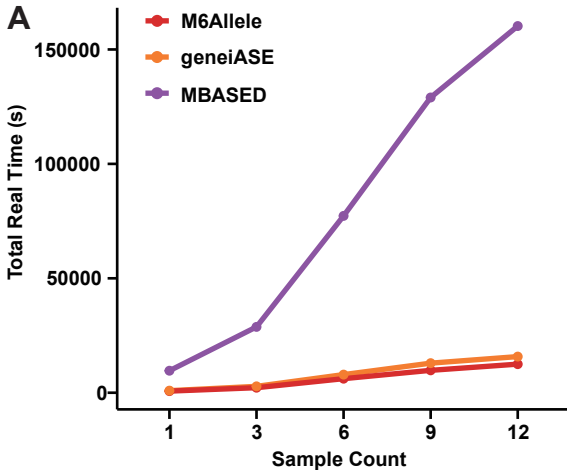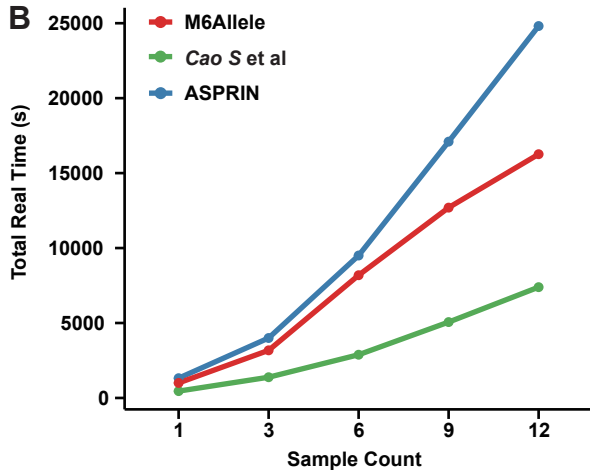

Supplement: giaf040_Supplemental_Files [file giaf040_supplemental_files.zip › Figure S4.pdf]

**A**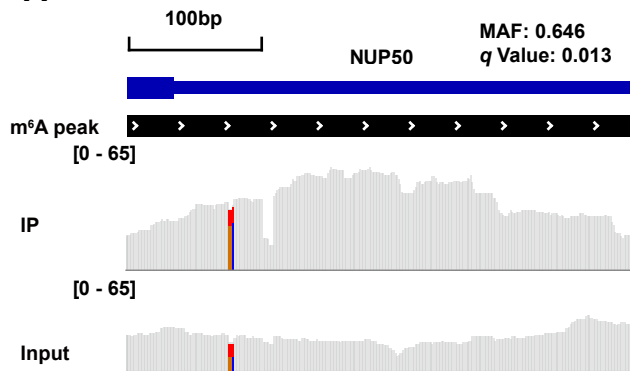**B**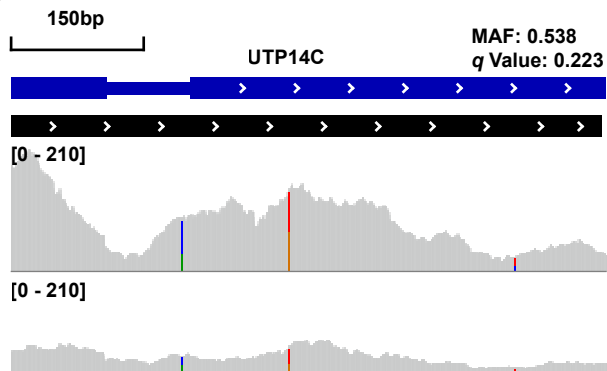**C**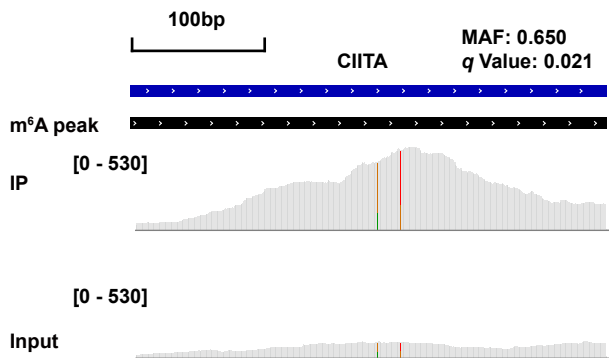**D**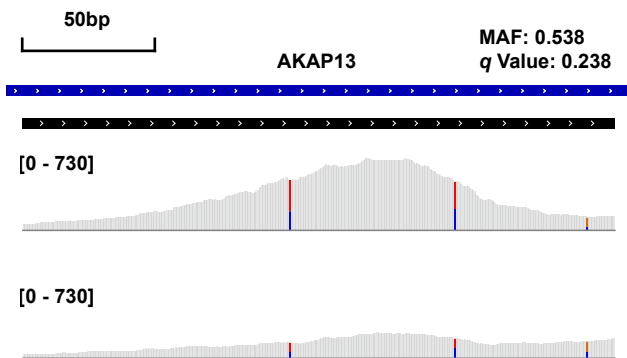

Supplement: giaf040_Supplemental_Files [file giaf040_supplemental_files.zip › Figure S5.pdf]

**A**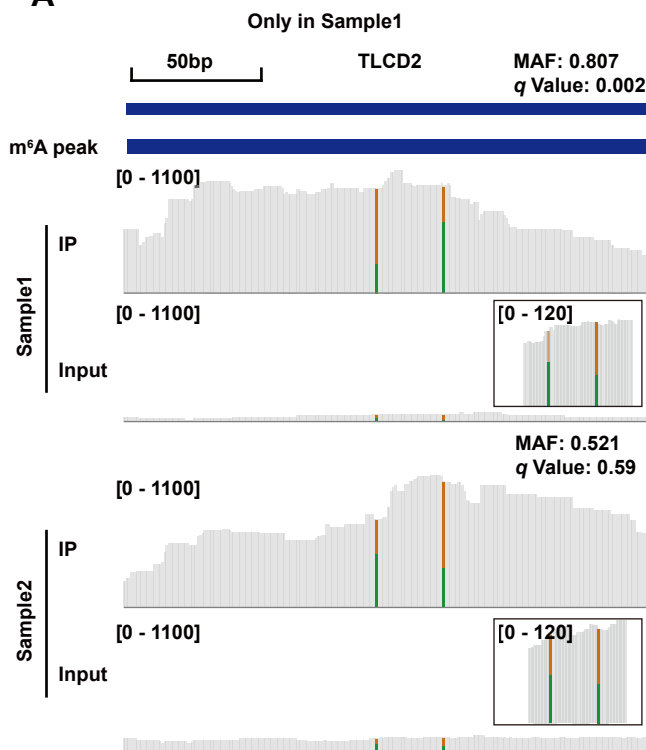**B**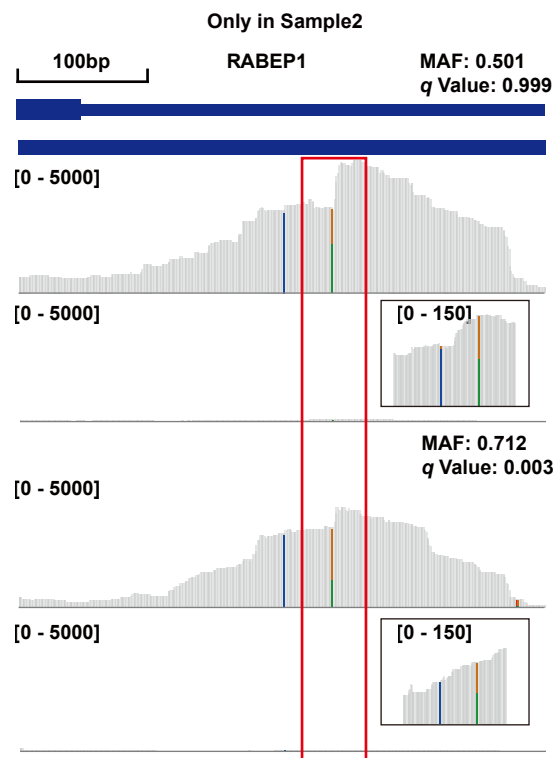**C**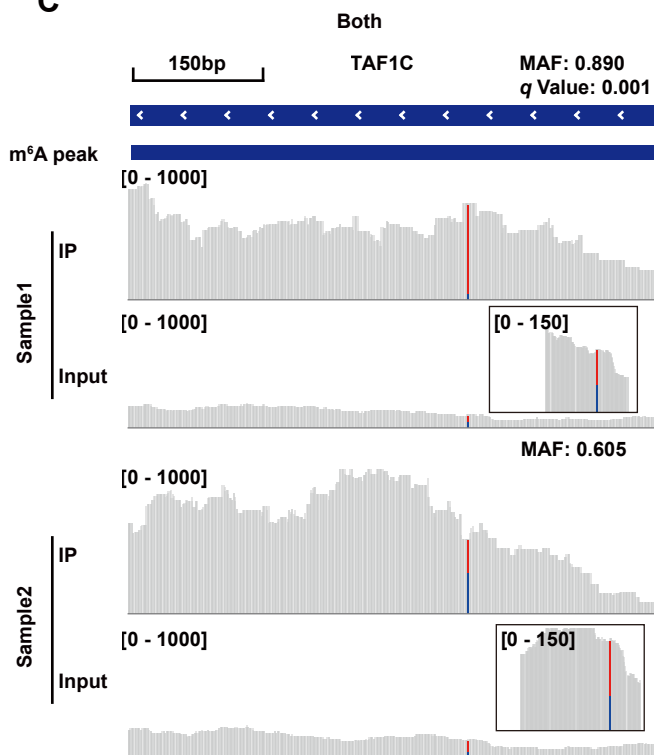**D**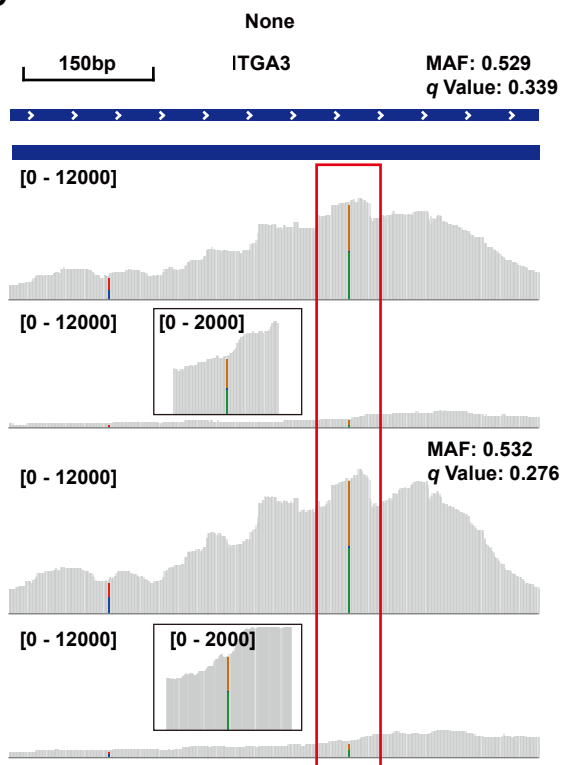

Supplement: giaf040_Supplemental_Files [file giaf040_supplemental_files.zip › Figure S6.pdf]

A

ASE-Gain Genes

ASm<sup>6</sup>A-Gain Genes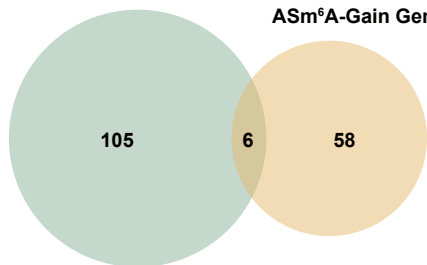

B

ASE-Loss Genes

ASm<sup>6</sup>A-Loss Genes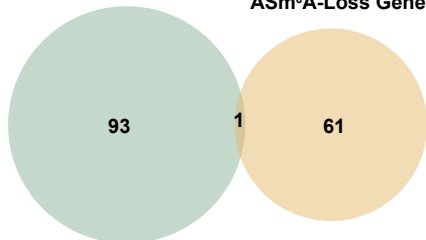

C

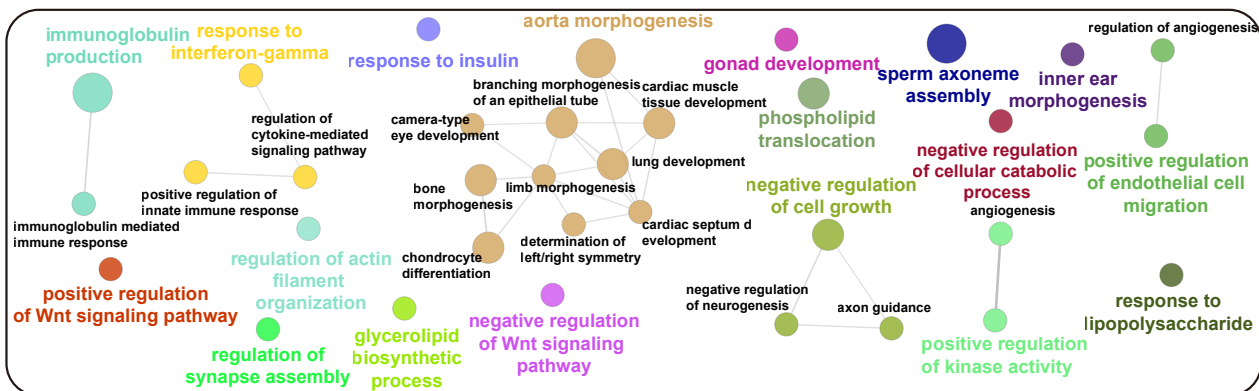

D

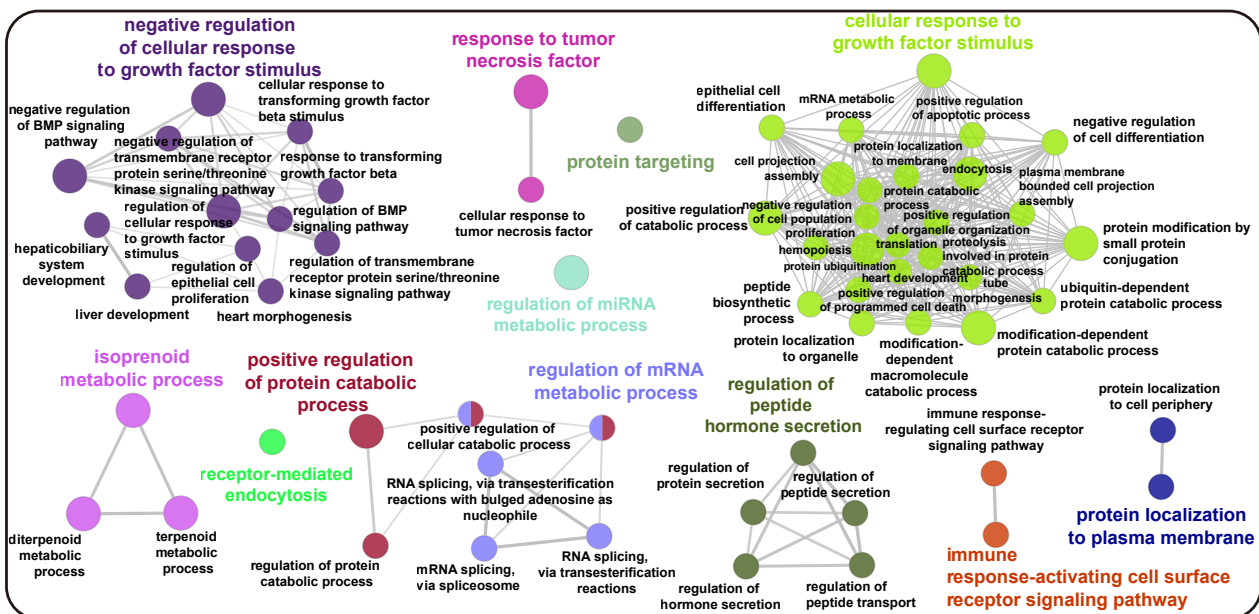

Supplement: giaf040_Supplemental_Files [file giaf040_supplemental_files.zip › Figure S7.pdf]

A

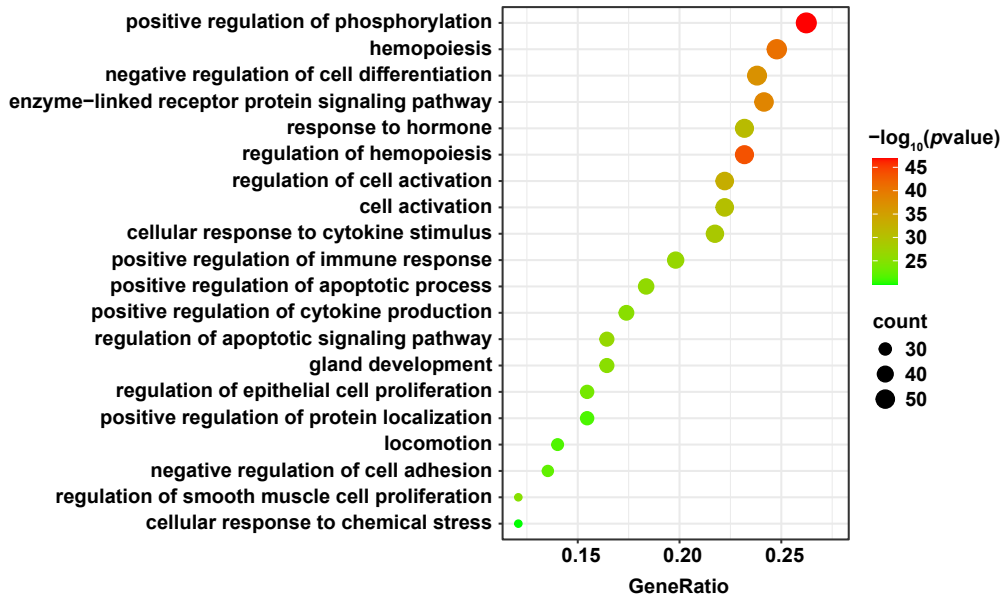

B

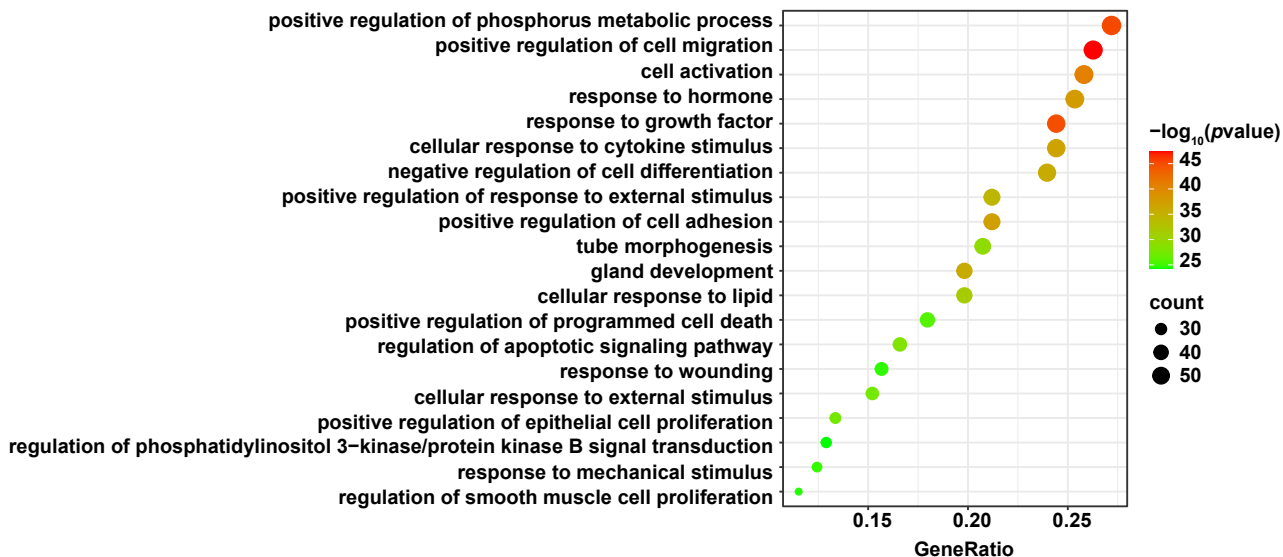

C

Hypergeometric test of Pulmonary Fibrosis-Associated Genes

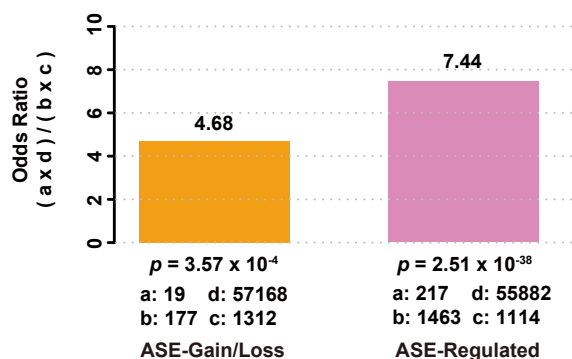

Supplement: giaf040_Supplemental_Files [file giaf040_supplemental_files.zip › Figure S8.pdf]

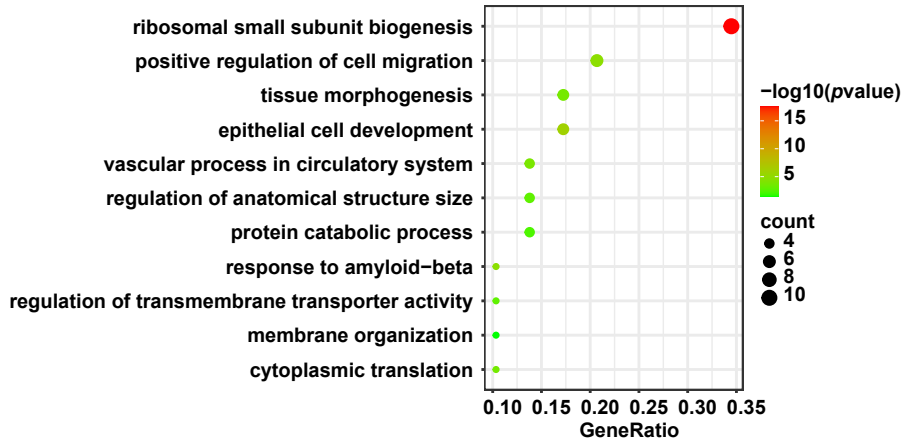

Supplement: giaf040_Supplemental_Files [file giaf040_supplemental_files.zip › Figure S9.pdf]
