# Supplementary material for: M6Allele: a toolkit for detection of allele-specific RNA N6-methyladenosine modifications
Source: Gigascience. 2025 May 19;14:giaf040. doi: 10.1093/gigascience/giaf040 (PMC12087454; doi:10.1093/gigascience/giaf040)
Supplement: giaf040_GIGA-D-24-00340_Revision_1 [file giaf040_giga-d-24-00340_revision_1.pdf]

GigaScience

M6Allele: A toolkit for detection of allele-specific RNA N6-methyladenosine modifications

--Manuscript Draft--

**Manuscript Number:**	GIGA-D-24-00340R1
**Full Title:**	M6Allele: A toolkit for detection of allele-specific RNA N6-methyladenosine modifications
**Article Type:**	Technical Note
**Funding Information:**	National Key Research and Development Program of China (2023YFC2705900) — Dr Yubin Xie<br>National Natural Science Foundation of China (32200542) — Dr Xiaotong Luo<br>National Natural Science Foundation of China (82301233) — Dr Shengyao Zhi<br>Young Elite Scientists Sponsorship Program by Guangzhou Association for Science and Technology (QT-2023-045) — Dr Xiaotong Luo<br>Guangdong Province Excellent Youth Team Project (2024B1515040009) — Dr Jian Ren<br>Discipline training, innovation and quality improvement engineering team project of Guangdong Pharmaceutical University (2024QZ02) — Dr Shengyao Zhi

Abstract:

Background

Allelic gene-specific regulatory events are crucial mechanisms in organisms, pivotal to many fundamental biological processes such as embryonic development and chromosome inactivation. Allelic gene imbalance manifests at both RNA expression and epigenetic levels. Recent research has unveiled allelic-specific regulation of RNA N6-methyladenosine (m6A), emphasizing the need for its precise identification. However, prevailing approaches primarily focus on screening allele-specific genetic variations associated with m6A, not truly identify allelic m6A event. Therefore, the construction of a novel algorithm dedicated to identify allele-specific m6A (ASm6A) signal is still necessary for comprehensively understanding the regulatory mechanism of Asm6A.

Findings

To address this limitation, we have developed a meta-analysis approach employing hierarchical Bayesian models to accurately detect ASm6A events at the peak level from MeRIP-seq data. For user convenience, we introduce a unified analysis pipeline named M6Allele, streamlining the assessment of significant ASm6A across single and paired samples. Applying M6Allele to MeRIP-seq data analysis of pulmonary fibrosis and lung adenocarcinoma reveals enrichment of ASm6A events in key regulatory genes associated with these diseases, suggesting their potential involvement in disease regulation.

Conclusions

Our effort provides a method for precisely identifying ASm6A events at the peak level, elucidates the interplay of m6A with human health and disease genetics, and paves a new visual angle for disease research. The M6Allele software is freely available at https://github.com/RenLabBioinformatics/M6Allele under the MIT license.

Corresponding Author: Xiaotong Luo, Ph.D
Sun Yat-Sen University

Powered by Editorial Manager® and ProduXion Manager® from Aries Systems Corporation

	GuangZhou, CHINA
**Corresponding Author Secondary Information:**	
**Corresponding Author's Institution:**	Sun Yat-Sen University
**Corresponding Author's Secondary Institution:**	
**First Author:**	Yin Zhang
**First Author Secondary Information:**	
**Order of Authors:**	Yin Zhang
	Lin Tang
	Shengyao Zhi
	Bosu Hu
	Zhixiang Zuo
	Jian Ren
	Yubin Xie
	Xiaotong Luo, Ph.D
**Order of Authors Secondary Information:**	
**Response to Reviewers:**	January 6, 2024<br><br>Editorial Office of GigaScience<br><br>Dear Dr. Hans Zauner,<br><br>We are grateful for the prompt review of our manuscript, and helpful comments from two reviewers. According to the reviewers' comments, we have supplemented and improved several aspects of the manuscript. Specifically, we provided a more detailed explanation of the methodology, emphasized the applicability of M6Allele to various types of RNA modifications, and clarified the suitability of different peak-calling tools. In addition, we conducted MeRIP-seq and Sanger sequencing to experimentally verify ASm6A events predicted by M6Allele, evaluated the robustness of the methods through statistical significance tests and variability analyses, assessed the impact of the pseudo-phasing strategy on detection accuracy, and provided additional discussions on the effects of key parameters. Furthermore, we conducted additional comparisons of computational time across different tools. We also replaced and supplemented some IGV examples to present the analysis results more intuitively. Additionally, the software “M6Allele” was approved by SciCrunch.org, with the RRID number SCR_026077, and its bio.tools ID is biotools:m6allele. The workflow has also been registered on WorkflowHub, and the DOI is https://doi.org/10.48546/WORKFLOWHUB.WORKFLOW.1223.1.<br><br>Finally, the newly generated MeRIP-seq data and the raw results of Sanger sequencing have been uploaded to GigaDB for public access (files.gigadb.org/M6Aelle). Additionally, the MeRIP-seq data have also been deposited in the Genome Sequence Archive (GSA) under the accession ID HRA009897.<br><br>The manuscript was revised based on the reviewer’s comments and all the revised texts were marked in red. Please find the point-by-point response below.<br><br>Thanks for your patience and coordination. I look forward to hearing from you.<br><br>Sincerely,<br><br>Xiaotong Luo, PhD<br><br>Point-by-point responses

Powered by Editorial Manager® and ProduXion Manager® from Aries Systems Corporation

Reviewer #1:
1. The method is entitled with "m6A"; however, it seems to me that the developed framework is equally applicable to other RNA modifications that can be profiled with MeRIP-seq technique, such as, m7G and Ac4C. It is possible to include another case study of other modifications. It can be very interesting and substantially expand the coverage of the proposed framework.

Response: Thank you for this valuable comment. Indeed, while focusing on identifying allele-specific modifications (ASM) of m6A in MeRIP-Seq experiments, M6Allele is equally applicable to other types of RNA modifications detected through antibody enrichment followed by sequencing. Since M6Allele primarily analyzes data based on peak information and SNP sites, it can process any modification data with well-defined peaks and SNPs detected via MeRIP-seq or similar approaches.

We have clarified this point in the manuscript, with the following statement added around line 447: “Although M6Allele was originally designed for MeRIP-seq experiments, it is also applicable for peak detection and differential analysis of other RIP-seq data, such as m7G or Ac4C. However, since the peak-calling tools within the M6Allele pipeline are primarily optimized for MeRIP-seq data, users can alternatively upload peak-calling results from other tools to facilitate the analysis of ASM events across various RIP-seq datasets.”

2. MetPeak is recommended as the peak calling method of the framework, which is a little bit surprising to me, as recent studies recommended TRESS and exomePeak2. Are there any particular reasons that MetPeak was recommended, and are other peak calling tools equally suitable?

Response: Thank you for your insightful comment. Indeed, recent tools such as TRESS and exomePeak2 have demonstrated strong performance in peak calling for MeRIP-Seq data, particularly in terms of sensitivity and specificity. In our study, we opted to use MetPeak primarily because it had been successfully applied in our previous studies, RM2Target[1], RMVar[2], and m6AVar[3], making it a natural choice for integration into the pipeline. However, the framework is flexible and also supports other peak-calling tools, including TRESS, exomePeak2, and MACS3.

To address this point, we have added a clarification in the manuscript (line 645), stating: "While MeTPeak was used as the default peak-calling tool in this study, we have tested other peak-calling tools, such as TRESS and exomePeak2, and MACS3, and confirmed that they are also compatible with M6Allele."

Reviewer #2:
1. Authors should validate some called candidate sites, e.g., using MeRIP-seq + Sanger sequencing.

Response: Thank you for your insightful suggestion. To address this point, we conducted experimental validations of candidate ASm6A sites using MeRIP-seq followed by Sanger sequencing on RNA samples derived from the human monocytic THP-1 cell line. Initially, we selected 15 positive and 15 negative candidate ASm6A sites based on M6Allele's predictions. To ensure that the expression levels of the transcripts at the selected sites were adequate for Sanger sequencing, we further examined the total read counts for each site in the Input samples. Sites with fewer than 25 reads were excluded, resulting in 6 positive and 14 negative sites being retained for Sanger sequencing (Additional file 2: Tables S4). Using EditR software to analyze the Sanger sequencing chromatograms, we calculated the proportions of different nucleotides at the selected sites in both the IP and input samples, determining the odds ratio for the major allele[4]. Using an odds ratio greater than 1.2 as the threshold, we classified 9 sites as true positives and 11 as true negatives for ASm6A (Additional file 2: Tables S4). Using these 20 sites, we compared the performance of three ASm6A detection tools. The results indicated that M6Allele achieved a higher AUC compared to the other tools (Fig. 3g), emphasizing its superior accuracy in detecting ASm6A events.

To clarify this point, we have added a detailed explanation in the manuscript (line 265),

Powered by Editorial Manager® and ProduXion Manager® from Aries Systems Corporation

stating:
"To further validate M6Allele's ASm6A detection performance, we conducted experimental validations using MeRIP-seq (GSA-Human: HRA009897) on the human monocytic THP-1 cell line, followed by Sanger sequencing. A total of 20 candidate sites were selected based on predictions from M6Allele (Additional file 2: Table S2, Table S3, Table S4). We utilized EditR software to analyze the Sanger sequencing chromatograms, calculating the ratios of different nucleotides at the selected sites in both the IP and input samples, and determining the odds ratio for the major allele. Using a threshold of greater than 1.2 for the odds ratio, we classified 9 ASm6A sites as true positives and 11 as true negatives. We then compared the performance of three ASm6A detection tools using these 20 sites. The results demonstrated that M6Allele achieved a significantly higher AUC compared to the other two tools (Fig. 3g)."

Additionally, in the Methods section (line 721), we have added the following details to clarify the process of selecting and validating candidate sites: "To validate potential ASm6A events, we initially selected 15 positive and 15 negative candidate sites based on M6Allele's predictions from the THP-1 cell line MeRIP-seq data. Ensure that the expression levels of the transcripts at the selected sites are sufficient for the validation, we filtered the sites by calculating the total read counts in the Input samples and excluded sites with fewer than 25 reads (Additional file 2: Table S4). As a result, a total of 6 positive and 14 negative sites were used for validation. Primers targeting these sites were designed for both Input and IP samples, with detailed primer sequences provided in Additional file 2: Table S3. PCR products were gel-purified and subjected to Sanger sequencing. The sequencing chromatograms were processed using EditR software to calculate the proportions of different nucleotides at the selected sites. Odds ratios for the major allele were calculated to compare nucleotide proportions between IP and input samples, with sites having an odds ratio greater than 1.2 classified as positive ASm6A events."

2. Fig. 2 The authors should conduct a larger number of simulations and report error bars (or confidence intervals).

Response: Thank you for the great comment. To address this point, we conducted the 50 simulation datasets and added error bars to Fig. 2, representing standard deviations, to reflect the variability in the results.

3. Why weren't the performance metrics used in Fig. 2 not used to compare M6Allele to the other two tools for ASm6A?

Response: Thank you for this great suggestion. To address this concern, we compared the performance of M6Allele with two other ASm6A tools using the same metrics as ASE. We specifically analyzed the impact of five factors: read length, library size, FPKM, the number of SNPs per peak, and the number of biological replicates on the performance of the three tools. The results showed that M6Allele outperformed the other tools in terms of F0.5 and F1 scores, which combine precision and recall. Additionally, M6Allele exhibited a lower false discovery rate, highlighting its superior performance. These findings indicate that M6Allele has a clear advantage over the other tools in both ASE and ASM detection tasks.

To clarify this point, we have added a detailed explanation in the manuscript (line 244), stating: "To provide a more comprehensive evaluation, we further compared the detailed performance metrics of different ASm6A detection algorithms (Additional file 1: Fig. S3). The results showed that M6Allele consistently outperformed the other two tools across all categories, combining higher precision and recall while maintaining a lower false discovery rate. Notably, M6Allele exhibited smaller fluctuations and superior stability compared to the other two tools, especially under challenging conditions such as lower library sizes or fewer biological replicates."

4. Fig. 3 Authors should show whether increases in each category produce statistically different changes. For example, does increasing number of biological replicates from 1 to 3 result in a statistically significant drop in average error?

Response: Thank you for your insightful comment. To address this, we conducted statistical analyses to evaluate whether changes in five key variables affect

performance metrics (Fig. 3). The results showed that increasing read length did not have a significant impact on M6Allele's performance, indicating robustness to sequencing read length (Fig. 3a). However, increasing library size, FPKM, the number of SNPs per peak, and the number of biological replicates led to a significant reduction in average error rates (Fig. 3b-e). Notably, increasing the number of biological replicates from 1 to 3 led to a gradual and statistically significant decrease in average error rates, which then stabilized. These findings underscored the ability of M6Allele to effectively utilize high-quality datasets and suggest that experimental designs incorporating sufficient library size and at least three biological replicates can achieve satisfactory performance.

To clarify this point, we have added a detailed explanation in the manuscript (line 215), stating: "The results demonstrated that changes in sequencing read length do not affect the performance of M6Allele (Fig. 3a). However, increases in library size, FPKM, the number of SNPs in a peak, and the number of biological replicates led to a reduction in the average error rate, with particularly pronounced improvements observed for greater library depth and higher gene expression levels (Fig. 3b-e)."

5. Fig. S2 shows very little differences (if any) in performance of a given tool with an increase in any of the categories. For example, increasing number of biological replicates has no effect on performance of any of the tools. What is going on here?

Response: Thank you for your insightful comment. Fig. S2 compares the AUC-ROC of M6Allele and the other two tools under five key variables. While AUC-ROC focuses solely on sensitivity and specificity, merely providing an overview of performance across various thresholds, it does not capture the detailed performance variations that may occur under specific conditions To address this, we calculated additional performance metrics, including precision, recall, F0.5, F1, and FDR (Additional file 1: Fig. S3). These analyses revealed that M6Allele consistently improved with increasing library size, SNP numbers, and biological replicates, outperforming the other two tools in terms of smaller fluctuations and superior stability. For example, with three or more biological replicates, M6Allele exhibited lower error rates and more consistent performance compared to the other tools, even under challenging conditions such as smaller library sizes.

6. Authors should run M6Allele and the two other tools on more real datasets and compare the results.

Response: Thank you for your insightful comment. As mentioned in our response to Comment 1, we performed MeRIP-seq and Sanger sequencing validations using RNA samples from the human monocytic THP-1 cell line. These validations included 20 candidate ASm6A sites derived from M6Allele predictions, along with corresponding results from two other tools. Using Sanger sequencing as the ground truth, we assessed each tool's performance, and M6Allele achieved a significantly higher AUC (0.7778) compared to ASPRIN (0.5455) and the algorithm developed by Cao S et al. (0.5707) (Fig. 3g). These findings highlight M6Allele's ability to outperform other tools in accurately detecting ASm6A modifications.

7. Can the authors evaluate phasing accuracy in simulation studies?

Response: For haplotyping, we referred to the voting-based pseudo-phasing strategy used in MBASED[5], which has demonstrated the feasibility of this approach. To further evaluate the impact of the pseudo-phasing strategy on ASm6A detection, we compared the performance of M6Allele using known phasing information versus the pseudo-phasing strategy. The results (Fig. 3f) showed comparable AUCs, with 0.9216 for known phasing and 0.9039 for the pseudo-phasing strategy. These findings indicate that the pseudo-phasing approach employed by M6Allele is sufficiently accurate to support reliable ASm6A detection.

To clarify this in the manuscript, we have added the following description at line 235: "Furthermore, we also investigated the impact of the pseudo-phasing strategy on the performance of M6Allele. The results indicated that the AUC of M6Allele was 0.9216 with known phasing information, which is comparable to the AUC of 0.9039 obtained with pseudo-phasing strategy (Fig. 3f). This further validates the reliability of the

pseudo-phasing method in ASm6A detection."

8. Computational time should be compared between M6Allele and the other two mentioned approaches using different number of samples.

Response: Thank you for this valuable comment. To address this, we compared the computational time of M6Allele with the two other tools for both ASE and ASM detection tasks. The comparison was conducted using the real datasets mentioned in the manuscript (GSE164151 and GSE198288), which include a total of 12 samples. We evaluated the time cost across five gradients of sample sizes under single-threaded mode for all tools. For ASE detection, the computational speed of M6Allele was comparable to geneiASE, and both were significantly faster than MBASED, with this advantage becoming more pronounced as the sample size increased (Additional file 1: Fig. S4a). For ASM detection, M6Allele achieved intermediate performance, being slower than Cao S et al.'s tool but faster than ASPRIN (Additional file 1: Fig. S4b). Cao S et al.'s tool demonstrated superior speed, particularly for larger sample sizes, which may be partially attributed to differences in the handling of SNP information and statistical modeling strategies. M6Allele performs more comprehensive integration of each SNP information within peak, which could increase computational complexity while improving detection accuracy.

To clarify this in the manuscript, we have added the following description at line 255: "Additionally, we evaluated the computational time of M6Allele in comparison to two other tools for both ASE and ASm6A detection tasks. Using two publicly available MeRIP-seq datasets from GEO database (GSE164151 and GSE198288) with a total of 12 human samples, we tested computational efficiency across five sample size gradients under single-threaded mode. The results showed that M6Allele exhibited comparable speed to geneiASE for ASE detection and intermediate performance for ASm6A detection, being slower than Cao S et al.'s tool but faster than ASPRIN (Additional file 1: Fig. S4). This difference in speed may be attributed to M6Allele's more comprehensive integration of SNP information within peaks, which increases computational complexity while ensuring higher detection accuracy."

9. The total number of reads in IP and input for Fig. S3 A and B are very similar to me. Seems like these should not be called as m6A peaks to begin with.

Response: Thank you for pointing out this observation. We agree that the peaks shown in Fig. S5A and S5B (previously S3A and S3B) may not be ideal examples of m6A peaks due to the similar number of reads in IP and input samples. This could be attributed to certain aspects of the peak-calling algorithm, which may occasionally lead to the identification of peaks with less distinct enrichment. To address this, we have replaced these examples with more representative peaks that exhibit a clearer enrichment in IP over input.

10. Authors should show IGV examples of differential allele-specific m6A.

Response: Thank you for your suggestion. To address this, we have added IGV examples illustrating differential allele-specific m6A modifications. These examples have been included in the revised supplementary materials as Fig. S6.

Additionally, we have incorporated the following description at line 288 in the manuscript: "To illustrate these four ASm6A categories, we provided IGV visualizations of randomly selected examples for each category (Additional file 1: Fig. S6). The observed haplotype distributions in IP and Input samples were consistent with M6Allele's detection results for differential ASm6A events."

Reference

1.Bao X, Zhang Y, Li H, Teng Y, Ma L, Chen Z, Luo X, Zheng J, Zhao A, Ren J, Zuo Z. RM2Target: a comprehensive database for targets of writers, erasers and readers of RNA modifications. Nucleic Acids Res. 2023; 51:D269-D279.
2.Luo X, Li H, Liang J, Zhao Q, Xie Y, Ren J, Zuo Z. RMVar: an updated database of functional variants involved in RNA modifications. Nucleic Acids Research. 2021; 49:D1405-D1412.

	3.Zheng Y, Nie P, Peng D, He Z, Liu M, Xie Y, Miao Y, Zuo Z, Ren J. m6AVar: a database of functional variants involved in m6A modification. Nucleic Acids Res. 2018; 46:D139-D145.<br>4.Kluesner MG, Nedveck DA, Lahr WS, Garbe JR, Abrahante JE, Webber BR, Moriarity BS. EditR: A Method to Quantify Base Editing from Sanger Sequencing. CRISPR J. 2018; 1:239-250.<br>5.Mayba O, Gilbert HN, Liu J, Haverty PM, Jhunjhunwala S, Jiang Z, Watanabe C, Zhang Z. MBASED: allele-specific expression detection in cancer tissues and cell lines. Genome Biol. 2014; 15:405.
**Additional Information:**	
**Question**	**Response**
Are you submitting this manuscript to a special series or article collection?	No
**Experimental design and statistics**<br><br>Full details of the experimental design and statistical methods used should be given in the Methods section, as detailed in our Minimum Standards Reporting Checklist. Information essential to interpreting the data presented should be made available in the figure legends.<br><br>Have you included all the information requested in your manuscript?	Yes
**Resources**<br><br>A description of all resources used, including antibodies, cell lines, animals and software tools, with enough information to allow them to be uniquely identified, should be included in the Methods section. Authors are strongly encouraged to cite Research Resource Identifiers (RRIDs) for antibodies, model organisms and tools, where possible.<br><br>Have you included the information requested as detailed in our Minimum Standards Reporting Checklist?	Yes
**Availability of data and materials**<br><br>All datasets and code on which the conclusions of the paper rely must be	Yes

either included in your submission or deposited in publicly available repositories (where available and ethically appropriate), referencing such data using a unique identifier in the references and in the "Availability of Data and Materials" section of your manuscript.<br><br>Have you have met the above requirement as detailed in our Minimum Standards Reporting Checklist?	

Powered by Editorial Manager® and ProduXion Manager® from Aries Systems Corporation

 Click here to access/download;Manuscript;M6Allele-submit-to-GigaScience-Manuscript_revise.docx

M6Allele: A toolkit for detection of allele-specific RNA N^6-methyladenosine modifications

Yin Zhang[1#], Lin Tang[1#], Shengyao Zhi[2#], Bosu Hu[1], Zhixiang Zuo[3], Jian Ren[1], Yubin Xie[4*], Xiaotong Luo[1,5*]

[#]Yin Zhang, Lin Tang, and Shengyao Zhi contributed equally to this work.

[1] Innovation Center of the Sixth Affiliated hospital, School of Life Sciences, Sun Yat-sen University, Guangzhou, 510060, China

[2] Guangdong Provincial Key Laboratory of Pharmaceutical Bioactive Substances, School of Biosciences and Biopharmaceutics，Guangdong Pharmaceutical University, Guangzhou, 510006, China

[3] State Key Laboratory of Oncology in South China, Cancer Center, Collaborative Innovation Center for Cancer Medicine, Sun Yat-sen University, Guangzhou, 510060, China

[4] Institute of Precision Medicine, The First Affiliated Hospital, Sun Yat-sen University, Guangzhou, 510060, China.

[5] Guangdong Institute of Gastroenterology, Biomedical Innovation Center, The Sixth Affiliated Hospital, Sun Yat-sen University, Guangzhou, 510060, China

*Correspondence:

luoxt36@mail.sysu.edu.cn; xieyb6@mail.sysu.edu.cn

Abstract

Background

Allelic gene-specific regulatory events are crucial mechanisms in organisms, pivotal to many fundamental biological processes such as embryonic development and chromosome inactivation. Allelic gene imbalance manifests at both RNA expression and epigenetic levels. Recent research has unveiled allelic-specific regulation of RNA N^6-methyladenosine (m^6A), emphasizing the need for its precise identification. However, prevailing approaches primarily focus on screening allele-specific genetic variations associated with m^6A, not truly identify allelic m^6A event. Therefore, the construction of a novel algorithm dedicated to identify allele-specific m^6A (ASm^6A) signal is still necessary for comprehensively understanding the regulatory mechanism of Asm^6A.

Findings

To address this limitation, we have developed a meta-analysis approach employing hierarchical Bayesian models to accurately detect ASm^6A events at the peak level from MeRIP-seq data. For user convenience, we introduce a unified analysis pipeline named M6Allele, streamlining the assessment of significant ASm^6A across single and paired samples. Applying M6Allele to MeRIP-seq data analysis of pulmonary fibrosis and lung adenocarcinoma reveals enrichment of ASm^6A events in key regulatory genes associated with these diseases, suggesting their potential involvement in disease regulation.

Conclusions

Our effort provides a method for precisely identifying ASm^{6}A events at the peak level, elucidates the interplay of m^{6}A with human health and disease genetics, and paves a new visual angle for disease research. The M6Allele software is freely available at https://github.com/RenLabBioinformatics/M6Allele under the MIT license.

Keywords

Allele-specific, RNA N^{6}-methyladenosine (m^{6}A), Hierarchical Bayesian model, Meta-analysis

Introduction

In a non-haploid genome, the transcriptional activity at different gene alleles can vary significantly [1]. Allele-specific effects are crucial in various cellular activities, particularly genomic imprinting[2], chromosome inactivation[3], and the regulation of gene expression in particular spatiotemporal circumstances[4]. Mechanisms such as random mono-allelic expression[5, 6], allele sequence-specific expression, and parental-specific (imprinted) expression[7, 8] have been shown to result in the expression of only one allele for many genes. Allele-specific gene expression (ASE) can impact disease traits, including biological developmental abnormalities[9], cardiovascular and cerebrovascular dysfunctions[10], progressive genetic disorders[11], and even cancers[12, 13]. In addition to ASE, allelic imbalance is evident in epigenetic regulation. Extensive research has focused on allele-specific DNA methylation as a factor that controls allele-specific expression[14, 15]. Notably, approximately 10% of human genes are regulated by allele-specific DNA

methylation[16]. While these studies primarily focused on DNA-level modifications that influence allele-specific regulation, RNA-level modifications have received less attention. Similar to DNA methylation, RNA methylation is a common and reversible epigenetic modification found in RNA nucleotides. Among all the RNA methylation types, N^6-methyladenosine (m^{6}A) is the most common modification in eukaryotic messenger RNAs (mRNAs), accounting for over 80% of known RNA modifications[17]. m^{6}A is also extensively present in microRNAs (miRNAs)[18], long non-coding RNAs (lncRNAs)[19], and circular RNAs (circRNAs)[20]. m^{6}A is widely involved in a variety of important cell processes, including embryonic development[21], apoptosis[22] and sperm development[23] as well as in a large number of malignant diseases, such as tumors and obesity[24, 25]. Therefore, m^{6}A is a key factor for understanding disease pathogenesis and developing new therapies.

Recent studies have revealed that allelic regulations were also existed in m^{6}A modifications[26]. For example, *Ane Olazagoitia-Garmendia* et al. have shown that ASm^{6}A in lncRNAs, like LOC339803, affects protein binding and chromatin localization, and that an SNP in the 5'UTR of XPO1 associated with coeliac disease, which is close to three m^{6}A consensus motifs (GGACT), exhibits higher m^{6}A methylation, leading to increased XPO1 protein levels and activation of nuclear factor kappa B (NFkB), contributing to inflammation[27, 28]. To identify the transcriptome-wide ASm^{6}A, *Cao S* et al. recently applied Fisher's exact test to detect ASm^{6}A at the SNP level in MeRIP-seq data. They identified 12,056 allele-specific SNPs located in m^{6}A peaks from human tissues and found that many of them are associated with risk variants in common diseases[29]. In addition,

Xing Yi et al. developed ASPRIN[30] (Allele-Specific Protein-RNA Interaction) to identify genetic variations that alter RBP-RNA interactions by jointly analyzing CLIP-seq and RNA-seq data, which can theoretically also be applied to analyze variant sites associated with ASm^{6}A on MeRIP-seq. However, both of these methods only estimated allele-specific imbalance of m^{6}A peaks at the SNP level, actually not truly identified allelic m^{6}A events, making it challenging to interpret the underlying mechanisms of ASm^{6}A in different biological processes. Since MeRIP-seq provides modification peaks of approximately 200nt, actual data demonstrates that a significant number of m^{6}A peaks possess multiple detectable heterozygous SNPs. This underscores the importance of having a framework for integrating expression information across individual sites in a peak region to accurately assess allele-specific imbalance of m^{6}A. However, there is currently no standard or robust method for summarizing information across SNPs into a single measure of ASm^{6}A for the entire peak.

To overcome these difficulties, we developed a new ASm^{6}A detection method, named M6Allele, which employs a hierarchical Bayesian model to assess ASm^{6}A by integrating information across individual heterozygous SNPs within a peak, even without any prior knowledge of haplotype phasing[31]. Our approach demonstrates higher precision and fewer false positives compared to previous methods using Fisher's exact test. For users' convenience, we have built a comprehensive toolkit for the one-stop analysis of ASm^{6}A from MeRIP-seq data (https://renlab.oss-cn-shenzhen.aliyuncs.com/M6Allele/m6allelepipe.tar.gz). We applied M6Allele to a panel of human pulmonary fibrosis tissues and paired tumor-normal lung tissue samples. The

results indicated enrichment of disease-specific ASm^{6}A modifications in pathogenic genes, suggesting a potential role for ASm^{6}A in disease regulation. Our study introduced a novel meta-analytic approach that enables the precise and sensitive analysis of the dynamic network of ASm^{6}A at the peak level. This method facilitates the identification of specific m^{6}A modifications occurring at the allele level, as well as the comprehension of their association with human health and disease.

Results

M6Allele: meta-analysis based detection of allele-specific m^{6}A modifications

In our study, we introduced M6Allele, an algorithm designed for identifying ASm^{6}As in MeRIP-seq data (Fig. 1a). Initially, high-confidence heterozygous SNVs were identified through variant calling, with rigorous filtering applied to mitigate transcription and mapping biases[32]. Variants were retained if they were absent in RNA editing sites (RADAR database) [33] but present in the dbSNP database. Subsequently, we calculated the read counts of alleles from m^{6}A IP and Input sequencing data, followed by a hierarchical Bayesian model to evaluate the modification difference between the two alleles at individual SNPs within a modification unit. For M6Allele, we chose peaks as units, which can be obtained through peak calling tools commonly used in MeRIP-seq data analysis. Therefore, we only considered SNPs located in the peak regions.

M6Allele requires prior knowledge of gene haplotype specifications, which are likely unknown for the MeRIP-seq dataset. To determine the allelic origin specificity of reads, we adopted MBASED's strategy[34] and introduced a pseudo-phasing approach for SNPs.

Specifically, for each retained SNP, we counted the frequencies of different base types in the m^{6}A Input sample separately. The two base types with the highest frequencies are assigned as the "major" and "minor" haplotypes, respectively. To precisely detect allelic imbalance within peaks, we quantified it as the odds ratio of the major allele relative to the minor allele in the m^{6}A IP sample. The detection in ASm^{6}A then became the identification of peaks with an odds ratio significantly >1.

To accurately evaluate the allelic imbalance of a m^{6}A peak, we utilized a Random Effects Model (REM)[35] to integrate the odd ratios of all SNPs within the peak. Essentially, we considered the regression coefficients of the fixed effects model (in our case, ASm^{6}A) as random variables, assuming all coefficients follow the same normal distribution. By constructing a hierarchical Bayesian model, we estimated the mean of this normal distribution, which served as the ASm^{6}A odds ratio for the entire peak. Similarly, to remove the influence of ASE on assessing ASm^{6}A events, we constructed a hierarchical Bayesian model for ASE using m^{6}A Input samples at the gene level. The odds ratio of ASE obtained served as the background odds ratio for the ASm^{6}A model.

Because of the pseudo-phasing strategy used by M6Allele to infer gene haplotypes, the statistical significance of ASm^{6}A may lead to anti-conservative nominal P-values[35]. To effectively address this issue, we simulated MeRIP-seq data using SNP loci from the Thousand Genomes Project (1000 Genomes) (http://ftp.1000genomes.ebi.ac.uk/vol1/ftp/phase3/data) and dbSNP[36] databases to mimic the absence of allele-specific events. We introduced the Generalized Pareto Distribution (GPD)[37] for fitting the deviation of allelic odds ratio under pseudo-phasing,

to adjust the statistical significance level. M6Allele converts the odds ratio of each peak into the frequency of the major allele (MAF) and provides its corresponding p-value. By adjusting the p-values using the Benjamini-Hochberg (BH) method[38], we obtain Q-values. A peak with a Q-value below 0.05 is considered a significant allelic m^{6}A imbalance event.

Additionally, M6Allele includes a paired-sample analysis module for detecting differential ASm^{6}A between paired samples from the same individual. Given that true haplotypes are unknown, maintaining the consistency of haplotypes across paired samples involves designating one as the source of pseudo-phasing. For instance, in a tumor versus normal comparison, we designate the normal sample as the control group and classify haplotypes into 'major' and 'minor' alleles based on read counts obtained from the Input sample. Differences between m^{6}A odds ratio at individual SNP in the two samples are used as measures of sample-specific ASm^{6}A. SNP-level scores are combined into a peak-level score using meta-analysis and a hierarchical Bayesian model, which is analogous to the single sample approach. This composite odds ratio provides an estimate of the peak-level odds ratio difference between samples.

The details of M6Allele are provided in Methods and Supplementary Methods. Our algorithm is implemented in Java, and the corresponding JAR file has been built. For users' convenience, we've developed an integrated pipeline for ASm^{6}A analysis using Docker (https://www.docker.com/)(Fig. 1b).

Robust allele-specific m^{6}A detection by M6Allele

Because of the absence of gold-standard MeRIP-seq data featuring allele-specific events,

we aimed to evaluate the performance of M6Allele in the absence of phasing information using simulated MeRIP-seq data. The simulation process detailed in the Additional file 1: Fig. S1, draws inspiration from the methods of Polyester[39] for simulating RNA-seq.

As accurate ASE results are essential for M6Allele to assess ASm^{6}A effectively, we initially evaluated the ASE detection performance of M6Allele using simulated RNA-seq data. During the ASE simulation, 50% of transcripts were randomly selected to represent positive ASE events. For these transcripts, the MAF was uniformly sampled from [0.6, 0.9], while the rest had an MAF of 0.5. Additionally, to assess M6Allele's robustness in identifying significant ASE events, we simulated RNA-seq data with different sequencing read lengths (75, 100, 150, and 300nt) 50 times each. Then, we applied M6Allele's ASE detection method to each simulated dataset, considering genes with a Q-value $\leqslant$ 0.05 as significant ASE events. Among the current ASE detection tools, GeneiASE[40] and MBASED[34] can only utilize RNA-seq data to identify ASE events. Consequently, we conducted a performance comparison of M6Allele with these tools (Fig. 2a-e). We observed that the overall precision of M6Allele remains robust across various simulated sequencing read lengths, showing minimal impact (Fig. 2a). However, recall increases with longer read lengths (Fig. 2b). We maintained overall false discovery rate (FDR) at a nominal level of 5%, affirming the effectiveness of p-value adjustment (Fig. 2c). By integrating precision and recall results, we calculated the F0.5 and F1 scores[41] to comprehensively assess the performance of M6Allele in ASE identification (Fig. 2d,e). Comparing M6Allele to two other ASE detection tools reveals its consistently superior performance (Additional file 2: Table S1), indicating its precision in ASE detection is

suitable for downstream analysis. To further validate M6Allele's ASE detection performance on real data, we used M6Allele to identify ASE in the RNA-seq data GSM4998283. Among the results, we chose a gene (RMRP) with significant ASE and one (H1-3) without significant ASE. Visualization with the IGV tool (Fig. 2f) showed their haplotypes distributions, confirming M6Allele's accurate identification of ASE events, consistent with reality.

We subsequently assessed the detection performance of ASm^{6}A by M6Allele using simulated data. To ensure the simulated dataset accurately reflected the genuine peak lengths and distribution of m^{6}A modifications, we incorporated ASm^{6}A events into the simulation by leveraging m^{6}A peaks and sites from GSM1828594. Moreover, for a comprehensive analysis of M6Allele's performance, we categorized all test peaks within the samples based on five pertinent variables, including read lengths, library size, FPKM of gene expression, the number of SNPs in a peak, and the number of biological replicates. In each category, 50% of the peaks was randomly designated as allele-specific, i.e. true positives for ASm^{6}A (MAF > 0.6), while the rest were labeled as true negatives for ASm^{6}A (MAF = 0.5). For robust evaluation, each simulated dataset was repeatedly analyzed 50 times. The results demonstrated that changes in sequencing read length do not affect the performance of M6Allele (Fig. 3a). However, increases in library size, FPKM, the number of SNPs in a peak, and the number of biological replicates led to a reduction in the average error rate, with particularly pronounced improvements observed for greater library depth and higher gene expression levels (Fig. 3b-e). Despite these variations, in simulated data tests, M6Allele consistently maintained an error rate below 10%, even in small libraries or

for genes with low expression levels. This underscores the robustness of the M6Allele model and demonstrates its applicability to sequencing data across diverse experimental conditions.

Furthermore, we compared the performance of M6Allele with two additional tools capable of detecting ASm^{6}A events, ASPRIN[30] and the algorithm developed by *Cao S* et al[29]. We followed the tutorials provided by the two tools, sticking to their default parameter settings. As these tools can only obtain individual SNP sites associated with ASm^{6}A, we aligned the SNPs with m^{6}A peaks. If any SNP within a peak was identified as having ASm^{6}A modification by ASPRIN or *Cao S* et al.'s algorithm, the peak was classified as ASm^{6}A modified, resulting in a positive outcome; otherwise, it was considered non-ASm^{6}A. According to the ASm^{6}A detection results from various algorithms, we calculated the area under the ROC curve (AUC) for each category of simulated peaks. The results indicated that, across diverse settings of the simulated data, M6Allele consistently exhibits a significantly higher average AUC compared to the other two algorithms (Fig. 3f; Additional file 1: Fig. S2). Furthermore, we also investigated the impact of the pseudo-phasing strategy on the performance of M6Allele. The results indicated that the AUC of M6Allele was 0.9216 with known phasing information, which is comparable to the AUC of 0.9039 obtained with pseudo-phasing strategy (Fig. 3f). This further validates the reliability of the pseudo-phasing method in ASm6A detection. Since the other two methods identify SNP sites related to ASm^{6}A, they were more susceptible to the influence of different sequencing conditions. As the observed data on peaks increased, such as the number of covered SNPs or biological replicates, their performance improved significantly. It suggested that

relying solely on individual SNPs to identify ASm^{6}A may struggle to avoid errors caused by the noise of sequencing data. To provide a more comprehensive evaluation, we further compared the detailed performance metrics of different ASm^{6}A detection algorithms (Additional file 1: Fig. S3). The results showed that M6Allele consistently outperformed the other two tools across all categories, combining higher precision and recall while maintaining a lower false discovery rate. Notably, M6Allele exhibited smaller fluctuations and superior stability compared to the other two tools, especially under challenging conditions such as lower library sizes or fewer biological replicates. This underscores the robustness of M6Allele, particularly compared to *Cao S* et al.'s algorithm, which showed significant performance improvement with increasing gene expression, potentially indicating its relatively higher restriction on the number of reads and lower sensitivity in identifying ASm^{6}A signals in low-expressed genes.

Additionally, we evaluated the computational time of M6Allele in comparison to two other tools for both ASE and ASm^{6}A detection tasks. Using two publicly available MeRIP-seq datasets from GEO database (GSE164151 and GSE198288) with a total of 12 human samples, we tested computational efficiency across five sample size gradients under single-threaded mode. The results showed that M6Allele exhibited comparable speed to geneiASE for ASE detection and intermediate performance for ASm^{6}A detection, being slower than *Cao S* et al.'s tool but faster than ASPRIN (Additional file 1: Fig. S4). This difference in speed may be attributed to M6Allele's more comprehensive integration of SNP information within peaks, which increases computational complexity while ensuring higher detection accuracy.

To further validate M6Allele's ASm^{6}A detection performance, we conducted experimental validations using MeRIP-seq (GSA-Human: HRA009897) on the human monocytic THP-1 cell line, followed by Sanger sequencing. A total of 20 candidate sites were selected based on predictions from M6Allele (Additional file 2: Table S2, Table S3, Table S4). We utilized EditR software to analyze the Sanger sequencing chromatograms, calculating the ratios of different nucleotides at the selected sites in both the IP and input samples, and determining the odds ratio for the major allele. Using a threshold of greater than 1.2 for the odds ratio, we classified 9 ASm^{6}A sites as true positives and 11 as true negatives. Using these 20 sites, we compared the performance of three ASm^{6}A detection tools. The results demonstrated that M6Allele achieved a significantly higher AUC compared to the other two tools (Fig. 3g). Additionally, we analyzed the IP and Input samples from GSE164151 (GSM4998285 and GSM4998284). From the results, we randomly selected three peaks with significant ASm^{6}A and three peaks showing no significant ASm^{6}A for visualization using IGV (Fig. 3h and Additional file 1: Fig. S5). Their haplotype distributions in IP and Input samples confirmed M6Allele's precise identification of ASm^{6}A events, aligning with actual observations.

Similarly, simulations were performed in the paired-sample setting (Supplementary Methods). To evaluate M6Allele's accuracy of detecting sample-specific ASm^{6}A events in the paired-sample analysis, MeRIP-seq data for paired-samples were generated using identical genotypic and m^{6}A peaks. Then, 956 peaks were randomly classified into four ASm^{6}A categories: absent in both samples, present only in Sample 1, present only in Sample 2, and present in both samples. Through paired-sample analysis using M6Allele

and comparing the results with the peak assignments (Fig. 3i), precise identification of sample-specific ASm^{6}A events was observed, achieving an overall accuracy of 89.9%. To illustrate these four ASm^{6}A categories, we provided IGV visualizations of randomly selected examples for each category (Additional file 1: Fig. S6). The observed haplotype distributions in IP and Input samples were consistent with M6Allele's detection results for differential ASm^{6}A events.

ASm^{6}A modifications are closely associated with pulmonary fibrosis

The impact of ASm^{6}A modification on human diseases is our focal point. However, only a few studies report an association between ASm^{6}A and diseases. Previous studies demonstrated that pulmonary fibrosis is a typical disease regulated by m^{6}A modification. To further investigate the impact of ASm^{6}A modification on pulmonary fibrosis, we utilized the M6Allele to analyze the distribution of ASE and ASm^{6}A events in patients with pulmonary fibrosis (Additional file 2: Table S5, Table S6). We identified widespread ASE and ASm^{6}A modifications across 22 pairs of autosomal chromosomes in patients with pulmonary fibrosis (Fig. 4a, b). Compared to normal human tissue, we found 111 genes exhibiting significant ASE exclusively in all pulmonary fibrosis patient tissues (referred to as ASE-Gain), along with 94 genes showing significant ASE only in normal tissue (referred to as ASE-Loss) at the whole-genome level (Additional file 1: Fig. S7a, b). Similarly, we detected 64 specific ASm^{6}A-modified genes (ASm^{6}A-Gain) and 62 genes with ASm^{6}A-Loss in pulmonary fibrosis patient tissues. We found very few genes shared between ASE and ASm^{6}A, with only six genes showing a gain of both ASE and ASm^{6}A, and just one

gene showing a loss of both ASE and ASm^{6}A (Additional file 1: Fig. S7a, b). It suggested that ASm^{6}A may exert its regulatory function through alternative mechanisms instead of only impact allelic gene expression. Next, we conducted pathway enrichment analysis on genes associated with ASE and ASm^{6}A events with a FDR < 0.05, utilizing the "GO Biological Processes" dataset from the Metascape database[42] (Fig. 4c, d and Additional file 1: Fig. S7c, d). In the patient tissues, genes with ASm^{6}A-Gain were significantly enriched in immune response, complement activation classical pathway, Rho protein signaling, and other functional pathways closely related to human pulmonary fibrosis disease (Fig. 4c). Meanwhile, genes exhibiting ASE-Gain in pulmonary fibrosis were enriched in aorta morphogenesis, response to interferon-gamma, negative regulation of cell growth, and other pathways related to lung vasculature, cell, and immunity (Additional file 1: Fig. S7c). ASm^{6}A-Loss genes were visibly enriched in pathways associated with epithelial cell differentiation, MAP kinase activation, changes in cell morphology, immune activation response, and platelet-derived growth factors associated with pulmonary fibrosis diseases (Fig. 4d). Genes with ASE-Loss in pulmonary fibrosis played crucial roles in growth factor and metabolism-related pathways (Additional file 1: Fig. S7d). These results suggested that ASE and ASm^{6}A events may collectively influence the development of pulmonary fibrosis through interconnected pathways.

To further elucidate the regulatory relationship between ASm^{6}A and pulmonary fibrosis, we compared known pulmonary fibrosis-related genes ($Score_{GDA} \geq 0.3$) from the DisGeNET database[43] with ASm^{6}A-Gain and ASm^{6}A-Loss genes in pulmonary fibrosis patients. Initially, we conducted a hypergeometric test to analyze the relationship between

ASm^6A-Gain and ASm^6A-Loss genes and known pulmonary fibrosis genes, utilizing all annotated genes in the GTF file of hg38 as sample population, totaling 58,676 genes. The result ($p < 5 \times 10^{-7}$) revealed a significant enrichment of ASm^6A-modified genes within the pulmonary fibrosis gene set (the blue bar in Fig. 4e). To delve deeper into the regulation of pulmonary fibrosis-associated genes by ASm^6A modification, we identified genes interacting with ASm^6A-modified genes with confidence of 0.9 from the STRING database[44] and determined their overlap with pulmonary fibrosis-related genes. In ASm^6A-modified genes and their interactors, referred to as ASm^6A-regulated genes, hypergeometric testing unveiled a significant enrichment of pulmonary fibrosis-related genes (the green bar in Fig. 4f). Functional pathway analysis of this gene overlaps highlighted significant enrichment in pathways crucial to pulmonary fibrosis pathogenesis, notably positive regulation of phosphorylation[45] negative regulation of cell differentiation[46] and positive regulation of immune response[46] (Additional file 1: Fig. S8a). Additionally, we conducted a similar analysis on ASE genes (Additional file 1: Fig. S8b). With the overlapping genes showing enrichment in pathways such as positive regulation of cell migration[47], response to growth factor[48], and negative regulation of cell differentiation[49]. The hypergeometric test between ASE genes and pulmonary fibrosis-related genes revealed a significant enrichment of ASE genes among pulmonary fibrosis-related genes (the orange bar in Additional file 1: Fig. S8c). Meanwhile, pulmonary fibrosis-related genes were also significantly enriched among ASE genes and their interactors (the pink bar in Additional file 1: Fig. S8c). The above findings suggest that genes with allele-specific events identified by M6Allele may interact with known pulmonary

fibrosis-related genes, regulate related pathways, and thus influence the progression of pulmonary fibrosis diseases. M6Allele can unearth ASm^{6}A-modified genes closely related to diseases from the MeRIP-seq data, providing a new direction for research on the pathogenesis and treatment of human diseases.

M6Allele reveals the lung adenocarcinoma-associated ASm^{6}A with high heterogeneity

It has previously been reported that m^{6}A modification can regulate the occurrence and development of cancers[50], particularly in lung adenocarcinoma, a kind of malignant tumor with the highest mortality rate[51, 52]. Notably, there have been no reports on whether ASm^{6}A modification regulates the progression of malignant tumors. To further explore the impact of ASm^{6}A modification on lung adenocarcinoma, we used the M6Allele to identify ASm^{6}A events in lung adenocarcinoma patients[53] (Additional file 2: Table S7). In cancer research, we typically emphasize intergroup differences between tumors and adjacent tissues unaffected by individual genetic information, such as sample-specific ASm^{6}A (ssASm^{6}A) events. As ASm^{6}A events achieved from unpaired-sample analysis of tumor samples often include many events unrelated to the disease, such as the patient's inherited ASm^{6}A events, filtering out these false positives is crucial for identifying disease-relevant ASm^{6}A modifications. Therefore, we compared two strategies, the unpaired-sample and paired-sample analysis, to exclude false positive ssASm^{6}A events. The results of the single-sample analysis showed that the tumor samples from three patients respectively had 382, 339, and 651 peaks with ASm^{6}A, while in the normal samples, there

were 446, 536, and 451 peaks with ASm^{6}A (Fig. 5a). Through paired-sample analysis, we found that only 17% to 49% of the ASm^{6}A events identified in unpaired-sample analysis were recognized as single-sample ASm^{6}A signals (Fig. 5b). The remaining ASm^{6}A signals were present in both tumor and normal samples, suggesting these events may be inherent epigenetic regulatory events in patients unrelated to the tumor. These results illustrate that paired-sample analysis can effectively screen for ssASm^{6}A modifications and identify significant differences in ASm^{6}A events between samples. Therefore, in downstream analysis, we focused solely on single-sample ASm^{6}A events.

To examine the uniformity of ssASm^{6}A sites among different patient samples, we combined the analysis results to create a Venn diagram. The results reveal that, among the 422, 247, and 158 tumor ssASm^{6}A Gain genes identified in the three patient samples, only 6 genes were shared (Fig. 5c). Similarly, there were only 9 shared tumor ssASm^{6}A Loss genes in the three patient samples, while the identified genes were 421, 367, and 321, respectively (Fig. 5d). These findings indicate that the tumor ssASm^{6}A modified genes identified in different patients with lung adenocarcinoma differ significantly, and the Gain and Loss of ASm^{6}A also vary notably across different patient samples. Moreover, the proportion of identified ASm^{6}A-modified genes existing alone in a single sample accounted for as high as 79.95% (674 in 843), 73.78% (453 in 614), and 75.16% (360 in 479), respectively. These results highlight the highly heterogeneous and complex nature of lung adenocarcinoma-associated ASm^{6}A modifications.

To prove the effectiveness of the algorithm, we annotated the 15 ssASm^{6}A genes shared among the three patient samples through the literature review (Additional file 2:

Table S8). Among these, 5 genes were reported to be directly associated with lung adenocarcinoma, 4 genes were associated with lung cancer but not specifically with lung adenocarcinoma, and 6 genes were not reported to be related to lung cancer but were found to be associated with other types of cancer. Furthermore, our hypergeometric testing revealed significant enrichment of these 15 genes among lung cancer-related genes from DisGeNet ($Score_{GDA} \geqslant 0.3$, the red bar in Fig. 4e). These findings demonstrate the algorithm's effectiveness in identifying significant genes related to cancer. In addition, genes associated with lung adenocarcinoma ($Score_{GDA} \geq 0.3$ in DisGenet) were significantly enriched among ssASm^{6}A-regulated genes including ssASm^{6}A-modified genes and their interactors (confidence ≥ 0.9 in STRING) (the purple bar in Fig.4f). It suggests that ssASm^{6}A may directly or indirectly regulate the occurrence and development of lung cancer by modifying disease-related genes and interacting proteins. We conducted functional pathway analysis on the overlapping genes, revealing significant enrichment in pathways related to lung cancer, such as positive regulation of cell migration[54], epithelial cell development[54], and protein catabolic process[55] (Additional file 1: Fig. S9). This suggests that the ssASm^{6}A may regulate the occurrence and development of lung cancer by affecting the function of lung cancer-related gene pathways.

Discussion

Recent research suggests the widespread presence of allele-specific m^{6}A modifications and their impact on disease susceptibility. In this study, a novel method called M6Allele was developed for detecting ASm^{6}A events using MeRIP-seq data, both in single-sample

analysis and in paired-sample comparison (differential ASm^{6}A). M6Allele integrates available information to determine ASm^{6}A extent in a given peak by meta-analysis across SNPs within IP and Input samples. Combining M6Allele with MeRIP-seq analysis tools in our pipeline enables precise visualization of the transcriptome-wide ASm^{6}A landscape.

Due to the absence of known phase information in most of the MeRIP-seq data, M6Allele utilizes a pseudo-phasing strategy to delineate the distribution of modified reads across various haplotypes. The pseudo-phasing strategy for inferring gene haplotypes may lead to non-conservative nominal p-values when calculating the statistical significance of ASm^{6}A. To assess this issue, the GPD was introduced to adjust the statistical significance level. The performance of simulated data demonstrated the robustness of this strategy, allowing M6Allele to accurately identify significant allele-specific imbalance events.

Unlike other existing algorithms, M6Allele does not identify SNP or mutation sites associated with ASm^{6}A. Instead, it employs a meta-analysis strategy at the peak level, integrating all SNPs information within each peak for ASm^{6}A estimation through a hierarchical Bayesian model. Using the MCMC process, the probability distribution of the odds ratio for the major allele haplotypes within each peak is sampled, constructing empirical statistical tests to identify significant ASm^{6}A events. This computational approach performs well across different parameters in MeRIP-seq experiments and compares favorably with other state-of-the-art tools. Additionally, the framework of M6Allele supports both within-sample and paired-sample ASm^{6}A analyses. The latter functionality allows the user to, for example, identify differential ASm^{6}A in tumor/normal comparisons, or to compare ASm^{6}A changes before and after treatment. These features make M6Allele more

suitable for identifying ASm^{6}A events under real experimental conditions. This study applied the M6Allele to identify ASm^{6}A events in pulmonary fibrosis and lung adenocarcinoma. The results demonstrated a significant association between the identified ASm^{6}A genes and these conditions, revealing the potential key role of ASm^{6}As in the development of these diseases. This also indicates that M6Allele can provide a reliable ASm^{6}A landscape for downstream experimental research.

Although M6Allele was originally designed for MeRIP-seq experiments, it is also applicable for peak detection and differential analysis of other RIP-seq data, such as m^{7}G or Ac^{4}C. However, since the peak-calling tools within the M6Allele pipeline are primarily optimized for MeRIP-seq data, users can alternatively upload peak-calling results from other tools to facilitate the analysis of ASM events across various RIP-seq datasets. In this study, we employed a pseudo-phasing strategy, which may introduce some deviation in MAF values, albeit insignificantly affecting events with marked allelic imbalances. Therefore, integrating gold standard haplotype data such as whole-genome sequencing data will be considered to enhance M6Allele's performance. Additionally, the gene dataset used to calibrate GPD for p-value correction comprises solely human genes. Nevertheless, given the homologous nature of gene expression, the p-value correction model remains applicable to studies involving other vertebrates. To ensure more precise assessments, our future endeavors will encompass a broader array of species within the M6Allele model, encompassing mice, fruit flies, yeast, and zebrafish.

Conclusions

This study showed that M6Allele is a powerful tool for detecting ASm^{6}A events using MeRIP-seq data, offering significant advantages in visualizing the transcriptome-wide ASm^{6}A landscape. The method's ability to handle both single-sample and paired-sample analyses provides versatility in identifying significant ASm^{6}A events under various experimental conditions. Applying M6Allele to pulmonary fibrosis and lung adenocarcinoma data highlighted its potential in uncovering the role of ASm^{6}As in disease development. While the pseudo-phasing strategy and haplotype reconstruction method have some limitations, introducing GPD for P-value adjustment ensures more accurate statistical significance assessments. This study paves the way for more comprehensive studies on the interplay between m^{6}A modifications and disease genetics, contributing valuable insights to the field. It sets the stage for more in-depth studies on how m^{6}A modifications interact with disease genetics, providing valuable insights into the field.

Methods

Overview of M6Allele

The comprehensive mathematical description and justification for M6Allele is provided in Supplementary Methods. Here, we offer a summary of M6Allele and its application in this manuscript.

The core algorithm of M6Allele is comprised of three functional modules: (1) a module that infers the genes with significant ASE event in RNA-seq samples (Fig. 1a), (2) a module designed to identify ASm^{6}A peaks from single MeRIP-seq sample, and (3) a module for detecting the differential ASm^{6}A peak between paired-samples.

Construction of ASE Determination module

We use genes as the units of ASE, defined as the combination of all exons that form individual transcript isoforms. M6Allele models the logarithm of odds ratio of major haplotype in a gene using a normal distribution.

However, the framework depends on specifying gene haplotypes, which may be unknown for MeRIP-seq data sets. Here, we refer to the voting-based pseudo-phasing strategy in MBASED[32] for haplotyping. When a gene contains at least one heterozygous exon SNP, we assume it to have two haplotypes. We then count the reads mapping to individual SNP in the Input sample and define the top two highest read counts of bases as the ‘major’ and ‘minor’ haplotypes for that site.

For a given gene, the following notation will be used upon describing the raw input:

n_j, total reads of the jth SNP site in the gene;

$x_{ma,j}$, the count of reads mapping to the major haplotype in SNP_j;

$x_{0,j}$, the theoretical read counts of the major haplotype at the SNPj without ASE, with a default value of $0.5*n_j$.

Accordingly, the standardized odds ratio ρ_j of major haplotype at individual SNPj can be represented as:

$$\rho_j = \frac{x_{ma,j}}{n_j - x_{ma,j}} / \frac{x_{0,j}}{n_j - x_{0,j}}. \quad (1)$$

The logarithm form of ρ_j is then computed as:

$$y_j = \ln(\rho_j) = \ln\left(\frac{x_{ma,j}}{n_j - x_{ma,j}}\right) - \ln\left(\frac{x_{0,j}}{n_j - x_{0,j}}\right). \quad (2)$$

Sequencing biases and subsequent analytic process such as reads alignment can usually cause fluctuations in observed read counts, making them deviated from theoretical values. Therefore, it's necessary to consider these fluctuations when estimating the logarithm of odds ratios for SNPs. To address this, we have introduced the one-way normal random-effects model (REM)[33] and assumed that each observed y_j in a gene are generated from the following process:

$$y_j \sim N\left(\theta_j, \sigma_j^2\right), \tag{3}$$

$$\theta_j \sim N\left(\mu, \tau^2\right), \tag{4}$$

$$\mu \sim Uniform(-\infty, +\infty), \tag{5}$$

$$\tau \sim scale - Inv - \chi^2(\varphi, s^2). \tag{6}$$

Notably, μ is the expected value of θ_j. Estimating μ provides the global log odds ratio for the major haplotype of the gene and serves as a measure of ASE extent. It is worth noting that, we found that the two parameters of the prior distribution for τ have a negligible impact on the identification performance of allele-specific events (Additional file 1: Fig. S7). Therefore, in the subsequent analysis, we set $\varphi = 5$ and $s^2 = 10$.

Using the improved Metropolis-Hastings (M-H) sampling method based on the Markov Chain Monte Carlo (MCMC) algorithm, we sample from the joint posterior distribution (Supplementary Methods shows the full derivation):

$$p\left(\theta_1, \cdots, \theta_j, \mu, \tau \mid y_1, y_2, \cdots, y_j\right), \tag{7}$$

and simultaneously their marginals:

$$p(\mu, \tau \mid y_1, y_2, \cdots, y_j) \tag{8}$$

and

$$p\left(\tau \mid y_1, y_2, \cdots, y_j\right). \tag{9}$$

We then compute the posterior means for μ, which we denote as μ_{ASE}, as the indicator of ASE.

Construction of ASm⁶A Determination module

The ASm^{6}A determination module is similar to the ASE determination module, using m^{6}A peaks as the meta-analysis unit and the SNP sites covered by each modification peak for hierarchical Bayesian model construction.

For a given peak, the following notation will be used to describe this step:

$n_j^{(m)}$, the total number of reads observed at that site in the IP sample;

$x_{ma,j}^{(m)}$, represents the read count for the major haplotype of the j_{th} SNP locus within the peak in the IP sample.

To eliminate the influence of ASE on ASm^{6}A identification, we will use the previously calculated gene ASE odds ratio μ_{ASE} as the background for calculating the ASm^{6}A odds ratio $\rho_j^{(m)}$, with the following equation:

$$\rho_j^{(m)} = \frac{x_{ma,j}^{(m)}}{n_j^{(m)} - x_{ma,j}^{(m)}} / e^{\mu_{ASE}} . \tag{10}$$

Furthermore, the equation for calculating the log odds ratio of ASm^{6}A is as follows:

$$y_j^{(m)} = \ln(\rho_j^{(m)}) = \ln(\frac{x_{\mathrm{ma},j}^{(m)}}{n_j^{(m)} - x_{ma,j}^{(m)}}) - \mu_{ASE} . \tag{11}$$

Similar to the ASE module, we constructed a hierarchical Bayesian model for each peak with the following process:

$$y_j^{(m)} \sim N(\theta_j^{(m)}, \sigma_j^{(m)^2}), \tag{12}$$

$$\theta_j^{(m)} \sim N\left(\mu^{(m)}, \tau^{(m)2}\right), \quad (13)$$

$$\mu^{(m)} \sim Uniform(-\infty, +\infty), \quad (14)$$

$$\tau^{(m)} \sim scale - Inv - \chi^2(\varphi, s^2). \quad (15)$$

Using the M-H sampling algorithm to estimate the parameters in the model, we can convert the calculated the posterior means for $\mu^{(m)}$ into the MAF of the peak to assess the tendency of allelic modification imbalance.

Construction of Paired-sample analysis module

In practical research, when samples from different groups originate from the same individual, they are referred to as paired samples, for example, the tumor and normal samples from the same patient. Researchers focus on intergroup differences not influenced by individual genetic information, such as sample-specific ASm^{6}A events. However, using a pseudo-phasing strategy may cause inconsistent haplotyping between samples when identifying ASm^{6}A separately for each sample, making it challenging to detect significant ASm^{6}A differences accurately. To address this issue, we have introduced a paired-sample analysis feature that builds upon the single-sample ASm^{6}A analysis. We describe the procedure here in terms of comparing a 'tumor' sample to a 'normal' sample, but the analysis can be done for any paired-samples. Initially, we identify m^{6}A peaks that overlap more than 50% in length between different samples as originating from the same modification event. The differential ASm^{6}A events between samples can be classified into the following scenarios:

1) A modification event is present in the tumor sample with allele-specificity but does not

appear in the normal sample; this is classified as a gain ASm^{6}A event in the tumor sample.

2) Conversely, it is considered a loss ASm^{6}A event in the tumor sample.

3) Another modification event is identified with allele-specificity in both tumor and normal samples, but shows differing major haplotypes; this is labeled as a gain event in tumor samples.

4) For modification event that shows allele-specificity in both tumor and normal samples, with the same major m^{6}A haplotype; it will be assessed for the significance of inter-sample differences using a hierarchical Bayesian model to estimate the odds ratio of the major m^{6}A haplotype. We consider the consensus heterozygous SNP sites within the combined regions of these peaks as available sites for the downstream analysis, ensuring consistent haplotyping between the two samples. For each SNP site, the odds ratio calculation formula is constructed as shown below:

$$\rho_j^s = \frac{\rho_{tumor,j}}{\rho_{normal,j}} \tag{16}$$

where, $\rho_{tumor,j} = \dfrac{y_{tumor,j}}{n_{tumor,j} - y_{tumor,j}} / e^{\mu_{tumor,b}}$, and $\rho_{normal,j} = \dfrac{y_{normal,j}}{n_{normal,j} - y_{normal,j}} / e^{u_{normal,b}}$.

Under the null hypothesis of no sample-specific ASm^{6}A event, we consider $\rho_{tumor,j} = \rho_{normal,j}$. We then construct a Bayesian model similar with the single-sample analysis for the M-H sampling the expected value of the natural logarithm of ρ_j^s.

Significance threshold for ASm^{6}A/ASE events

The hierarchical Bayesian models merely computed tendencies of allele-specific events. To identify significant allele-specific events, we need to construct a testing model. Here,

we developed a threshold calculation algorithm based on extreme value theory to assess the significance of allele-specific events. Details of the threshold calculation algorithm can be found in Supplementary Methods.

To distinguish significant allele-specific events, we are required to obtain the Minor Allele Frequency (MAF) distribution under the null hypothesis condition. Due to the lack of eligible real MeRIP-seq data meeting the criteria, we need to simulate sequencing data to obtain the read counts for major and minor alleles of SNPs without significant ASE or ASm^{6}A events. Since previous studies commonly fit the read distribution with a negative binomial distribution (NBD), we also introduce it here to fit the read count distribution for individual SNPs captured by sequencing.

When the total read count for the SNP$_i$ is N_i, the reads count x_{ij} for individual allele j (j can be 0 or 1) covering each SNP locus is assumed as follows:

$$x_{ij} \sim NB(\omega_i, k)\,. \tag{17}$$

Here, ω_i represents the theoretical read count of one haplotype at a SNP site without allele-specific events, so it can be calculated using $0.5N_i$. In addition, k is the dispersion parameter.

Next, we need to estimate k using appropriate sequencing data. Since most heterozygous somatic mutations on diploid genomes typically involve only one chromosome, genome-wide sequencing data for detecting genomic mutations theoretically lack allele imbalance and are suitable as background data for estimating k. To evaluate individual heterogeneity in actual sequencing data and determine the dispersion of read counts, we obtained whole-genome sequencing (WGS) data from the 1000 Genomes

database. We then tallied the read counts N_i at SNPi along with the read counts x_{i0} and x_{i1} for the alleles. Since each SNP from different individuals can be considered independently distributed, we integrated all the N_i and x_{ij} using maximum likelihood estimation to estimate the dispersion parameter k.

Subsequently, we simulated the total read counts for each SNP on every gene/peak as the parameter ω_i of the NBD. Given the varied gene expression patterns in the transcriptome, we established gene-specific FPKM distributions to enhance the fidelity of our simulated data reflecting true gene expression. We collected FPKM values for all genes from The Cancer Genome Atlas Program (TCGA)[56] and fitted their distributions for each gene using the Python package fitter (https://pypi.org/project/fitter/). Genes were classified into six categories with the distribution type of FPKM according to previous research[57]. To facilitate computation, we refitted the overall distribution of FPKM for each category and sampled from these distributions to simulate FPKM values for each gene within its respective class. Simultaneously, by simulating the library size of sequencing data, we further calculated the total read count $N_i^{'}$ for each SNP on the gene based on the simulated FPKM and gene length.

Based on the dispersion parameter k and $N_i^{'}$, we derived the NBD for the allelic reads of each SNP within the gene/peak. By sampling from the NBD, we simulated the counts of reads for major and minor alleles of every SNP, and obtained MAF for each gene/peak using M6Allele.

Given the rarity of allele-specific events, we assume they follow a tail distribution in genomic data. Thus, we introduced the Generalized Pareto Distribution (GPD), which

accurately models the tails of various distributions. In the categorization of different gene expression patterns, we estimated the tail distribution of MAF under the null hypothesis and computed the statistical significance thresholds.

The Implementation and Integration of M6Allele

We implemented the single- and paired-sample analyses described above in a JAR package called M6Allele. To enhance users' convenience, we provided a comprehensive pipeline for ASm^{6}A analysis using Docker. This pipeline integrates tools such as FastQC, fastp[58], STAR[59], VarScan[60], GATK[61], and MeTPeak[62] for quality control, alignment, SNP calling, and m^{6}A peak calling. While MeTPeak was used as the default peak-calling tool in this study, we have tested other peak-calling tools, such as TRESS[63] and exomePeak2[64], and MACS3[65], and confirmed that they are also compatible with M6Allele. By providing FASTQ sequencing files, gene annotation GTF files, and reference genome fasta files, users can automatically calculate allele-specific events for both gene expressions and m^{6}A modifications. The pipeline generates reports on MAF and ASE/ASm^{6}A p-values for each allele-specific event.

MeRIP-seq data collection and alignment

MeRIP-seq raw sequencing reads for pulmonary fibrosis and lung carcinoma were downloaded from the NCBI Gene Expression Omnibus[66] (GEO; https://www.ncbi.nlm.nih.gov/geo/; accession numbers GSE164151, GSE198288). FastX_Trimmer (version 0.0.13) and FastQC (version 0.11.9) was used to trim adaptors

and control read quality, respectively. Then, the clean reads were mapped to the human genome (GRCh38) using STAR[59] (version 2.7.6.a) with parameters set as --twopassMode Basic. SAMtools[67] was then utilized to filter for uniquely aligned sequences or select the highest-scoring alignment from multiple alignments.

Variant calling from the Input sample of MeRIP-seq data

VarScan[60] (version 2.3.9) was used to detect SNPs with a minimum VAF value of 0.05. Following this, BCFtools[67] (version 1.2.1) was employed to flag SNP positions with a reference allele depth less than 2 or within 3bp of an indel. Then, VCFtools[68] (version 0.1.17) was applied to filter out the flagged positions. The variants were retained if they matched the criteria: neither found in UCSC RepeatMasker microsatellites[69] nor in RNA editing sites (RADAR database[33]) but were contained in the dbSNP database[36] or the 1000 Genomes. Then we count the reads on the two alleles for each SNP. Only those variants that satisfied the minimum mapping reads on both alleles were considered as reliable candidate heterozygous sites (each allele $\geqslant$2, the sum of two alleles$\geqslant$10[29]).

m^6A peak calling from MeRIP-seq data

To obtain m^6A modification peaks, we utilized MeTPeak[62] (version 1.1) for peak calling with default parameter settings. By comparing with variant information, only m^6A peaks that contain variants were retained for allele-specific methylation analysis.

Comparison with the Other ASE or ASm^{6}A identification methods

In comparing ASE identification methods, we utilized GeneiASE [43] and MBASED [32] to to identify genes exhibiting significant ASE in simulated RNA-seq data. Leveraging the settings of true ASE events in the simulated data, we computed metrics such as Precision, Recall, FDR, F0.5, and F1 for the results obtained from GeneiASE, MBASED, and M6Allele, facilitating a thorough comparison.

For the comparison of ASm^{6}A identification tools, we executed the methods of ASPRIN and *Cao S* et al. according to their tutorials, adhering to the default parameter estimates as suggested by the authors. As these tools analyze individual SNP site, we aligned SNPs associated with ASm^{6}A identified by these tools with m^{6}A peaks. SNPs not aligning with the regions of m^{6}A peaks were excluded from the analysis. If any SNP within a peak was identified as having ASm^{6}A modification by ASPRIN or *Cao S* et al.'s algorithm, that peak was classified as ASm^{6}A modified, resulting in a positive outcome; otherwise, it was considered negative. Based on this strategy, we can get the accuracy of the prediction results for each peak and calculate the true positive rate (TPR) and false positive rate (FPR).

Gene Ontology Enrichment Analysis

We performed Gene Ontology Enrichment Analysis on genes with ASE or ASm^{6}A modifications using Metascape[42] with the “GO Biological Process” pathway dataset. A significance level of $P < 0.05$ was chosen as the threshold for statistical significance. Following this, we imported the GO pathway enrichment results into Cytoscape[70] and utilized the ClueGO[71] plugin to visualize the pathway networks.

Cell lines and cell culture

The human monocytic THP-1 cell line (#TIB-202), originally purchased from American Type Culture Collection (ATCC) by Dr. Shouheng Jin and provided for this study, was cultured in RPMI 1640 medium (Gibco, cat. C22400500BT) supplemented with 10% FBS and 1% glutamine.

MeRIP sequencing

Total RNA was extracted using TRIzol reagent (Invitrogen, USA) and assessed for quality using a NanoDrop and Bioanalyzer. Poly(A)-tailed RNA was purified using Dynabeads Oligo(dT)25 (Thermo Fisher, USA) and fragmented at 86°C for 7 minutes. Fragmented RNA was incubated with an m^6A-specific antibody (Cat# 202003, Synaptic Systems, Germany) in IP buffer to enrich m^6A-modified RNA. The RNA was reverse-transcribed into cDNA and converted into double-stranded DNA, followed by adapter ligation and size selection using AMPure XP beads. Libraries were amplified by PCR and sequenced on an Illumina NovaSeq™ 6000 platform (LC-Bio Technology Co., Ltd., Hangzhou, China) in paired-end 150 bp mode.

Sanger sequencing

To validate potential ASm^6A events, we initially selected 15 positive and 15 negative candidate sites based on M6Allele's predictions from the THP-1 cell line MeRIP-seq data. Ensure that the expression levels of the transcripts at the selected sites are sufficient for the validation, we filtered the sites by calculating the total read counts in the Input samples

and excluded sites with fewer than 25 reads (Additional file 2: Table S4). As a result, a total of 6 positive and 14 negative sites were used for validation. Primers targeting these sites were designed for both Input and IP samples, with detailed primer sequences provided in Additional file 2: Table S3. PCR products were gel-purified and subjected to Sanger sequencing. The sequencing chromatograms were processed using EditR software[72] to calculate the proportions of different nucleotides at the selected sites. Odds ratios for the major allele were calculated to compare nucleotide proportions between IP and input samples, with sites having an odds ratio greater than 1.2 classified as positive ASm^{6}A events.

Supplementary Information

Additional File 1:

Supplementary Methods and Supplementary Figure. S1-S10. **Fig. S1** Simulation data generation workflow. **Fig. S2** Comparison of the performance of M6Allele, ASPRIN, and *Cao S* et al. in different metrics. **Fig. S3** Performance comparison of different ASm^{6}A detection tools across various evaluation metrics on simulated datasets. **Fig. S4** Comparison of computational time for ASE and ASm^{6}A detection across different tools and sample sizes. **Fig. S5** IGV visualizations on simulated data. **Fig. S6** IGV visualizations of differential ASm^{6}A. **Fig. S7** ASE gene analysis results on pulmonary fibrosis dataset. **Fig. S8** Enrichment and pathway analysis of ASm^{6}A and ASE genes in pulmonary fibrosis. **Fig. S9** Pathway Analysis of Overlapping ssASm6A-Modified Genes and Their Interactors

Enriched in Lung Cancer-Related Pathways. **Fig. S10** Impact of scaled inverse chi-squared prior parameters on predicted major allele frequency.

Additional File 2:

Table S1. Results of model identification of ASE events on simulated data.

Table S2. Identification of ASM events in THP-1 cell line MeRIP-seq data using M6Allele, ASPRIN, and *Cao S* et al.

Table S3. Primers information of ASm^{6}A sites used for Sanger sequencing experiments.

Table S4. Base composition of ASm^{6}A sites in input and IP samples analyzed by Sanger sequencing.

Table S5. Identification of ASE events in pulmonary fibrosis dataset through single-sample analysis using M6Allele.

Table S6. Identification of ASM events in pulmonary fibrosis dataset through single-sample analysis using M6Allele.

Table S7. Identification of ASM events in lung adenocarcinoma dataset through paired-sample analysis using M6Allele.

Table S8. ssASm^{6}A genes in the lung adenocarcinoma dataset.

Acknowledgements

We thank Dr. Shouheng Jin for providing the THP-1 cell line used in this study.

Authors' contributions

X.L. and Y.X. conceived the project. Y.Z., L.T. and S.Z. developed the methodology and implemented the method. B.H. and Z.Z. helped with the design of methodology. X.L., Y.X. and J.R. wrote the paper. All authors read and approved the final manuscript.

Conflict of interest statement

The authors have declared that no competing interests exist.

Funding

This work was supported by National Key Research and Development Program of China [2023YFC2705900]; the National Natural Science Foundation of China [32200542,82301233]; the Young Elite Scientists Sponsorship Program by Guangzhou Association for Science and Technology [QT-2023-045]; the Guangdong Province Excellent Youth Team Project [2024B1515040009]; and the Discipline training, innovation and quality improvement engineering team project of Guangdong Pharmaceutical University [2024QZ02].

Data Availability

The raw THP-1 cell line MeRIP-seq data have been deposited in the Genome Sequence Archive[73] in National Genomics Data Center[74], China National Center for Bioinformation / Beijing Institute of Genomics, Chinese Academy of Sciences (GSA-Human: HRA009897) that are publicly accessible at https://ngdc.cncb.ac.cn/gsa-human. The raw Sanger sequencing results generated in this study have been deposited in

GigaDB and are accessible at files.gigadb.org/M6Aelle.

Availability of Source Code and Requirements

Project Name: M6Allele

Project Homepage: https://github.com/RenLabBioinformatics/M6Allele

Operating System(s): Platform independent

Programming Language: Java

Other Requirements: This pipeline integrates multiple tools, including FastQC, fastp[58], STAR[59], VarScan[60], GATK[61], and MeTPeak[62], for quality control, alignment, SNP calling, and m6A peak identification. All dependencies are prepackaged in the provided Docker image.

License: MIT License

RRID: SCR_026077

Bio.tools ID: biotools:m6allele

WorkflowHub DOI: https://doi.org/10.48546/WORKFLOWHUB.WORKFLOW.1223.1

The Docker image file of M6Allele, containing the JAR file and all necessary dependencies, can be downloaded from https://renlab.oss-cn-shenzhen.aliyuncs.com/M6Allele/m6allelepipe.tar.gz. Comprehensive installation and usage instructions are available on the project homepage: https://github.com/RenLabBioinformatics/M6Allele.

Ethics approval and consent to participate

This study includes the use of publicly available datasets obtained from open-access databases as well as newly generated sequencing data from the THP-1 cell line. The human monocytic THP-1 cell line (#TIB-202) was originally purchased from the American Type Culture Collection (ATCC) by Dr. Shouheng Jin, who kindly provided it for use in this study. The cells were cultured following standard protocols. As the THP-1 cell line is not classified as human research material, no additional ethics approval or written consent was required for this study.

Consent for publication

All authors have read and approved the final manuscript and consent to its publication in GigaScience.

Reference

1. Pastinen T. Genome-wide allele-specific analysis: insights into regulatory variation. Nat Rev Genet. 2010; 11:533-538.
2. Xu Q, Xiang Y, Wang Q, Wang L, Brind'Amour J, Bogutz AB, Zhang Y, Zhang B, Yu G, Xia W, et al. SETD2 regulates the maternal epigenome, genomic imprinting and embryonic development. Nat Genet. 2019; 51:844-856.
3. Bonthuis PJ, Huang WC, Stacher Horndli CN, Ferris E, Cheng T, Gregg C. Noncanonical Genomic Imprinting Effects in Offspring. Cell Rep. 2015; 12:979-991.
4. Sveen A, Johannessen B, Eilertsen IA, Rosok BI, Gulla M, Eide PW, Bruun J, Kryeziu K, Meza-Zepeda LA, Myklebost O, et al. The expressed mutational landscape of microsatellite stable

colorectal cancers. Genome Med. 2021; 13:142.

5. Gendrel AV, Marion-Poll L, Katoh K, Heard E. Random monoallelic expression of genes on autosomes: Parallels with X-chromosome inactivation. Semin Cell Dev Biol. 2016; 56:100-110.

6. Reinius B, Sandberg R. Random monoallelic expression of autosomal genes: stochastic transcription and allele-level regulation. Nat Rev Genet. 2015; 16:653-664.

7. van Ekelenburg YS, Hornslien KS, Van Hautegem T, Fendrych M, Van Isterdael G, Bjerkan KN, Miller JR, Nowack MK, Grini PE. Spatial and temporal regulation of parent-of-origin allelic expression in the endosperm. Plant Physiol. 2023; 191:986-1001.

8. Barlow DP, Bartolomei MS. Genomic imprinting in mammals. Cold Spring Harb Perspect Biol. 2014; 6.

9. Kravitz SN, Gregg C. New subtypes of allele-specific epigenetic effects: implications for brain development, function and disease. Curr Opin Neurobiol. 2019; 59:69-78.

10. Sigurdsson MI, Saddic L, Heydarpour M, Chang TW, Shekar P, Aranki S, Couper GS, Shernan SK, Seidman JG, Body SC, Muehlschlegel JD. Allele-specific expression in the human heart and its application to postoperative atrial fibrillation and myocardial ischemia. Genome Med. 2016; 8:127.

11. Gyorgy B, Nist-Lund C, Pan B, Asai Y, Karavitaki KD, Kleinstiver BP, Garcia SP, Zaborowski MP, Solanes P, Spataro S, et al. Allele-specific gene editing prevents deafness in a model of dominant progressive hearing loss. Nat Med. 2019; 25:1123-1130.

12. Sen A, Huo Y, Elster J, Zage PE, McVicker G. Allele-specific expression reveals genes with recurrent cis-regulatory alterations in high-risk neuroblastoma. Genome Biol. 2022; 23:71.

13. Shetty A, Seo JH, Bell CA, O'Connor EP, Pomerantz MM, Freedman ML, Gusev A. Allele-specific

epigenetic activity in prostate cancer and normal prostate tissue implicates prostate cancer risk mechanisms. Am J Hum Genet. 2021; 108:2071-2085.

14. Guo Y, Feng YF, Yang GG, Jia Y, He J, Wu ZY, Liao HR, Wei QX, Xue LJ. Allele-specific DNA methylation and gene expression during shoot organogenesis in tissue culture of hybrid poplar. Hortic Res. 2024; 11:uhae027.

15. Xuan A, Song Y, Bu C, Chen P, El-Kassaby YA, Zhang D. Changes in DNA Methylation in Response to 6-Benzylaminopurine Affect Allele-Specific Gene Expression in Populus Tomentosa. Int J Mol Sci. 2020; 21.

16. Zhang Y, Rohde C, Reinhardt R, Voelcker-Rehage C, Jeltsch A. Non-imprinted allele-specific DNA methylation on human autosomes. Genome Biol. 2009; 10:R138.

17. Zheng HX, Zhang XS, Sui N. Advances in the profiling of N(6)-methyladenosine (m(6)A) modifications. Biotechnol Adv. 2020; 45:107656.

18. Han X, Guo J, Fan Z. Interactions between m6A modification and miRNAs in malignant tumors. Cell Death Dis. 2021; 12:598.

19. Feng ZH, Liang YP, Cen JJ, Yao HH, Lin HS, Li JY, Liang H, Wang Z, Deng Q, Cao JZ, et al. m6A-immune-related lncRNA prognostic signature for predicting immune landscape and prognosis of bladder cancer. J Transl Med. 2022; 20:492.

20. Du A, Li S, Zhou Y, Disoma C, Liao Y, Zhang Y, Chen Z, Yang Q, Liu P, Liu S, et al. M6A-mediated upregulation of circMDK promotes tumorigenesis and acts as a nanotherapeutic target in hepatocellular carcinoma. Mol Cancer. 2022; 21:109.

21. Liu H, Zheng J, Liao A. The regulation and potential roles of m6A modifications in early embryonic development and immune tolerance at the maternal-fetal interface. Front Immunol.

2022; 13:988130.

22. Yang Z, Cai Z, Yang C, Luo Z, Bao X. ALKBH5 regulates STAT3 activity to affect the proliferation and tumorigenicity of osteosarcoma via an m6A-YTHDF2-dependent manner. EBioMedicine. 2022; 80:104019.

23. Kasowitz SD, Ma J, Anderson SJ, Leu NA, Xu Y, Gregory BD, Schultz RM, Wang PJ. Nuclear m6A reader YTHDC1 regulates alternative polyadenylation and splicing during mouse oocyte development. PLoS Genet. 2018; 14:e1007412.

24. Yin H, Zhang X, Yang P, Zhang X, Peng Y, Li D, Yu Y, Wu Y, Wang Y, Zhang J, et al. RNA m6A methylation orchestrates cancer growth and metastasis via macrophage reprogramming. Nat Commun. 2021; 12:1394.

25. Azzam SK, Alsafar H, Sajini AA. FTO m6A Demethylase in Obesity and Cancer: Implications and Underlying Molecular Mechanisms. Int J Mol Sci. 2022; 23.

26. Xiong X, Hou L, Park YP, Molinie B, Consortium GT, Gregory RI, Kellis M. Genetic drivers of m(6)A methylation in human brain, lung, heart and muscle. Nat Genet. 2021; 53:1156-1165.

27. Olazagoitia-Garmendia A, Rojas-Marquez H, Sebastian-delaCruz M, Agirre-Lizaso A, Ochoa A, Mendoza-Gomez LM, Perugorria MJ, Bujanda L, Madrigal AH, Santin I, Castellanos-Rubio A. m(6)A Methylated Long Noncoding RNA LOC339803 Regulates Intestinal Inflammatory Response. Adv Sci (Weinh). 2024; 11:e2307928.

28. Olazagoitia-Garmendia A, Zhang L, Mera P, Godbout JK, Sebastian-DelaCruz M, Garcia-Santisteban I, Mendoza LM, Huerta A, Irastorza I, Bhagat G, et al. Gluten-induced RNA methylation changes regulate intestinal inflammation via allele-specific XPO1 translation in epithelial cells. Gut. 2022; 71:68-76.

29. Cao S, Zhu H, Cui J, Liu S, Li Y, Shi J, Mo J, Wang Z, Wang H, Hu J, et al. Allele-specific RNA N (6)-methyladenosine modifications reveal functional genetic variants in human tissues. Genome Res. 2023; 33:1369-1380.

30. Bahrami-Samani E, Xing Y. Discovery of Allele-Specific Protein-RNA Interactions in Human Transcriptomes. Am J Hum Genet. 2019; 104:492-502.

31. Guk JY, Jang MJ, Choi JW, Lee YM, Kim S. De novo phasing resolves haplotype sequences in complex plant genomes. Plant Biotechnol J. 2022; 20:1031-1041.

32. Castel SE, Levy-Moonshine A, Mohammadi P, Banks E, Lappalainen T. Tools and best practices for data processing in allelic expression analysis. Genome Biol. 2015; 16:195.

33. Ramaswami G, Li JB. RADAR: a rigorously annotated database of A-to-I RNA editing. Nucleic Acids Res. 2014; 42:D109-113.

34. Mayba O, Gilbert HN, Liu J, Haverty PM, Jhunjhunwala S, Jiang Z, Watanabe C, Zhang Z. MBASED: allele-specific expression detection in cancer tissues and cell lines. Genome Biol. 2014; 15:405.

35. Borenstein M, Hedges LV, Higgins JP, Rothstein HR. A basic introduction to fixed-effect and random-effects models for meta-analysis. Res Synth Methods. 2010; 1:97-111.

36. Sherry ST, Ward MH, Kholodov M, Baker J, Phan L, Smigielski EM, Sirotkin K. dbSNP: the NCBI database of genetic variation. Nucleic Acids Res. 2001; 29:308-311.

37. Wang C, Chen G. A new hybrid estimation method for the generalized pareto distribution. Communications in Statistics-Theory and Methods. 2016; 45:4285-4294.

38. Albaradei S, Thafar M, Alsaedi A, Van Neste C, Gojobori T, Essack M, Gao X. Machine learning and deep learning methods that use omics data for metastasis prediction. Comput Struct Biotechnol J. 2021; 19:5008-5018.

39. Frazee AC, Jaffe AE, Langmead B, Leek JT. Polyester: simulating RNA-seq datasets with differential transcript expression. Bioinformatics. 2015; 31:2778-2784.

40. Edsgard D, Iglesias MJ, Reilly SJ, Hamsten A, Tornvall P, Odeberg J, Emanuelsson O. GeneiASE: Detection of condition-dependent and static allele-specific expression from RNA-seq data without haplotype information. Sci Rep. 2016; 6:21134.

41. Sokolova M, Lapalme G. A systematic analysis of performance measures for classification tasks. Information processing & management. 2009; 45:427-437.

42. Zhou Y, Zhou B, Pache L, Chang M, Khodabakhshi AH, Tanaseichuk O, Benner C, Chanda SK. Metascape provides a biologist-oriented resource for the analysis of systems-level datasets. Nat Commun. 2019; 10:1523.

43. Pinero J, Ramirez-Anguita JM, Sauch-Pitarch J, Ronzano F, Centeno E, Sanz F, Furlong LI. The DisGeNET knowledge platform for disease genomics: 2019 update. Nucleic Acids Res. 2020; 48:D845-D855.

44. Szklarczyk D, Kirsch R, Koutrouli M, Nastou K, Mehryary F, Hachilif R, Gable AL, Fang T, Doncheva NT, Pyysalo S. The STRING database in 2023: protein–protein association networks and functional enrichment analyses for any sequenced genome of interest. Nucleic acids research. 2023; 51:D638-D646.

45. Wang Z, Liu Y, Chen F, Liao H, Wang X, Guo Z, Wang Z. Feasibility and mechanism analysis of Reduning in the prevention of sepsis-induced pulmonary fibrosis. Front Pharmacol. 2022; 13:1079511.

46. Rajesh R, Atallah R, Barnthaler T. Dysregulation of metabolic pathways in pulmonary fibrosis. Pharmacol Ther. 2023; 246:108436.

47. Guan S, Zhou J. CXCR7 attenuates the TGF-beta-induced endothelial-to-mesenchymal transition and pulmonary fibrosis. Mol Biosyst. 2017; 13:2116-2124.

48. Grimminger F, Gunther A, Vancheri C. The role of tyrosine kinases in the pathogenesis of idiopathic pulmonary fibrosis. Eur Respir J. 2015; 45:1426-1433.

49. Scruggs AM, Koh HB, Tripathi P, Leeper NJ, White ES, Huang SK. Loss of CDKN2B promotes fibrosis via increased fibroblast differentiation rather than proliferation. Am J Respir Cell Mol Biol. 2018; 59:200-214.

50. Liu Y, Yang D, Liu T, Chen J, Yu J, Yi P. N6-methyladenosine-mediated gene regulation and therapeutic implications. Trends Mol Med. 2023; 29:454-467.

51. Li K, Peng ZY, Wang R, Li X, Du N, Liu DP, Zhang J, Zhang YF, Ma L, Sun Y, et al. Enhancement of TKI sensitivity in lung adenocarcinoma through m6A-dependent translational repression of Wnt signaling by circ-FBXW7. Mol Cancer. 2023; 22:103.

52. Fang H, Sun Q, Zhou J, Zhang H, Song Q, Zhang H, Yu G, Guo Y, Huang C, Mou Y, et al. m(6)A methylation reader IGF2BP2 activates endothelial cells to promote angiogenesis and metastasis of lung adenocarcinoma. Mol Cancer. 2023; 22:99.

53. Zhang JX, Huang PJ, Wang DP, Yang WY, Lu J, Zhu Y, Meng XX, Wu X, Lin QH, Lv H, et al. m(6)A modification regulates lung fibroblast-to-myofibroblast transition through modulating KCNH6 mRNA translation. Mol Ther. 2021; 29:3436-3448.

54. Guo L, Liu Z, Tang X. Overexpression of SLFN5 induced the epithelial-mesenchymal transition in human lung cancer cell line A549 through beta-catenin/Snail/E-cadherin pathway. Eur J Pharmacol. 2019; 862:172630.

55. Wang X, Chen X, Liu H. Expression and Bioinformatics-Based Functional Analysis of UAP1 in

Lung Adenocarcinoma. Cancer Manag Res. 2020; 12:12111-12121.

56. Cancer Genome Atlas Research N, Weinstein JN, Collisson EA, Mills GB, Shaw KR, Ozenberger BA, Ellrott K, Shmulevich I, Sander C, Stuart JM. The Cancer Genome Atlas Pan-Cancer analysis project. Nat Genet. 2013; 45:1113-1120.

57. de Torrente L, Zimmerman S, Suzuki M, Christopeit M, Greally JM, Mar JC. The shape of gene expression distributions matter: how incorporating distribution shape improves the interpretation of cancer transcriptomic data. BMC Bioinformatics. 2020; 21:562.

58. Chen S, Zhou Y, Chen Y, Gu J. fastp: an ultra-fast all-in-one FASTQ preprocessor. Bioinformatics. 2018; 34:i884-i890.

59. Dobin A, Davis CA, Schlesinger F, Drenkow J, Zaleski C, Jha S, Batut P, Chaisson M, Gingeras TR. STAR: ultrafast universal RNA-seq aligner. Bioinformatics. 2013; 29:15-21.

60. Koboldt DC, Larson DE, Wilson RK. Using VarScan 2 for Germline Variant Calling and Somatic Mutation Detection. Curr Protoc Bioinformatics. 2013; 44:15 14 11-17.

61. McKenna A, Hanna M, Banks E, Sivachenko A, Cibulskis K, Kernytsky A, Garimella K, Altshuler D, Gabriel S, Daly M, DePristo MA. The Genome Analysis Toolkit: a MapReduce framework for analyzing next-generation DNA sequencing data. Genome Res. 2010; 20:1297-1303.

62. Cui X, Meng J, Zhang S, Chen Y, Huang Y. A novel algorithm for calling mRNA m6A peaks by modeling biological variances in MeRIP-seq data. Bioinformatics. 2016; 32:i378-i385.

63. Guo Z, Shafik AM, Jin P, Wu H. Differential RNA methylation analysis for MeRIP-seq data under general experimental design. Bioinformatics. 2022; 38:4705-4712.

64. Meng J, Lu Z, Liu H, Zhang L, Zhang S, Chen Y, Rao MK, Huang Y. A protocol for RNA methylation differential analysis with MeRIP-Seq data and exomePeak R/Bioconductor package. Methods.

2014; 69:274-281.

65. Zhang Y, Liu T, Meyer CA, Eeckhoute J, Johnson DS, Bernstein BE, Nusbaum C, Myers RM, Brown M, Li W, Liu XS. Model-based analysis of ChIP-Seq (MACS). Genome Biol. 2008; 9:R137.

66. Clough E, Barrett T. The Gene Expression Omnibus Database. Methods Mol Biol. 2016; 1418:93-110.

67. Danecek P, Bonfield JK, Liddle J, Marshall J, Ohan V, Pollard MO, Whitwham A, Keane T, McCarthy SA, Davies RM, Li H. Twelve years of SAMtools and BCFtools. Gigascience. 2021; 10.

68. Danecek P, Auton A, Abecasis G, Albers CA, Banks E, DePristo MA, Handsaker RE, Lunter G, Marth GT, Sherry ST, et al. The variant call format and VCFtools. Bioinformatics. 2011; 27:2156-2158.

69. Tarailo-Graovac M, Chen N. Using RepeatMasker to identify repetitive elements in genomic sequences. Curr Protoc Bioinformatics. 2009; Chapter 4:4 10 11-14 10 14.

70. Shannon P, Markiel A, Ozier O, Baliga NS, Wang JT, Ramage D, Amin N, Schwikowski B, Ideker T. Cytoscape: a software environment for integrated models of biomolecular interaction networks. Genome Res. 2003; 13:2498-2504.

71. Bindea G, Mlecnik B, Hackl H, Charoentong P, Tosolini M, Kirilovsky A, Fridman WH, Pages F, Trajanoski Z, Galon J. ClueGO: a Cytoscape plug-in to decipher functionally grouped gene ontology and pathway annotation networks. Bioinformatics. 2009; 25:1091-1093.

72. Kluesner MG, Nedveck DA, Lahr WS, Garbe JR, Abrahante JE, Webber BR, Moriarity BS. EditR: A Method to Quantify Base Editing from Sanger Sequencing. CRISPR J. 2018; 1:239-250.

73. Chen T, Chen X, Zhang S, Zhu J, Tang B, Wang A, Dong L, Zhang Z, Yu C, Sun Y, et al. The Genome Sequence Archive Family: Toward Explosive Data Growth and Diverse Data Types. Genomics

Proteomics Bioinformatics. 2021; 19:578-583.
74. Members C-N, Partners. Database Resources of the National Genomics Data Center, China National Center for Bioinformation in 2024. Nucleic Acids Res. 2024; 52:D18-D32.

Figure Legend

Fig. 1 ASm^{6}As analysis pipeline. **a** Schematic diagram of the M6Allele model. **b** ASm^{6}As identification pipeline based on Docker.

Fig. 2 Performance comparison of different tools of ASE identification on simulated datasets. **a** The precision rates in ASE analysis between M6Allele, MBASED, and GeneiASE with different numbers of biological replicates. **b** The recall rates in ASE analysis between M6Allele, MBASED, and GeneiASE with different numbers of biological replicates. **c** The false discovery rates in ASE analysis between M6Allele, MBASED, and GeneiASE with different numbers of biological replicates. **d** The F0.5 scores in ASE analysis between M6Allele, MBASED, and GeneiASE with different numbers of biological replicates. **e** The F1 scores in ASE analysis between M6Allele, MBASED, and GeneiASE with different numbers of biological replicates. **f** Visualization of the number of reads covered by allele-specific expressed gene versus non-allele-specific expressed gene.

Fig. 3 Evaluation of the M6Allele algorithm performance on various metrics. **a** The error in ASm^{6}A analysis in M6Allele with different sequencing lengths. **b** The error in ASm^{6}A analysis in M6Allele with different library sizes. **c** The error in ASm^{6}A analysis in M6Allele

with different FPKMs. **d** The error in ASm^{6}A analysis in M6Allele with different numbers of SNP sites covered by each modification peak. **e** The error in ASm^{6}A analysis in M6Allele with different numbers of biological replicates. **f** The performance evaluation and comparison of M6Allele, ASPRIN, and the algorithm developed by *Cao S* et al. in the simulated MeRIP-seq dataset. **g** The performance evaluation and comparison of M6Allele, ASPRIN, and the algorithm developed by *Cao S* et al. using the MeRIP-seq data from THP-1 cell line, followed by Sanger sequencing. **h** Visualization of the number of reads covered by ASm^{6}A peak versus non-ASm^{6}A peak. **i** Identification of sample-specific ASm^{6}A events in the simulated paired-samples dataset.

Fig. 4 Analysis results of M6Allele on pulmonary fibrosis dataset. **a** The chromosomal distribution of genes with ASE. **b** The chromosomal distribution of genes with ASm^{6}A. **c** The results of GO enrichment analysis with ASm^{6}A-Gain genes. **d** The results of GO enrichment analysis with ASm^{6}A-Loss genes. **e** The hypergeometric test results for ASm^{6}A-Gain/Loss events related to disease associated genes in pulmonary fibrosis and lung adenocarcinoma. The blue bar represents the odds ratio of genes associated with pulmonary fibrosis observed in ASm^{6}A-Gain/Loss genes, where 'a' denotes the overlap between ASm^{6}A-Gain/Loss genes and pulmonary fibrosis-associated genes, 'b' represents ASm^{6}A-Gain/Loss genes exclusively, 'c' denotes genes exclusively associated with pulmonary fibrosis, and 'd' represents genes that do not belong to either category. The red bar represents the odds ratio of genes associated with lung cancer observed in ssASm^{6}A-Gain/Loss genes of tumor samples. **f** The hypergeometric test results for ASm^{6}A-regulated

events related to disease associated genes in pulmonary fibrosis and lung adenocarcinoma. The green bar represents the odds ratio of ASm^{6}A-regulated genes observed in pulmonary fibrosis-associated genes, where 'a' denotes the overlap between both gene categories, 'b' represents genes exclusively associated with pulmonary fibrosis, 'c' denotes genes exclusively regulated by ASm^{6}A, and 'd' represents genes that do not belong to either category. The meanings of 'a' to 'd' in the remaining bars are analogous to those in above two bars. The purple bar represents the odds ratio of genes associated with lung adenocarcinoma observed in ssASm^{6}A-regulated genes of tumor samples.

Fig. 5 Analysis results of M6Allele on lung adenocarcinoma dataset. **a** ssASm^{6}A events identified by the single-sample analysis strategy. **b** Comparison of the ssASm^{6}A events from the single-sample analysis and the paired-sample analysis. **c** ssASm^{6}A modified genes in tumor samples. **d** ssASm^{6}A modified genes in normal samples.

Figure 1

Click here to access/download;Figure;Figure 1.pdf

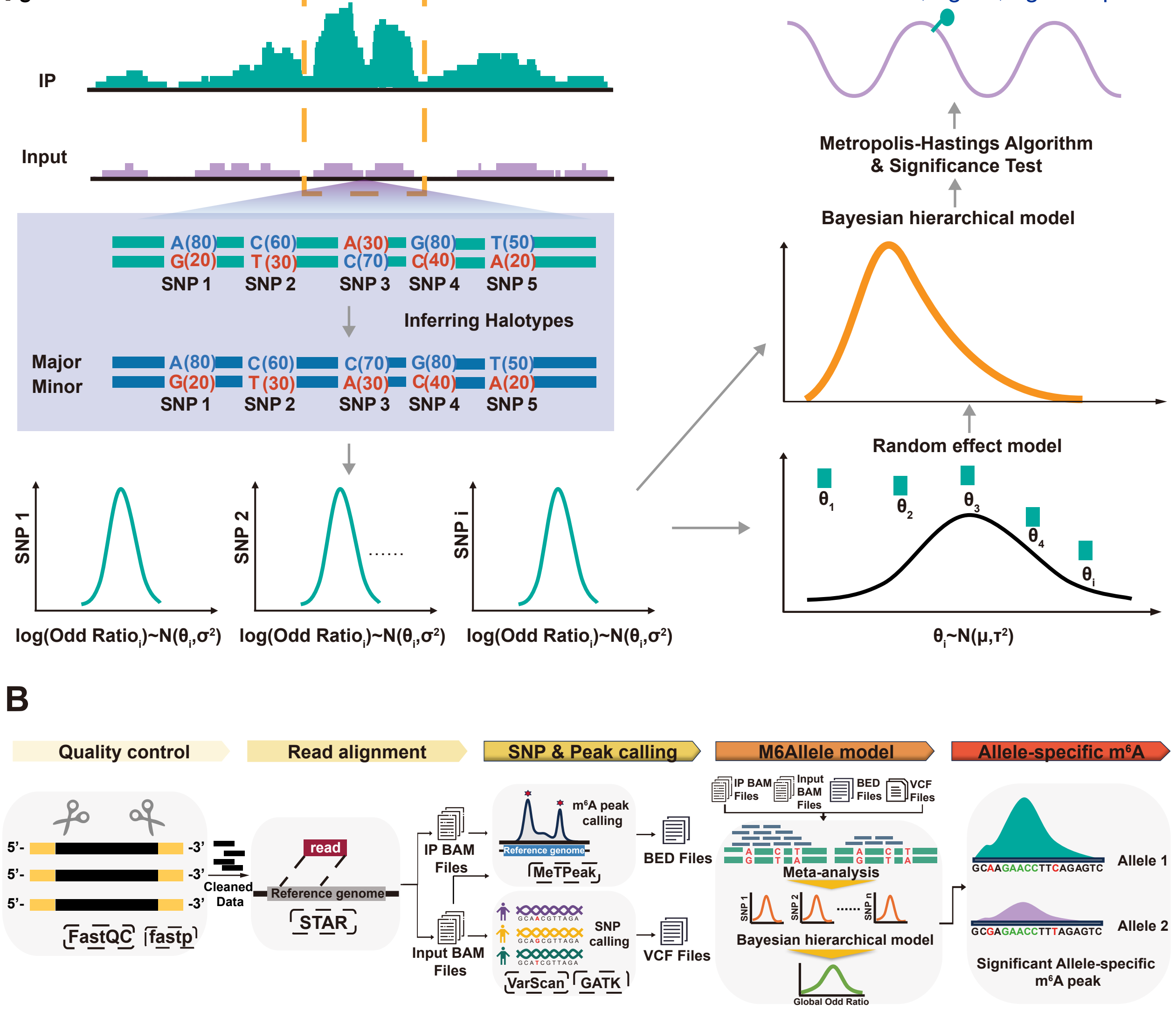

Figure 2

Click here to access/download;Figure;Figure 2.pdf

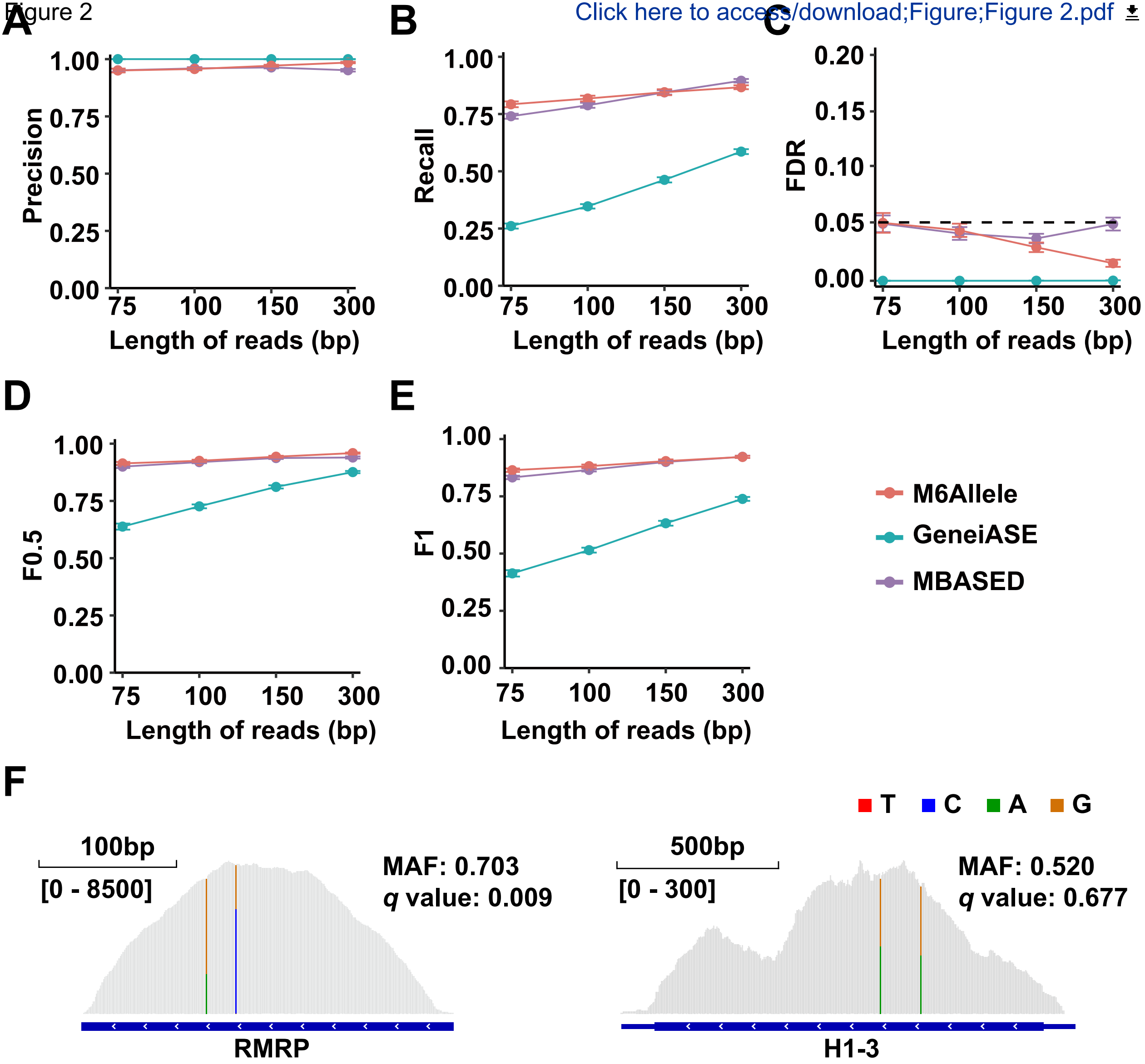

Figure 3

Click here to access/download;Figure;Figure 3.pdf

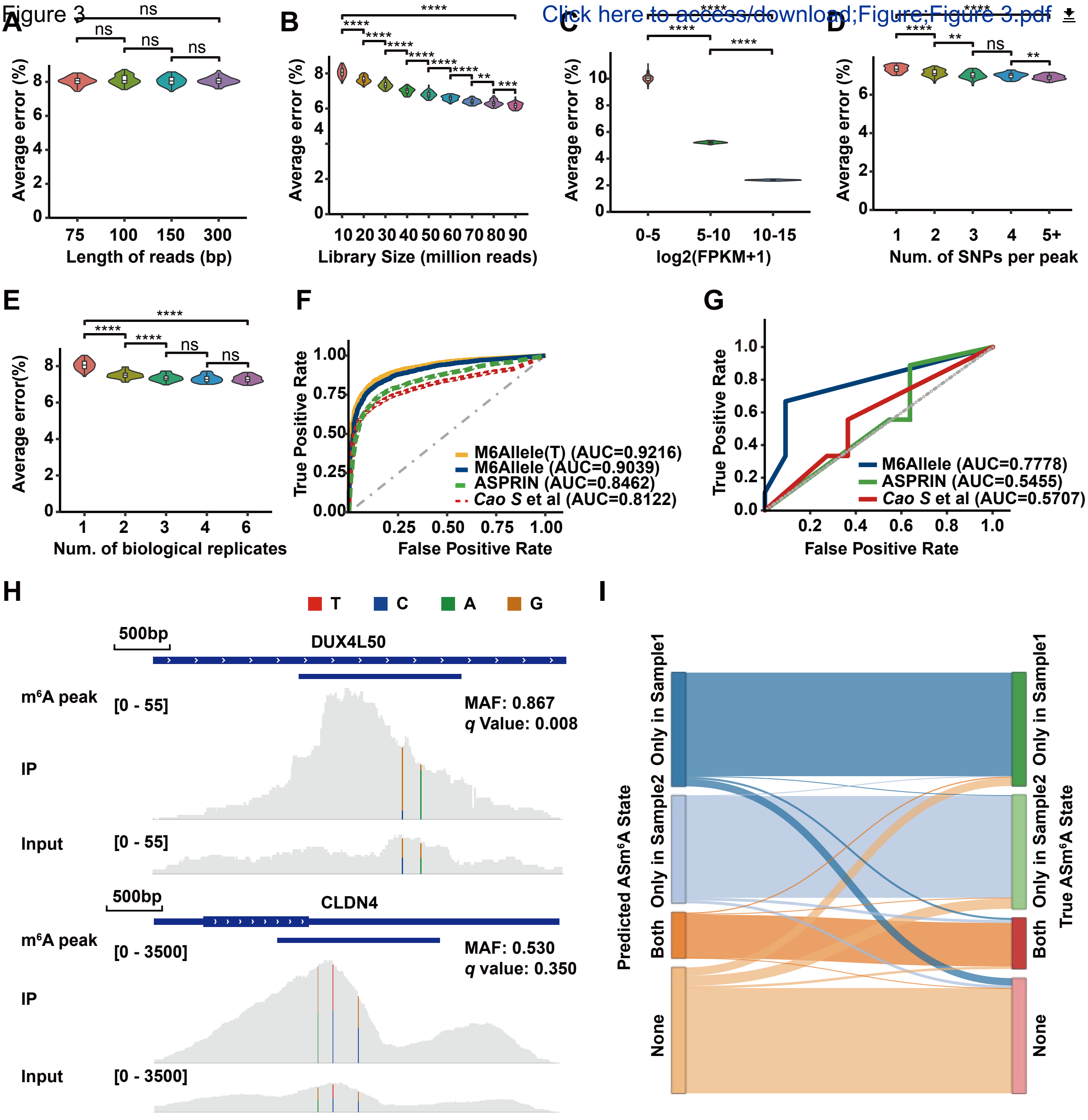

Figure 4

Click here to access/download;Figure;Figure 4.pdf

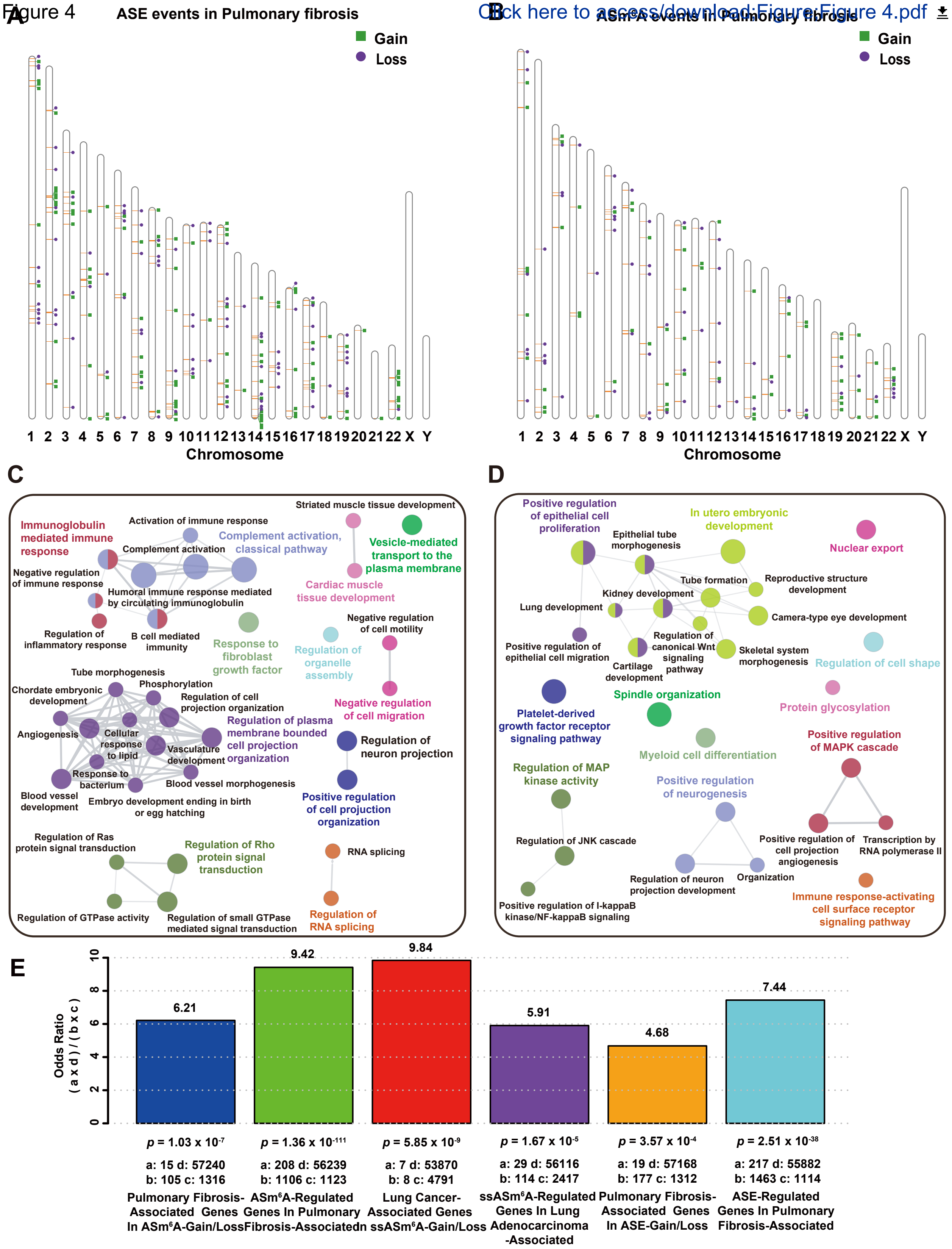

Figure 5

Click here to access/download;Figure

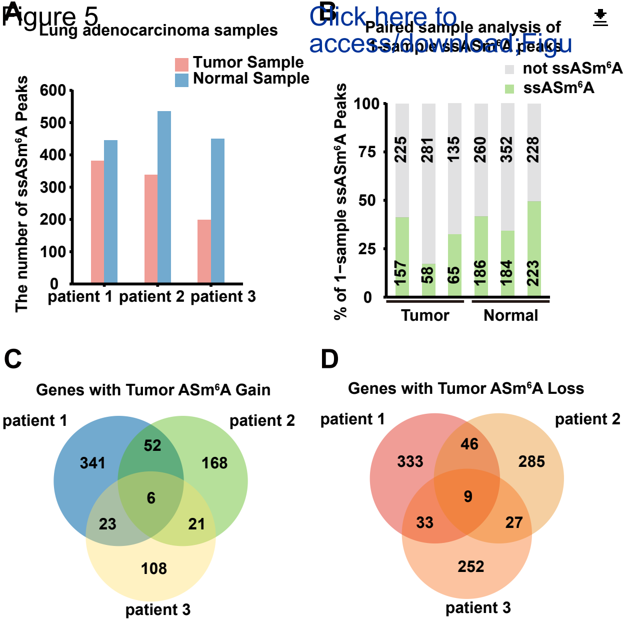

Figure S1

Click here to access/download;Figure;Figure S1.pdf

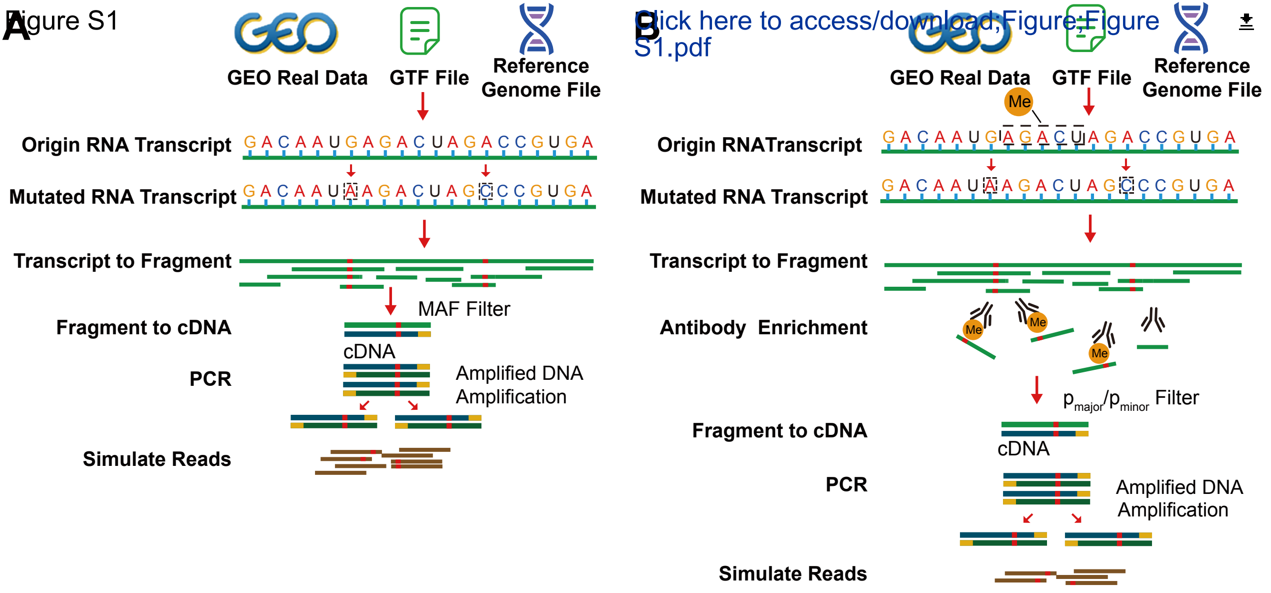

Figure S2

Click here to access/download;Figure;Figure S2.pdf

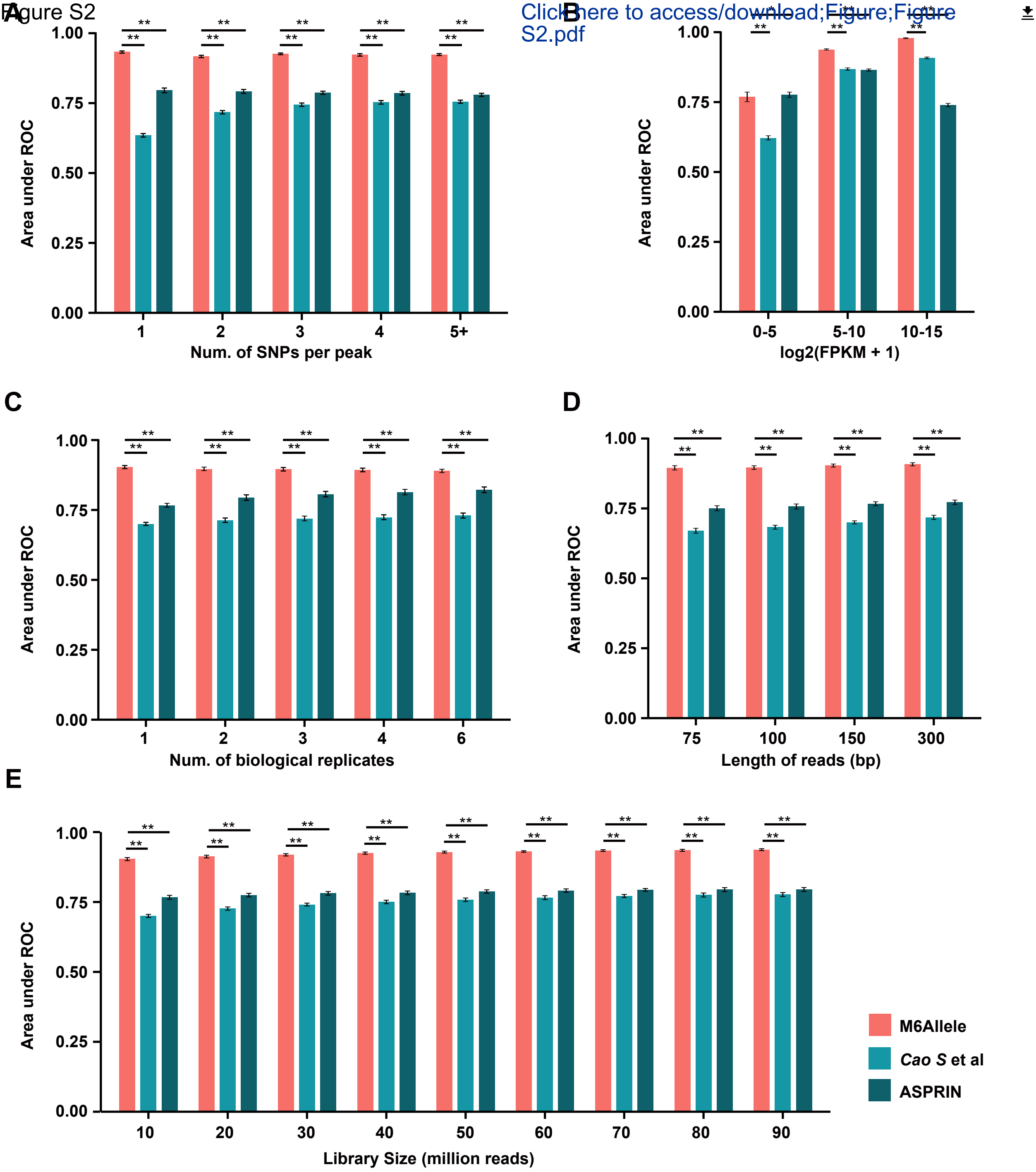

Figure S3

Click here to access/download;Figure;Figure S3.pdf

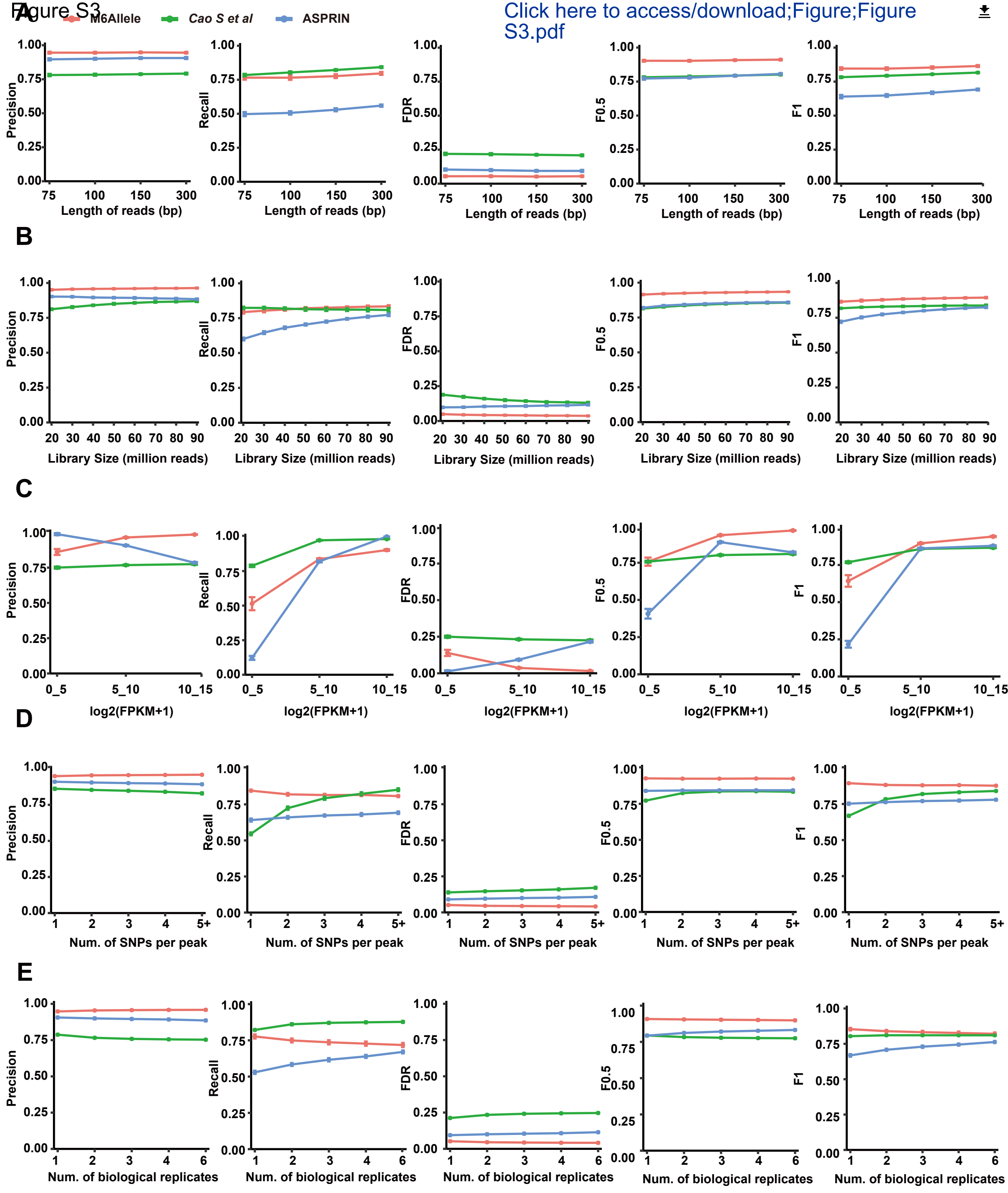

Figure S4

Click here to access/download;Figure;Figure S4.pdf

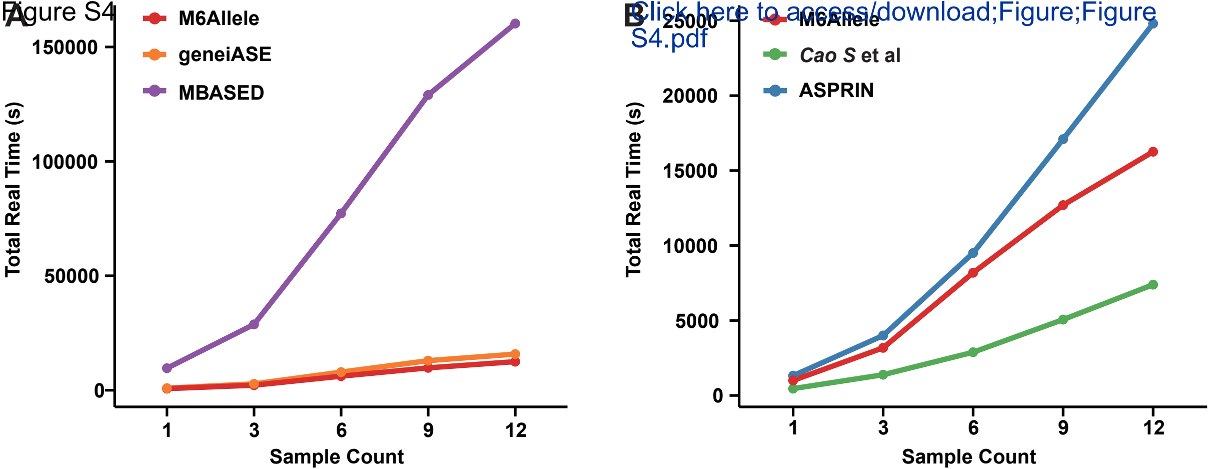

Figure S5

Click here to access/download;Figure;Figure S5.pdf

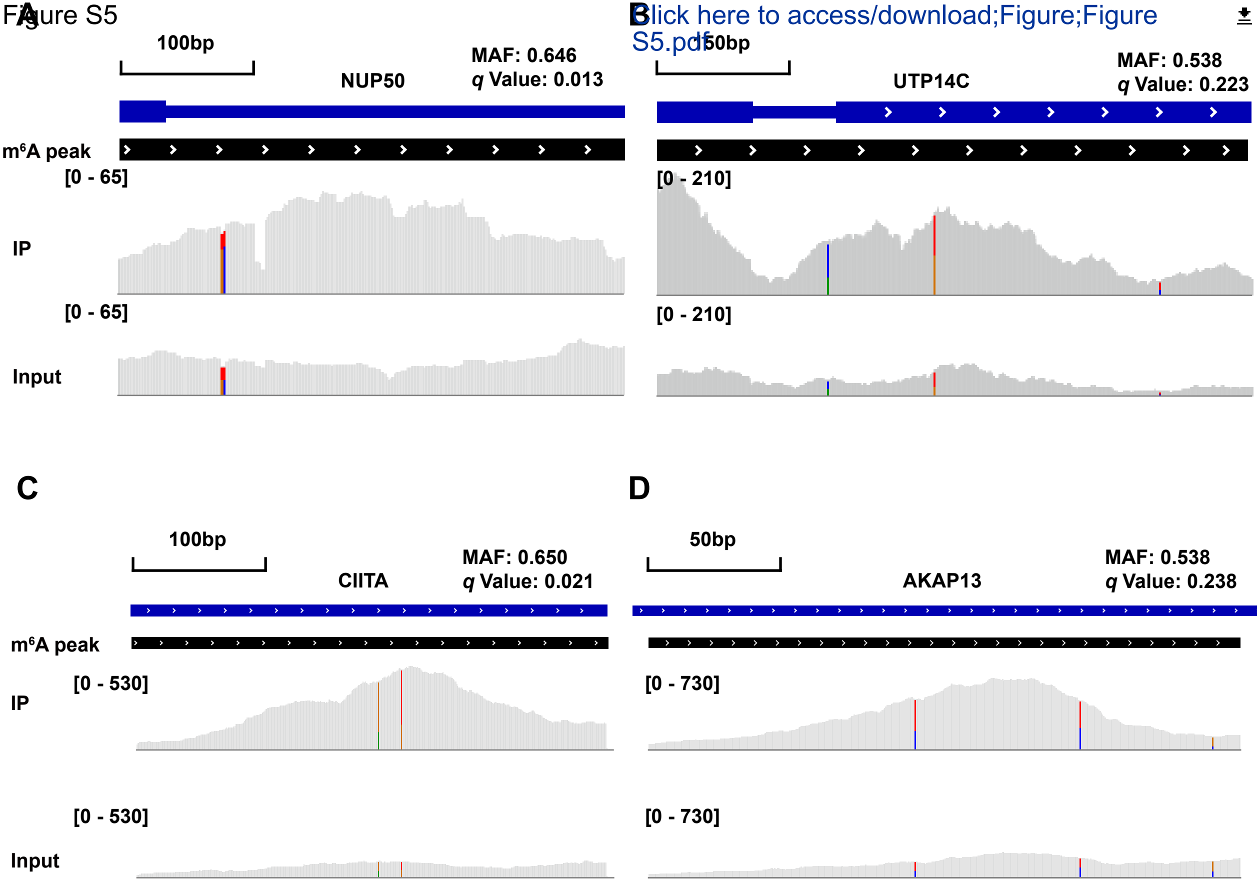

Figure S6

Click here to access/download;Figure;Figure S6.pdf

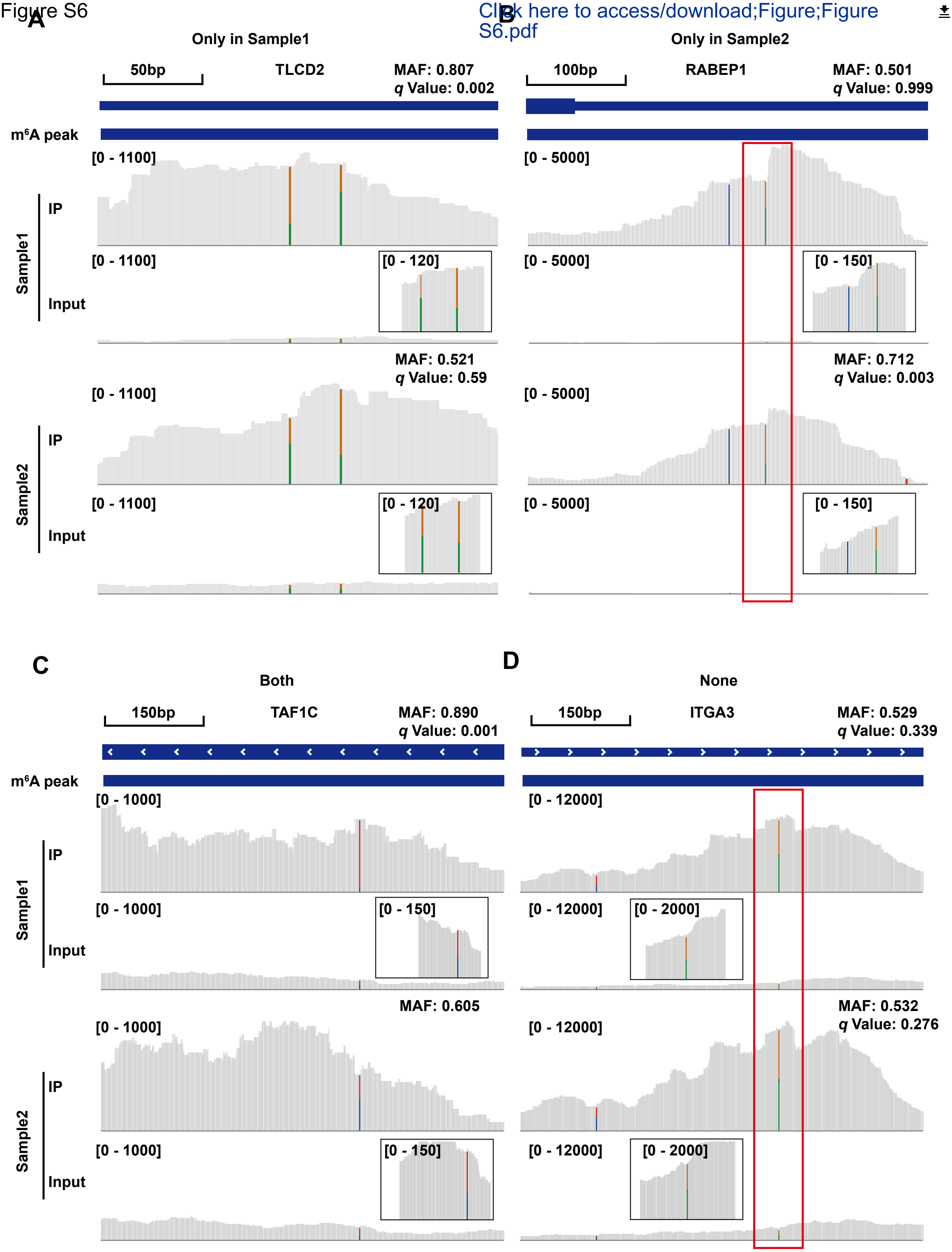

Figure S7

Click here to access/download;Figure;Figure S7.pdf

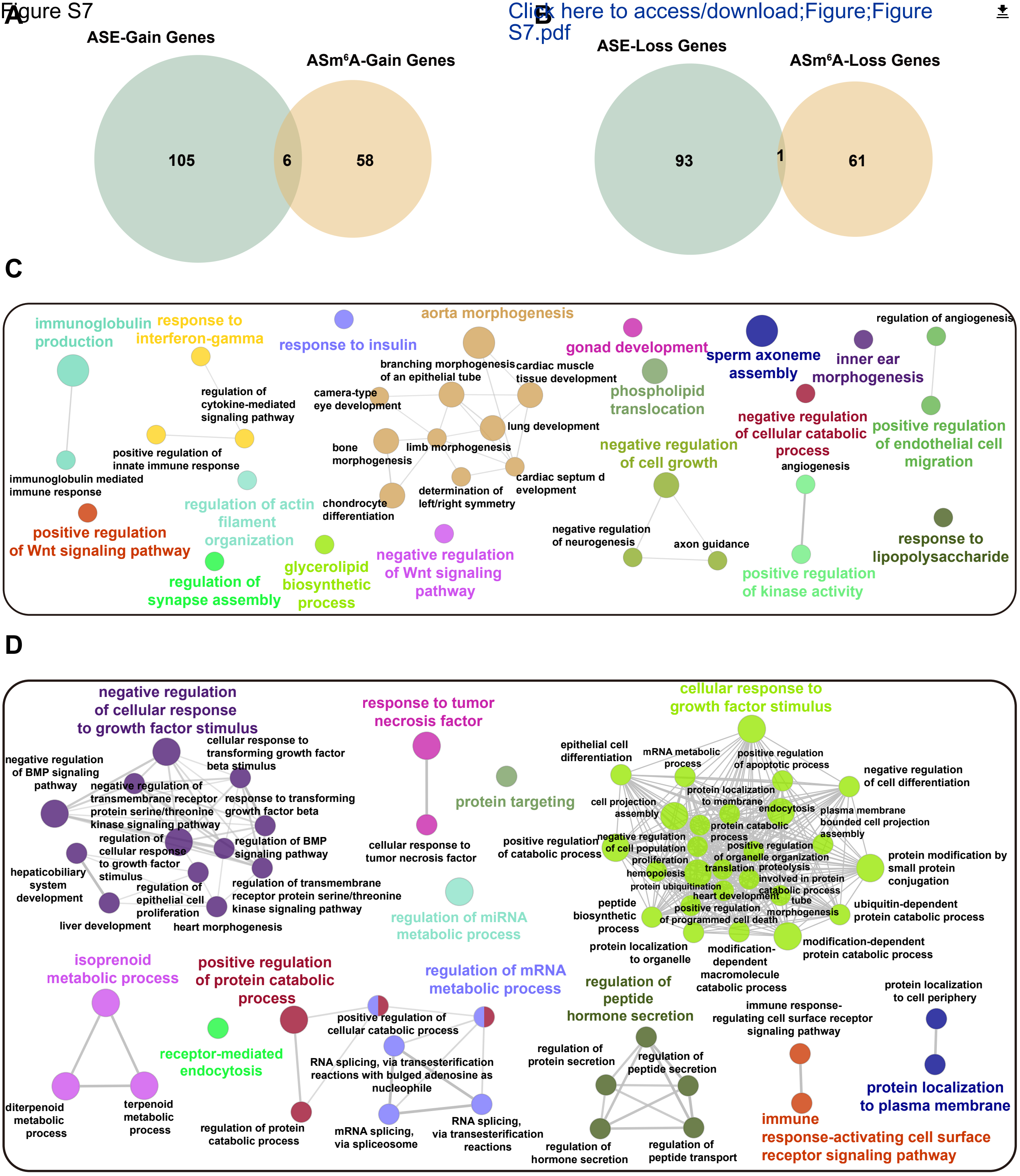

Figure S8

Click here to access/download;Figure;Figure S8.pdf

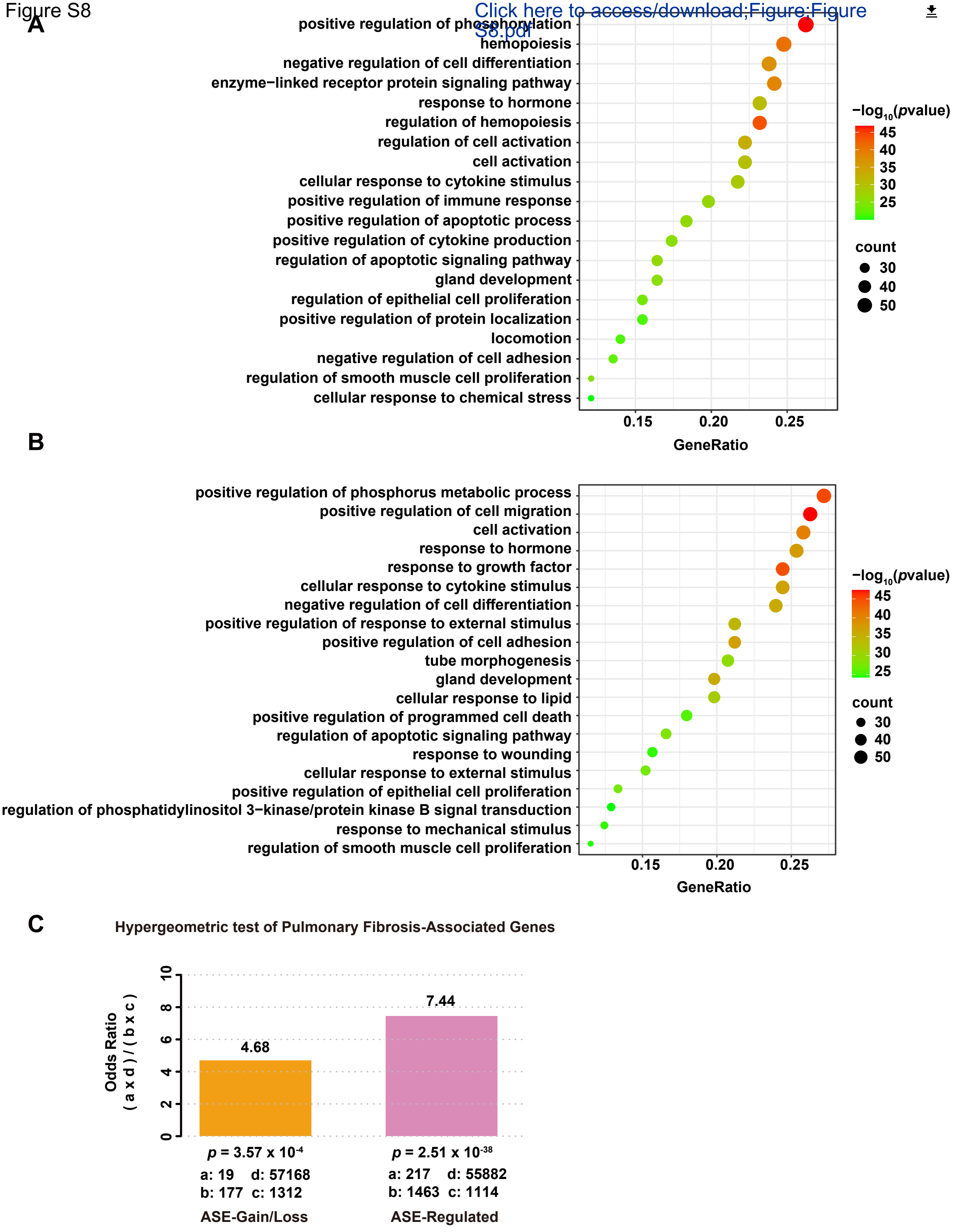

Figure S9

Click here to access/download;Figure;Figure

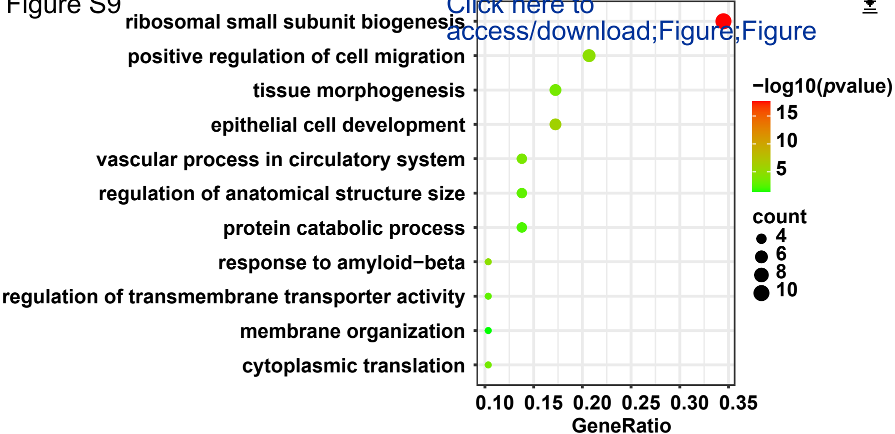

Figure S10

Click here to access/download;Figure;Figure S10.pdf

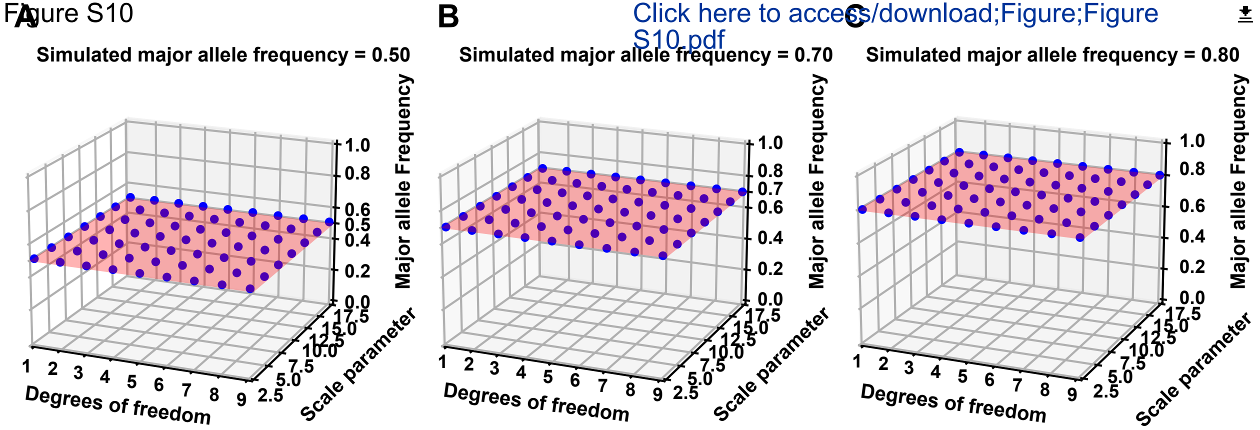

Additonal file 1

Click here to access/download
Supplementary Material
Additonal file 1_revise.docx

Additonal file 2 Table S1

Click here to access/download
Supplementary Material
Additional_File_2_Table_S1.csv

Additonal file 2 Table S2

Click here to access/download
Supplementary Material
Additional_File_2_Table_S2.csv

Additonal file 2 Table S3

Click here to access/download
Supplementary Material
Additional_File_2_Table_S3.csv

Additonal file 2 Table S4

Click here to access/download
Supplementary Material
Additional_File_2_Table_S4.csv

Additonal file 2 Table S5

Click here to access/download
Supplementary Material
Additional_File_2_Table_S5.csv

Additonal file 2 Table S6

Click here to access/download
Supplementary Material
Additional_File_2_Table_S6.csv

Additonal file 2 Table S7

Click here to access/download
Supplementary Material
Additional_File_2_Table_S7.csv

Additonal file 2 Table S8

Click here to access/download
Supplementary Material
Additional_File_2_Table_S8.csv

Response to Reviewers Click here to access/download;Personal Cover;Response to Reviewers_revise.docx

January 6, 2024

Editorial Office of GigaScience

Dear Dr. Hans Zauner,

We are grateful for the prompt review of our manuscript, and helpful comments from two reviewers. According to the reviewers' comments, we have supplemented and improved several aspects of the manuscript. Specifically, we provided a more detailed explanation of the methodology, emphasized the applicability of M6Allele to various types of RNA modifications, and clarified the suitability of different peak-calling tools. In addition, we conducted MeRIP-seq and Sanger sequencing to experimentally verify ASm6A events predicted by M6Allele, evaluated the robustness of the methods through statistical significance tests and variability analyses, assessed the impact of the pseudo-phasing strategy on detection accuracy, and provided additional discussions on the effects of key parameters. Furthermore, we conducted additional comparisons of computational time across different tools. We also replaced and supplemented some IGV examples to present the analysis results more intuitively. Additionally, the software "M6Allele" was approved by SciCrunch.org, with the RRID number SCR_026077, and its bio.tools ID is biotools:m6allele. The workflow has also been registered on WorkflowHub, and the DOI is https://doi.org/10.48546/WORKFLOWHUB.WORKFLOW.1223.1.

Finally, the newly generated MeRIP-seq data and the raw results of Sanger sequencing have been uploaded to GigaDB for public access (files.gigadb.org/M6Aelle). Additionally, the MeRIP-seq data have also been deposited in the Genome Sequence Archive (GSA) under the accession ID HRA009897.

The manuscript was revised based on the reviewer's comments and all the revised texts were marked in red. Please find the point-by-point response below.

Thanks for your patience and coordination. I look forward to hearing from you.

Sincerely,

Xiaotong Luo, PhD

Point-by-point responses

Reviewer #1:

1. The method is entitled with "m6A"; however, it seems to me that the developed framework is equally applicable to other RNA modifications that can be profiled with MeRIP-seq technique, such as, m7G and Ac4C. It is possible to include another case study of other modifications. It can be very interesting and substantially expand the coverage of the proposed framework.

Response: Thank you for this valuable comment. Indeed, while focusing on identifying allele-specific modifications (ASM) of m6A in MeRIP-Seq experiments, M6Allele is equally applicable to other types of RNA modifications detected through antibody enrichment followed by sequencing. Since M6Allele primarily analyzes data based on peak information and SNP sites, it can process any modification data with well-defined peaks and SNPs detected via MeRIP-seq or similar approaches.

We have clarified this point in the manuscript, with the following statement added around line 447: *"Although M6Allele was originally designed for MeRIP-seq experiments, it is also applicable for peak detection and differential analysis of other RIP-seq data, such as m7G or Ac4C. However, since the peak-calling tools within the M6Allele pipeline are primarily optimized for MeRIP-seq data, users can alternatively upload peak-calling results from other tools to facilitate the analysis of ASM events across various RIP-seq datasets."*

2. MetPeak is recommended as the peak calling method of the framework, which is a little bit surprising to me, as recent studies recommended TRESS and exomePeak2. Are there any particular reasons that MetPeak was recommended, and are other peak calling tools equally suitable?

Response: Thank you for your insightful comment. Indeed, recent tools such as TRESS and exomePeak2 have demonstrated strong performance in peak calling for MeRIP-Seq data, particularly in terms of sensitivity and specificity. In our study, we opted to use MetPeak primarily because it had been successfully applied in our previous studies, RM2Target[1], RMVar[2], and m6AVar[3], making it a natural choice for integration into the pipeline. However, the framework is flexible and also supports other peak-calling tools, including TRESS, exomePeak2, and MACS3.

To address this point, we have added a clarification in the manuscript (line 645), stating: "*While MeTPeak was used as the default peak-calling tool in this study, we have tested other peak-calling tools, such as TRESS and exomePeak2, and MACS3, and confirmed that they are also compatible with M6Allele.*"

Reviewer #2:

1. Authors should validate some called candidate sites, e.g., using MeRIP-seq + Sanger sequencing.

Response: Thank you for your insightful suggestion. To address this point, we conducted experimental validations of candidate ASm6A sites using MeRIP-seq followed by Sanger sequencing on RNA samples derived from the human monocytic THP-1 cell line. Initially, we selected 15 positive and 15 negative candidate ASm6A sites based on M6Allele's predictions. To ensure that the expression levels of the transcripts at the selected sites were adequate for Sanger sequencing, we further examined the total read counts for each site in the Input samples. Sites with fewer than 25 reads were excluded, resulting in 6 positive and 14 negative sites being retained for Sanger sequencing (Additional file 2: Tables S4). Using EditR software to analyze the Sanger sequencing chromatograms, we calculated the proportions of different nucleotides at the selected sites in both the IP and input samples, determining the odds ratio for the major allele[4]. Using an odds ratio greater than 1.2 as the threshold, we classified 9 sites as true positives and 11 as true negatives for ASm6A (Additional file 2: Tables S4). Using these 20 sites, we compared the performance of three ASm6A detection tools. The results indicated that M6Allele achieved a higher AUC compared to the other tools (Fig. 3g), emphasizing its superior accuracy in detecting ASm6A events.

To clarify this point, we have added a detailed explanation in the manuscript (line 265), stating: "*To further validate M6Allele's ASm6A detection performance, we conducted experimental validations using MeRIP-seq (GSA-Human: HRA009897) on the human monocytic THP-1 cell line, followed by Sanger sequencing. A total of 20 candidate sites were selected based on predictions from M6Allele (Additional file 2: Table S2, Table S3, Table S4). We utilized EditR software to analyze the Sanger sequencing chromatograms, calculating the ratios of different nucleotides at the selected sites in both the IP and input samples, and determining the odds ratio for the major allele. Using a threshold of greater than 1.2 for the odds ratio, we classified 9 ASm6A sites as true positives and 11 as true negatives. We then compared the performance of three ASm6A detection tools using these 20 sites. The results demonstrated that M6Allele achieved a significantly higher AUC compared to the other two tools (Fig. 3g).*"

Additionally, in the Methods section (line 721), we have added the following details to clarify the process of selecting and validating candidate sites: "*To validate potential ASm6A events, we initially selected 15 positive and 15 negative candidate sites based on M6Allele's predictions from the THP-1 cell line MeRIP-seq data. Ensure that the expression levels of the transcripts at the selected sites are sufficient for the validation, we filtered the sites by calculating the total read counts in the Input samples and excluded sites with fewer than 25 reads (Additional file 2: Table S4). As a result, a total of 6 positive and 14 negative sites were used for validation. Primers targeting these sites were designed for both Input and IP samples, with detailed primer sequences provided in Additional file 2: Table S3. PCR products were gel-purified and subjected to Sanger sequencing. The sequencing chromatograms were processed using EditR software to calculate the proportions of different nucleotides at the selected sites. Odds ratios for the major allele were calculated to compare nucleotide proportions between IP and input samples, with sites having an odds ratio greater than 1.2 classified as positive ASm6A events.*"

2. Fig. 2 The authors should conduct a larger number of simulations and report error bars (or

confidence intervals).

Response: Thank you for the great comment. To address this point, we conducted the 50 simulation datasets and added error bars to Fig. 2, representing standard deviations, to reflect the variability in the results.

3. Why weren't the performance metrics used in Fig. 2 not used to compare M6Allele to the other two tools for ASm6A?

Response: Thank you for this great suggestion. To address this concern, we compared the performance of M6Allele with two other ASm6A tools using the same metrics as ASE. We specifically analyzed the impact of five factors: read length, library size, FPKM, the number of SNPs per peak, and the number of biological replicates on the performance of the three tools. The results showed that M6Allele outperformed the other tools in terms of F0.5 and F1 scores, which combine precision and recall. Additionally, M6Allele exhibited a lower false discovery rate, highlighting its superior performance. These findings indicate that M6Allele has a clear advantage over the other tools in both ASE and ASM detection tasks.

To clarify this point, we have added a detailed explanation in the manuscript (line 244), stating: "*To provide a more comprehensive evaluation, we further compared the detailed performance metrics of different ASm6A detection algorithms (Additional file 1: Fig. S3). The results showed that M6Allele consistently outperformed the other two tools across all categories, combining higher precision and recall while maintaining a lower false discovery rate. Notably, M6Allele exhibited smaller fluctuations and superior stability compared to the other two tools, especially under challenging conditions such as lower library sizes or fewer biological replicates.*"

4. Fig. 3 Authors should show whether increases in each category produce statistically different changes. For example, does increasing number of biological replicates from 1 to 3 result in a statistically significant drop in average error?

Response: Thank you for your insightful comment. To address this, we conducted statistical analyses to evaluate whether changes in five key variables affect performance metrics (Fig. 3). The results showed that increasing read length did not have a significant impact on M6Allele's performance, indicating robustness to sequencing read length (Fig. 3a). However, increasing library size, FPKM, the number of SNPs per peak, and the number of biological replicates led to a significant reduction in average error rates (Fig. 3b-e). Notably, increasing the number of biological replicates from 1 to 3 led to a gradual and statistically significant decrease in average error rates, which then stabilized. These findings underscored the ability of M6Allele to effectively utilize high-quality datasets and suggest that experimental designs incorporating sufficient library size and at least three biological replicates can achieve satisfactory performance.

To clarify this point, we have added a detailed explanation in the manuscript (line 215), stating: "*The results demonstrated that changes in sequencing read length do not affect the performance*

of M6Allele (Fig. 3a). However, increases in library size, FPKM, the number of SNPs in a peak, and the number of biological replicates led to a reduction in the average error rate, with particularly pronounced improvements observed for greater library depth and higher gene expression levels (Fig. 3b-e)."

5. Fig. S2 shows very little differences (if any) in performance of a given tool with an increase in any of the categories. For example, increasing number of biological replicates has no effect on performance of any of the tools. What is going on here?

Response: Thank you for your insightful comment. Fig. S2 compares the AUC-ROC of M6Allele and the other two tools under five key variables. While AUC-ROC focuses solely on sensitivity and specificity, merely providing an overview of performance across various thresholds, it does not capture the detailed performance variations that may occur under specific conditions To address this, we calculated additional performance metrics, including precision, recall, F0.5, F1, and FDR (Additional file 1: Fig. S3). These analyses revealed that M6Allele consistently improved with increasing library size, SNP numbers, and biological replicates, outperforming the other two tools in terms of smaller fluctuations and superior stability. For example, with three or more biological replicates, M6Allele exhibited lower error rates and more consistent performance compared to the other tools, even under challenging conditions such as smaller library sizes.

6. Authors should run M6Allele and the two other tools on more real datasets and compare the results.

Response: Thank you for your insightful comment. As mentioned in our response to Comment 1, we performed MeRIP-seq and Sanger sequencing validations using RNA samples from the human monocytic THP-1 cell line. These validations included 20 candidate ASm6A sites derived from M6Allele predictions, along with corresponding results from two other tools. Using Sanger sequencing as the ground truth, we assessed each tool's performance, and M6Allele achieved a significantly higher AUC (0.7778) compared to ASPRIN (0.5455) and the algorithm developed by *Cao S* et al. (0.5707) (Fig. 3g). These findings highlight M6Allele's ability to outperform other tools in accurately detecting ASm6A modifications.

7. Can the authors evaluate phasing accuracy in simulation studies?

Response: For haplotyping, we referred to the voting-based pseudo-phasing strategy used in MBASED[5], which has demonstrated the feasibility of this approach. To further evaluate the impact of the pseudo-phasing strategy on ASm6A detection, we compared the performance of M6Allele using known phasing information versus the pseudo-phasing strategy. The results (Fig. 3f) showed comparable AUCs, with 0.9216 for known phasing and 0.9039 for the pseudo-phasing strategy. These findings indicate that the pseudo-phasing approach employed by M6Allele is sufficiently accurate to support reliable ASm6A detection.

To clarify this in the manuscript, we have added the following description at line 235:

"*Furthermore, we also investigated the impact of the pseudo-phasing strategy on the performance of M6Allele. The results indicated that the AUC of M6Allele was 0.9216 with known phasing information, which is comparable to the AUC of 0.9039 obtained with pseudo-phasing strategy (Fig. 3f). This further validates the reliability of the pseudo-phasing method in ASm6A detection.*"

8. Computational time should be compared between M6Allele and the other two mentioned approaches using different number of samples.

Response: Thank you for this valuable comment. To address this, we compared the computational time of M6Allele with the two other tools for both ASE and ASM detection tasks. The comparison was conducted using the real datasets mentioned in the manuscript (GSE164151 and GSE198288), which include a total of 12 samples. We evaluated the time cost across five gradients of sample sizes under single-threaded mode for all tools. For ASE detection, the computational speed of M6Allele was comparable to geneiASE, and both were significantly faster than MBASED, with this advantage becoming more pronounced as the sample size increased (Additional file 1: Fig. S4a). For ASM detection, M6Allele achieved intermediate performance, being slower than *Cao S* et al.'s tool but faster than ASPRIN (Additional file 1: Fig. S4b). *Cao S* et al.'s tool demonstrated superior speed, particularly for larger sample sizes, which may be partially attributed to differences in the handling of SNP information and statistical modeling strategies. M6Allele performs more comprehensive integration of each SNP information within peak, which could increase computational complexity while improving detection accuracy.

To clarify this in the manuscript, we have added the following description at line 255: "*Additionally, we evaluated the computational time of M6Allele in comparison to two other tools for both ASE and ASm6A detection tasks. Using two publicly available MeRIP-seq datasets from GEO database (GSE164151 and GSE198288) with a total of 12 human samples, we tested computational efficiency across five sample size gradients under single-threaded mode. The results showed that M6Allele exhibited comparable speed to geneiASE for ASE detection and intermediate performance for ASm6A detection, being slower than Cao S et al.'s tool but faster than ASPRIN (Additional file 1: Fig. S4). This difference in speed may be attributed to M6Allele's more comprehensive integration of SNP information within peaks, which increases computational complexity while ensuring higher detection accuracy.*"

9. The total number of reads in IP and input for Fig. S3 A and B are very similar to me. Seems like these should not be called as m6A peaks to begin with.

Response: Thank you for pointing out this observation. We agree that the peaks shown in Fig. S5A and S5B (previously S3A and S3B) may not be ideal examples of m6A peaks due to the similar number of reads in IP and input samples. This could be attributed to certain aspects of the peak-calling algorithm, which may occasionally lead to the identification of peaks with less distinct enrichment. To address this, we have replaced these examples with more representative peaks that exhibit a clearer enrichment in IP over input.

10. Authors should show IGV examples of differential allele-specific m6A.

Response: Thank you for your suggestion. To address this, we have added IGV examples illustrating differential allele-specific m6A modifications. These examples have been included in the revised supplementary materials as Fig. S6.

Additionally, we have incorporated the following description at line 288 in the manuscript: "*To illustrate these four ASm6A categories, we provided IGV visualizations of randomly selected examples for each category (Additional file 1: Fig. S6). The observed haplotype distributions in IP and Input samples were consistent with M6Allele's detection results for differential ASm6A events.*"

Reference

1. Bao X, Zhang Y, Li H, Teng Y, Ma L, Chen Z, Luo X, Zheng J, Zhao A, Ren J, Zuo Z. RM2Target: a comprehensive database for targets of writers, erasers and readers of RNA modifications. Nucleic Acids Res. 2023; 51:D269-D279.
2. Luo X, Li H, Liang J, Zhao Q, Xie Y, Ren J, Zuo Z. RMVar: an updated database of functional variants involved in RNA modifications. Nucleic Acids Research. 2021; 49:D1405-D1412.
3. Zheng Y, Nie P, Peng D, He Z, Liu M, Xie Y, Miao Y, Zuo Z, Ren J. m6AVar: a database of functional variants involved in m6A modification. Nucleic Acids Res. 2018; 46:D139-D145.
4. Kluesner MG, Nedveck DA, Lahr WS, Garbe JR, Abrahante JE, Webber BR, Moriarity BS. EditR: A Method to Quantify Base Editing from Sanger Sequencing. CRISPR J. 2018; 1:239-250.
5. Mayba O, Gilbert HN, Liu J, Haverty PM, Jhunjhunwala S, Jiang Z, Watanabe C, Zhang Z. MBASED: allele-specific expression detection in cancer tissues and cell lines. Genome Biol. 2014; 15:405.
