# Supplementary material for: M6Allele: a toolkit for detection of allele-specific RNA N6-methyladenosine modifications
Source: Gigascience. 2025 May 19;14:giaf040. doi: 10.1093/gigascience/giaf040 (PMC12087454; doi:10.1093/gigascience/giaf040)
Supplement: giaf040_Authors_Response_To_Reviewer_Comments_original_submission [file giaf040_authors_response_to_reviewer_comments_original_submission.pdf]

Author's Response To Reviewer Comments

January 6, 2024

Editorial Office of GigaScience

Dear Dr. Hans Zauner,

We are grateful for the prompt review of our manuscript, and helpful comments from two reviewers. According to the reviewers' comments, we have supplemented and improved several aspects of the manuscript. Specifically, we provided a more detailed explanation of the methodology, emphasized the applicability of M6Allele to various types of RNA modifications, and clarified the suitability of different peak-calling tools. In addition, we conducted MeRIP-seq and Sanger sequencing to experimentally verify ASm6A events predicted by M6Allele, evaluated the robustness of the methods through statistical significance tests and variability analyses, assessed the impact of the pseudo-phasing strategy on detection accuracy, and provided additional discussions on the effects of key parameters. Furthermore, we conducted additional comparisons of computational time across different tools. We also replaced and supplemented some IGV examples to present the analysis results more intuitively. Additionally, the software “M6Allele” was approved by SciCrunch.org, with the RRID number SCR_026077, and its bio.tools ID is biotools:m6allele. The workflow has also been registered on WorkflowHub, and the DOI is https://doi.org/10.48546/WORKFLOWHUB.WORKFLOW.1223.1.

Finally, the newly generated MeRIP-seq data and the raw results of Sanger sequencing have been uploaded to GigaDB for public access (files.gigadb.org/M6Aelle). Additionally, the MeRIP-seq data have also been deposited in the Genome Sequence Archive (GSA) under the accession ID HRA009897.

The manuscript was revised based on the reviewer’s comments and all the revised texts were marked in red. Please find the point-by-point response below.

Thanks for your patience and coordination. I look forward to hearing from you.

Sincerely,

Xiaotong Luo, PhD

Point-by-point responses

Reviewer #1:
1. The method is entitled with "m6A"; however, it seems to me that the developed framework is equally applicable to other RNA modifications that can be profiled with MeRIP-seq technique, such as, m7G and Ac4C. It is possible to include another case study of other modifications. It can be very interesting and substantially expand the coverage of the proposed framework.

Response: Thank you for this valuable comment. Indeed, while focusing on identifying allele-specific modifications (ASM) of m6A in MeRIP-Seq experiments, M6Allele is equally applicable to other types of RNA modifications detected through antibody enrichment followed by sequencing. Since M6Allele primarily analyzes data based on peak information and SNP sites, it can process any modification data with well-defined peaks and SNPs detected via MeRIP-seq or similar approaches.

We have clarified this point in the manuscript, with the following statement added around line 447: “Although M6Allele was originally designed for MeRIP-seq experiments, it is also applicable for peak detection and differential analysis of other RIP-seq data, such as m7G or Ac4C. However, since the peak-calling tools within the M6Allele pipeline are primarily optimized for MeRIP-seq data, users can alternatively upload peak-calling results from other tools to facilitate the analysis of ASM events across various RIP-seq datasets.”

2. MetPeak is recommended as the peak calling method of the framework, which is a little bit surprising to me, as recent studies recommended TRESS and exomePeak2. Are there any particular reasons that MetPeak was recommended, and are other peak calling tools equally suitable?

Response: Thank you for your insightful comment. Indeed, recent tools such as TRESS and exomePeak2 have demonstrated strong performance in peak calling for MeRIP-Seq data, particularly in terms of sensitivity and specificity. In our study, we opted to use MetPeak primarily because it had been successfully applied in our previous studies, RM2Target[1], RMVar[2], and m6AVar[3], making it a natural choice for integration into the pipeline. However, the framework is flexible and also supports other peak-calling tools, including TRESS, exomePeak2, and MACS3.

To address this point, we have added a clarification in the manuscript (line 645), stating: "While MeTPeak was used as the default peak-calling tool in this study, we have tested other peak-calling tools, such as TRESS and exomePeak2, and MACS3, and confirmed that they are also compatible with M6Allele."

Reviewer #2:
1. Authors should validate some called candidate sites, e.g., using MeRIP-seq + Sanger sequencing.

Response: Thank you for your insightful suggestion. To address this point, we conducted experimental validations of candidate ASm6A sites using MeRIP-seq followed by Sanger sequencing on RNA samples derived from the human monocytic THP-1 cell line. Initially, we selected 15 positive and 15 negative candidate ASm6A sites based on M6Allele's predictions. To ensure that the expression levels of the transcripts at the selected sites were adequate for Sanger sequencing, we further examined the total read counts for each site in the Input samples. Sites with fewer than 25 reads were excluded, resulting in 6 positive and 14 negative sites being retained for Sanger sequencing (Additional file 2: Tables S4). Using EditR software to analyze the Sanger sequencing chromatograms, we calculated the proportions of different nucleotides at the selected sites in both the IP and input samples, determining the odds ratio for the major allele[4]. Using an odds ratio greater than 1.2 as the threshold, we classified 9 sites as true positives and 11 as true negatives for ASm6A (Additional file 2: Tables S4). Using these 20 sites, we compared the performance of three ASm6A detection tools. The results indicated that M6Allele achieved a higher AUC compared to the other tools (Fig. 3g), emphasizing its superior accuracy in detecting ASm6A events.

To clarify this point, we have added a detailed explanation in the manuscript (line 265), stating:
"To further validate M6Allele's ASm6A detection performance, we conducted experimental validations using MeRIP-seq (GSA-Human: HRA009897) on the human monocytic THP-1 cell line, followed by Sanger sequencing. A total of 20 candidate sites were selected based on predictions from M6Allele (Additional file 2: Table S2, Table S3, Table S4). We utilized EditR software to analyze the Sanger sequencing chromatograms, calculating the ratios of different nucleotides at the selected sites in both the IP and input samples, and determining the odds ratio for the major allele. Using a threshold of greater than 1.2 for the odds ratio, we classified 9 ASm6A sites as true positives and 11 as true negatives. We then compared the performance of three ASm6A detection tools using these 20 sites. The results demonstrated that M6Allele achieved a significantly higher AUC compared to the other two tools (Fig. 3g)."

Additionally, in the Methods section (line 721), we have added the following details to clarify the process of selecting and validating candidate sites: "To validate potential ASm6A events, we initially selected 15 positive and 15 negative candidate sites based on M6Allele's predictions from the THP-1 cell line MeRIP-seq data. Ensure that the expression levels of the transcripts at the selected sites are sufficient for the validation, we filtered the sites by calculating the total read counts in the Input samples and excluded sites with fewer than 25 reads (Additional file 2: Table S4). As a result, a total of 6 positive and 14 negative sites were used for validation. Primers targeting these sites were designed for both Input and IP samples, with detailed primer sequences provided in Additional file 2: Table S3. PCR products were gel-purified and subjected to Sanger sequencing. The sequencing chromatograms were processed using EditR software to calculate the proportions of different nucleotides at the selected sites. Odds ratios for the major allele were calculated to compare nucleotide proportions between IP and input samples, with sites having an odds ratio greater than 1.2 classified as positive ASm6A events."

2. Fig. 2 The authors should conduct a larger number of simulations and report error bars (or confidence intervals).

Response: Thank you for the great comment. To address this point, we conducted the 50 simulation datasets and added error bars to Fig. 2, representing standard deviations, to reflect the variability in the results.

3. Why weren't the performance metrics used in Fig. 2 not used to compare M6Allele to the other two tools for ASm6A?

Response: Thank you for this great suggestion. To address this concern, we compared the performance of M6Allele with two other ASm6A tools using the same metrics as ASE. We specifically analyzed the impact of five factors: read length, library size, FPKM, the number of SNPs per peak, and the number of biological replicates on the performance of the three tools. The results showed that M6Allele outperformed the other tools in terms of F0.5 and F1 scores, which combine precision and recall. Additionally, M6Allele exhibited a lower false discovery rate, highlighting its superior performance. These findings indicate that M6Allele has a clear advantage over the other tools in both ASE and ASM detection tasks.

To clarify this point, we have added a detailed explanation in the manuscript (line 244), stating: "To provide a more comprehensive evaluation, we further compared the detailed performance metrics of different ASm6A detection algorithms (Additional file 1: Fig. S3). The results showed that M6Allele consistently outperformed the other two tools across all categories, combining higher precision and recall while maintaining a lower false discovery rate. Notably, M6Allele exhibited smaller fluctuations and superior stability compared to the other two tools, especially under challenging conditions such as lower library sizes or fewer biological replicates."

4. Fig. 3 Authors should show whether increases in each category produce statistically different changes. For example, does increasing number of biological replicates from 1 to 3 result in a statistically significant drop in average error?

Response: Thank you for your insightful comment. To address this, we conducted statistical analyses to evaluate whether changes in five key variables affect performance metrics (Fig. 3). The results showed that increasing read length did not have a significant impact on M6Allele's performance, indicating robustness to sequencing read length (Fig. 3a). However, increasing library size, FPKM, the number of SNPs per peak, and the number of biological replicates led to a significant reduction in average error rates (Fig. 3b-e). Notably, increasing the number of biological replicates from 1 to 3 led to a gradual and statistically significant decrease in average error rates, which then stabilized. These findings underscored the ability of M6Allele to effectively utilize high-quality datasets and suggest that experimental designs incorporating sufficient library size and at least three biological replicates can achieve satisfactory performance.

To clarify this point, we have added a detailed explanation in the manuscript (line 215), stating: "The results demonstrated that changes in sequencing read length do not affect the performance of M6Allele (Fig. 3a). However, increases in library size, FPKM, the number of SNPs in a peak, and the number of biological replicates led to a reduction in the average error rate, with particularly pronounced improvements observed for greater library depth and higher gene expression levels (Fig. 3b-e)."

5. Fig. S2 shows very little differences (if any) in performance of a given tool with an increase in any of the categories. For example, increasing number of biological replicates has no effect on performance of any of the tools. What is going on here?

Response: Thank you for your insightful comment. Fig. S2 compares the AUC-ROC of M6Allele and the other two tools under five key variables. While AUC-ROC focuses solely on sensitivity and specificity, merely providing an overview of performance across various thresholds, it does not capture the detailed performance variations that may occur under specific conditions To address this, we calculated additional performance metrics, including precision, recall, F0.5, F1, and FDR (Additional file 1: Fig. S3). These

analyses revealed that M6Allele consistently improved with increasing library size, SNP numbers, and biological replicates, outperforming the other two tools in terms of smaller fluctuations and superior stability. For example, with three or more biological replicates, M6Allele exhibited lower error rates and more consistent performance compared to the other tools, even under challenging conditions such as smaller library sizes.

6. Authors should run M6Allele and the two other tools on more real datasets and compare the results.

Response: Thank you for your insightful comment. As mentioned in our response to Comment 1, we performed MeRIP-seq and Sanger sequencing validations using RNA samples from the human monocytic THP-1 cell line. These validations included 20 candidate ASm6A sites derived from M6Allele predictions, along with corresponding results from two other tools. Using Sanger sequencing as the ground truth, we assessed each tool's performance, and M6Allele achieved a significantly higher AUC (0.7778) compared to ASPRIN (0.5455) and the algorithm developed by Cao S et al. (0.5707) (Fig. 3g). These findings highlight M6Allele's ability to outperform other tools in accurately detecting ASm6A modifications.

7. Can the authors evaluate phasing accuracy in simulation studies?

Response: For haplotyping, we referred to the voting-based pseudo-phasing strategy used in MBASED[5], which has demonstrated the feasibility of this approach. To further evaluate the impact of the pseudo-phasing strategy on ASm6A detection, we compared the performance of M6Allele using known phasing information versus the pseudo-phasing strategy. The results (Fig. 3f) showed comparable AUCs, with 0.9216 for known phasing and 0.9039 for the pseudo-phasing strategy. These findings indicate that the pseudo-phasing approach employed by M6Allele is sufficiently accurate to support reliable ASm6A detection.

To clarify this in the manuscript, we have added the following description at line 235:
"Furthermore, we also investigated the impact of the pseudo-phasing strategy on the performance of M6Allele. The results indicated that the AUC of M6Allele was 0.9216 with known phasing information, which is comparable to the AUC of 0.9039 obtained with pseudo-phasing strategy (Fig. 3f). This further validates the reliability of the pseudo-phasing method in ASm6A detection."

8. Computational time should be compared between M6Allele and the other two mentioned approaches using different number of samples.

Response: Thank you for this valuable comment. To address this, we compared the computational time of M6Allele with the two other tools for both ASE and ASM detection tasks. The comparison was conducted using the real datasets mentioned in the manuscript (GSE164151 and GSE198288), which include a total of 12 samples. We evaluated the time cost across five gradients of sample sizes under single-threaded mode for all tools. For ASE detection, the computational speed of M6Allele was comparable to geneiASE, and both were significantly faster than MBASED, with this advantage becoming more pronounced as the sample size increased (Additional file 1: Fig. S4a). For ASM detection, M6Allele achieved intermediate performance, being slower than Cao S et al.'s tool but faster than ASPRIN (Additional file 1: Fig. S4b). Cao S et al.'s tool demonstrated superior speed, particularly for larger sample sizes, which may be partially attributed to differences in the handling of SNP information and statistical modeling strategies. M6Allele performs more comprehensive integration of each SNP information within peak, which could increase computational complexity while improving detection accuracy.

To clarify this in the manuscript, we have added the following description at line 255: "Additionally, we evaluated the computational time of M6Allele in comparison to two other tools for both ASE and ASm6A detection tasks. Using two publicly available MeRIP-seq datasets from GEO database (GSE164151 and GSE198288) with a total of 12 human samples, we tested computational efficiency across five sample size gradients under single-threaded mode. The results showed that M6Allele exhibited comparable speed to geneiASE for ASE detection and intermediate performance for ASm6A detection, being slower than Cao S et al.'s tool but faster than ASPRIN (Additional file 1: Fig. S4). This difference in speed may be attributed to M6Allele's more comprehensive integration of SNP information within peaks, which increases computational complexity while ensuring higher detection accuracy."

9. The total number of reads in IP and input for Fig. S3 A and B are very similar to me. Seems like these should not be called as m6A peaks to begin with.

Response: Thank you for pointing out this observation. We agree that the peaks shown in Fig. S5A and S5B (previously S3A and S3B) may not be ideal examples of m6A peaks due to the similar number of reads in IP and input samples. This could be attributed to certain aspects of the peak-calling algorithm, which may occasionally lead to the identification of peaks with less distinct enrichment. To address this, we have replaced these examples with more representative peaks that exhibit a clearer enrichment in IP over input.

10. Authors should show IGV examples of differential allele-specific m6A.

Response: Thank you for your suggestion. To address this, we have added IGV examples illustrating differential allele-specific m6A modifications. These examples have been included in the revised supplementary materials as Fig. S6.

Additionally, we have incorporated the following description at line 288 in the manuscript: "To illustrate these four ASm6A categories, we provided IGV visualizations of randomly selected examples for each category (Additional file 1: Fig. S6). The observed haplotype distributions in IP and Input samples were consistent with M6Allele's detection results for differential ASm6A events."

Reference

1. Bao X, Zhang Y, Li H, Teng Y, Ma L, Chen Z, Luo X, Zheng J, Zhao A, Ren J, Zuo Z. RM2Target: a comprehensive database for targets of writers, erasers and readers of RNA modifications. Nucleic Acids Res. 2023; 51:D269-D279.
2. Luo X, Li H, Liang J, Zhao Q, Xie Y, Ren J, Zuo Z. RMVar: an updated database of functional variants involved in RNA modifications. Nucleic Acids Research. 2021; 49:D1405-D1412.
3. Zheng Y, Nie P, Peng D, He Z, Liu M, Xie Y, Miao Y, Zuo Z, Ren J. m6AVar: a database of functional variants involved in m6A modification. Nucleic Acids Res. 2018; 46:D139-D145.
4. Kluesner MG, Nedveck DA, Lahr WS, Garbe JR, Abrahante JE, Webber BR, Moriarity BS. EditR: A Method to Quantify Base Editing from Sanger Sequencing. CRISPR J. 2018; 1:239-250.
5. Mayba O, Gilbert HN, Liu J, Haverty PM, Jhunjhunwala S, Jiang Z, Watanabe C, Zhang Z. MBASED: allele-specific expression detection in cancer tissues and cell lines. Genome Biol. 2014; 15:405.

Editorial Manager®

Cookie Preference Center

We use cookies which are necessary to make our site work. We may also use additional cookies to analyze, improve and personalize our content and your digital experience. For more information, see our Cookie Policy.

You may choose not to allow some types of cookies. However, blocking some types may impact your experience of our site and the services we are able to offer. See the different category headings below to find out more or change your settings.

Allow all

Manage Consent Preferences

Strictly Necessary Cookies

Always active

These cookies are necessary for the website to function and cannot be switched off in our systems. They are usually only set in response to actions made by you which amount to a request for services, such as setting your privacy preferences, logging in or filling in forms. You can set your browser to block or alert you about these cookies, but some parts of the site will not then work. These cookies do not store any personally identifiable information.

Functional Cookies

These cookies enable the website to provide enhanced functionality and personalisation. They may be set by us or by third party providers whose services we have added to our pages. If you do not allow these cookies then some or all of these services may not function properly.

Performance Cookies

These cookies allow us to count visits and traffic sources so we can measure and improve the performance of our site. They help us to know which pages are the most and least popular and see how visitors move around the site.

Targeting Cookies

These cookies may be set through our site by our advertising partners. They may be used by those companies to build a profile of your interests and show you relevant adverts on other sites. If you do not allow these cookies, you will experience less targeted advertising.

Cookie List

Clear

Apply Cancel

Consent Leg.Interest

Confirm my choices

Powered by onetrust
