# Supplementary material for: M6Allele: a toolkit for detection of allele-specific RNA N6-methyladenosine modifications
Source: Gigascience. 2025 May 19;14:giaf040. doi: 10.1093/gigascience/giaf040 (PMC12087454; doi:10.1093/gigascience/giaf040)
Supplement: giaf040_Reviewer_1_Report_original_submission — Jia Meng -- 9/28/2024 [file giaf040_reviewer_1_report_original_submission.pdf]

**Reviewer Report**

**Title: M6Allele: A toolkit for detection of allele-specific RNA N6-methyladenosine modifications**

**Version: Original Submission** **Date:** 9/28/2024

**Reviewer name: Jia Meng**

**Reviewer Comments to Author:**

The manuscript by Zhang et al presents a nice method for detection of allele-specific RNA m6A methylation from MeRIP-seq data.In general, the manuscript is very well prepared, well structured, nicely presented with adequate technical contents. The figures and tables are of very high quality. The statistical methods and experimental design are rigorous. I am convinced that the manuscript is very high quality.I have two minor comments only for the authors to consider.1. The method is entitled with "m6A"; however, it seems to me that the developed framework is equally applicable to other RNA modifications that can be profiled with MeRIP-seq technique, such as, m7G and Ac4C. It is possible to possible to include another case study of other modifications. It can be very interesting and substantially expand the coverage of the proposed framework.2. MetPeak is recommended as the peak calling method of the framework, which is a little bit surprising to me, as recent studies recommended TRESS and exomePeak2. Are there any particular reasons that Metpeak was recommended, and are other peak calling tools equally suitable?

**Level of Interest**

Please indicate how interesting you found the manuscript: Choose an item.

**Quality of Written English**

Please indicate the quality of language in the manuscript: Choose an item.

**Declaration of Competing Interests**

Please complete a declaration of competing interests, considering the following questions:

- Have you in the past five years received reimbursements, fees, funding, or salary from an organisation that may in any way gain or lose financially from the publication of this manuscript, either now or in the future?
- Do you hold any stocks or shares in an organisation that may in any way gain or lose financially from the publication of this manuscript, either now or in the future?
- Do you hold or are you currently applying for any patents relating to the content of the manuscript?
- Have you received reimbursements, fees, funding, or salary from an organization that holds or has applied for patents relating to the content of the manuscript?

- Do you have any other financial competing interests?
- Do you have any non-financial competing interests in relation to this paper?

If you can answer no to all of the above, write 'I declare that I have no competing interests' below. If your reply is yes to any, please give details below.

I declare that I have no competing interests.

I agree to the open peer review policy of the journal. I understand that my name will be included on my report to the authors and, if the manuscript is accepted for publication, my named report including any attachments I upload will be posted on the website along with the authors' responses. I agree for my report to be made available under an Open Access Creative Commons CC-BY license (http://creativecommons.org/licenses/by/4.0/). I understand that any comments which I do not wish to be included in my named report can be included as confidential comments to the editors, which will not be published.

Choose an item.

To further support our reviewers, we have joined with Publons, where you can gain additional credit to further highlight your hard work (see: https://publons.com/journal/530/gigascience). On publication of this paper, your review will be automatically added to Publons, you can then choose whether or not to claim your Publons credit. I understand this statement.

Yes Choose an item.
