# Supplementary material for: M6Allele: a toolkit for detection of allele-specific RNA N6-methyladenosine modifications
Source: Gigascience. 2025 May 19;14:giaf040. doi: 10.1093/gigascience/giaf040 (PMC12087454; doi:10.1093/gigascience/giaf040)
Supplement: giaf040_Reviewer_2_Report_original_submission — Andrew Shafik -- 10/16/2024 [file giaf040_reviewer_2_report_original_submission.pdf]

Reviewer Report

Title: M6Allele: A toolkit for detection of allele-specific RNA N6-methyladenosine modifications

Version: Original Submission **Date:** 10/16/2024

Reviewer name: Andrew Shafik

Reviewer Comments to Author:

The authors have developed M6Allele to identify allele specific m6A sites from MeRIP-seq data. They utilize MBASED's strategy to include pseudo-phasing approach for SNPs, with allelic imbalance being quantified as the odds ratio of the major allele compared to the minor allele in the m6A IP sample. They compare the performance of M6Allele to two other existing tools. These are my main concerns:1. Authors should validate some called candidate sites, e.g. using MeRIP-seq + Sanger sequencing2. Fig. 2 The authors should conduct a larger number of simulations and report error bars (or confidence intervals).3. Why weren't the performance metrics used in Fig. 2 not used to compare M6Allele to the other two tools for ASm6A?4. Fig. 3 Authors should show whether increases in each category produce statistically different changes. For example does increasing number of biological replicates from 1 to 3 result in a statistically significant drop in average error?5. Fig. S2 shows very little differences (if any) in performance of a given tool with an increase in any of the categories. For example, increasing number of biological replicates has no effect on performance of any of the tools. What is going on here?6. Authors should run M6Allele and the two other tools on more real datasets and compare the results.7. Can the authors evaluate phasing accuracy in simulation studies?8. Computational time should be compared between M6Allele and the other two mentioned approaches using different number of samples.9. The total number of reads in IP and input for Fig. S3 A and B are very similar to me. Seems like these should not be called as m6A peaks to begin with.10. Authors should show IGV examples of differential allele-specific m6A

Level of Interest

Please indicate how interesting you found the manuscript: Choose an item.

Quality of Written English

Please indicate the quality of language in the manuscript: Choose an item.

Declaration of Competing Interests

Please complete a declaration of competing interests, considering the following questions:

- Have you in the past five years received reimbursements, fees, funding, or salary from an organisation that may in any way gain or lose financially from the publication of this manuscript, either now or in the future?

- Do you hold any stocks or shares in an organisation that may in any way gain or lose financially from the publication of this manuscript, either now or in the future?
- Do you hold or are you currently applying for any patents relating to the content of the manuscript?
- Have you received reimbursements, fees, funding, or salary from an organization that holds or has applied for patents relating to the content of the manuscript?
- Do you have any other financial competing interests?
- Do you have any non-financial competing interests in relation to this paper?

If you can answer no to all of the above, write 'I declare that I have no competing interests' below. If your reply is yes to any, please give details below.

I declare that I have no competing interests

I agree to the open peer review policy of the journal. I understand that my name will be included on my report to the authors and, if the manuscript is accepted for publication, my named report including any attachments I upload will be posted on the website along with the authors' responses. I agree for my report to be made available under an Open Access Creative Commons CC-BY license (http://creativecommons.org/licenses/by/4.0/). I understand that any comments which I do not wish to be included in my named report can be included as confidential comments to the editors, which will not be published.

Choose an item.

To further support our reviewers, we have joined with Publons, where you can gain additional credit to further highlight your hard work (see: https://publons.com/journal/530/gigascience). On publication of this paper, your review will be automatically added to Publons, you can then choose whether or not to claim your Publons credit. I understand this statement.

Yes Choose an item.
